# Meta-analysis of genome-wide association studies identifies novel loci that influence cupping and the glaucomatous process

**DOI:** 10.1038/ncomms5883

**Published:** 2014-09-22

**Authors:** Henriët. Springelkamp, René Höhn, Aniket Mishra, Pirro G. Hysi, Chiea-Chuen Khor, Stephanie J. Loomis, Jessica N. Cooke Bailey, Jane Gibson, Gudmar Thorleifsson, Sarah F. Janssen, Xiaoyan Luo, Wishal D. Ramdas, Eranga Vithana, Monisha E. Nongpiur, Grant W. Montgomery, Liang Xu, Jenny E. Mountain, Puya Gharahkhani, Yi Lu, Najaf Amin, Lennart C. Karssen, Kar-Seng Sim, Elisabeth M. van Leeuwen, Adriana I. Iglesias, Virginie J. M. Verhoeven, Michael A. Hauser, Seng-Chee Loon, Dominiek D. G. Despriet, Abhishek Nag, Cristina Venturini, Paul G. Sanfilippo, Arne Schillert, Jae H. Kang, John Landers, Fridbert Jonasson, Angela J. Cree, Leonieke M. E. van Koolwijk, Fernando Rivadeneira, Emmanuelle Souzeau, Vesteinn Jonsson, Geeta Menon, Blue Mountains Eye Study—GWAS group, Paul Mitchell, Jie Jin Wang, Elena Rochtchina, John Attia, Rodney Scott, Elizabeth G. Holliday, Tien-Yin Wong, Paul N. Baird, Jing Xie, Michael Inouye, Ananth Viswanathan, Xueling Sim, Robert N. Weinreb, Paulus T. V. M. de Jong, Ben A. Oostra, André G. Uitterlinden, Albert Hofman, Sarah Ennis, Unnur Thorsteinsdottir, Kathryn P. Burdon, NEIGHBORHOOD Consortium, R. Rand Allingham, Murray H. Brilliant, Donald L. Budenz, Jessica N. Cooke Bailey, William G. Christen, John Fingert, David S. Friedman, Douglas Gaasterland, Terry Gaasterland, Jonathan L. Haines, Michael A. Hauser, Jae Hee Kang, Peter Kraft, Richard K. Lee, Paul R. Lichter, Yutao Liu, Stephanie J. Loomis, Sayoko E. Moroi, Louis R. Pasquale, Margaret A. Pericak-Vance, Anthony Realini, Julia E. Richards, Joel S. Schuman, William K. Scott, Kuldev Singh, Arthur J. Sit, Douglas Vollrath, Robert N. Weinreb, Janey L. Wiggs, Gadi Wollstein, Donald J. Zack, Kang Zhang, Wellcome Trust Case Control Consortium 2 (WTCCC2), Peter Donnelly (Chair), Ines Barroso (Deputy Chair), Jenefer M. Blackwell, Elvira Bramon, Matthew A. Brown, Juan P. Casas, Aiden Corvin, Panos Deloukas, Audrey Duncanson, Janusz Jankowski, Hugh S. Markus, Christopher G. Mathew, Colin N. A. Palmer, Robert Plomin, Anna Rautanen, Stephen J. Sawcer, Richard C. Trembath, Ananth C. Viswanathan, Nicholas W. Wood, Chris C. A. Spencer, Gavin Band, Céline Bellenguez, Colin Freeman, Garrett Hellenthal, Eleni Giannoulatou, Matti Pirinen, Richard Pearson, Amy Strange, Zhan Su, Damjan Vukcevic, Peter Donnelly, Cordelia Langford, Sarah E. Hunt, Sarah Edkins, Rhian Gwilliam, Hannah Blackburn, Suzannah J. Bumpstead, Serge Dronov, Matthew Gillman, Emma Gray, Naomi Hammond, Alagurevathi Jayakumar, Owen T. McCann, Jennifer Liddle, Simon C. Potter, Radhi Ravindrarajah, Michelle Ricketts, Matthew Waller, Paul Weston, Sara Widaa, Pamela Whittaker, Ines Barroso, Panos Deloukas, Christopher G. Mathew (Chair), Jenefer M. Blackwell, Matthew A. Brown, Aiden Corvin, Chris C. A. Spencer, Timothy D. Spector, Alireza Mirshahi, Seang-Mei Saw, Johannes R. Vingerling, Yik-Ying Teo, Jonathan L. Haines, Roger C. W. Wolfs, Hans G. Lemij, E-Shyong Tai, Nomdo M. Jansonius, Jost B. Jonas, Ching-Yu Cheng, Tin Aung, Ananth C. Viswanathan, Caroline C. W. Klaver, Jamie E. Craig, Stuart Macgregor, David A. Mackey, Andrew J. Lotery, Kari Stefansson, Arthur A. B. Bergen, Terri L. Young, Janey L. Wiggs, Norbert Pfeiffer, Tien-Yin Wong, Louis R. Pasquale, Alex W. Hewitt, Cornelia M. van Duijn, Christopher J. Hammond

**Affiliations:** 1 Department of Ophthalmology, Erasmus Medical Center, Rotterdam 3000 CA, The Netherlands; 2 Department of Epidemiology, Erasmus Medical Center, Rotterdam 3000 CA, The Netherlands; 3 Department of Ophthalmology, University Medical Center Mainz, Mainz 55131, Germany; 4 Department of Genetics and Computational Biology, Statistical Genetics, QIMR Berghofer Medical Research Institute, Royal Brisbane Hospital, Brisbane, Queensland 4006, Australia; 5 Department of Twin Research and Genetic Epidemiology, King’s College London, London WC2R 2LS, UK; 6 Department of Ophthalmology, National University of Singapore and National University Health System, Singapore 119077, Singapore; 7 Division of Human Genetics, Genome Institute of Singapore, Singapore 138672, Singapore; 8 Department of Ophthalmology, Harvard Medical School and Massachusetts Eye and Ear Infirmary, Boston, Massachusetts 02114, USA; 9 Department of Molecular Physiology and Biophysics, Center for Human Genetics Research, Vanderbilt University School of Medicine, Nashville, Tennessee 37232, USA; 10 Department of Epidemiology and Biostatistics, Case Western Reserve University, Cleveland, Ohio 44106, USA; 11 Centre for Biological Sciences, Faculty of Natural and Environmental Sciences, University of Southampton, Southampton SO17 1BJ, UK; 12 deCODE/Amgen, Reykjavik 101, Iceland; 13 Department of Clinical and Molecular Ophthalmogenetics, The Netherlands Institute for Neuroscience (NIN), Royal Netherlands Academy of Arts and Sciences (KNAW), Amsterdam 1105 BA, the Netherlands; 14 Department of Ophthalmology, Duke University Eye Center, Durham, North Carolina 27710, USA; 15 Singapore Eye Research Institute, Singapore National Eye Centre, Singapore 168751, Singapore; 16 Duke-National University of Singapore, Graduate Medical School, Singapore 169857, Singapore; 17 Department of Genetics and Computational Biology, Molecular Epidemiology Laboratory, QIMR Berghofer Medical Research Institute, Royal Brisbane Hospital, Brisbane, Queensland 4006, Australia; 18 Beijing Institute of Ophthalmology, Beijing Tongren Eye Center, Beijing Tongren Hospital, Capital Medical University, Beijing 100730, China; 19 Beijing Ophthalmology and Visual Science Key Lab, Beijing 100730, China; 20 Telethon Institute for Child Health Research, Subiaco, Western Australia 6008, Australia; 21 Departments of Medicine and Ophthalmology, Duke University Medical Center, Durham, North Carolina 27710, USA; 22 UCL Institute of Ophthalmology, London EC1V 9EL, UK; 23 Centre for Eye Research Australia (CERA), University of Melbourne, Royal Victorian Eye and Ear Hospital, Melbourne, Victoria 3002, Australia; 24 Institute of Medical Biometry and Statistics, University of Lübeck, Lübeck 23562, Germany; 25 Department of Medicine, Channing Division of Network Medicine, Brigham and Women's Hospital, Boston, Massachusetts 02115, USA; 26 Department of Ophthalmology, Flinders University, Adelaide, South Australia 5042, Australia; 27 Faculty of Medicine, University of Iceland, Reykjavik 101, Iceland; 28 Department of Ophthalmology, Landspitali National University Hospital, Reykjavik 101, Iceland; 29 Clinical and Experimental Sciences, Faculty of Medicine, University of Southampton, Southampton SO17 1BJ, UK; 30 Department of Internal Medicine, Erasmus Medical Center, Rotterdam 3000 CA, The Netherlands; 31 Netherlands Consortium for Healthy Ageing, Netherlands Genomics Initiative, The Hague 2593 CE, The Netherlands; 32 Department of Ophthalmology, Frimley Park Hospital NHS Foundation Trust, Frimley GU16 7UJ, UK; 33 Department of Ophthalmology and Hamilton Glaucoma Center, University of California, San Diego, California 92093, USA; 34 Department of Retinal Signal Processing, Netherlands Institute for Neuroscience, Amsterdam 1105 BA, The Netherlands; 35 Department of Ophthalmology, Academic Medical Center, Amsterdam 1105 AZ, The Netherlands; 36 Department of Ophthalmology, Leiden University Medical Center, Leiden 2333 ZA, The Netherlands; 37 Department of Clinical Genetics, Erasmus Medical Center, Rotterdam 3000 CA, The Netherlands; 38 Human Development and Health, Faculty of Medicine, University of Southampton, Southampton SO17 1BJ, UK; 39 Saw Swee Hock School of Public Health, National University of Singapore and National University Health System, Singapore 117597, Singapore; 40 Department of Statistics and Applied Probability, National University of Singapore, Singapore 119077, Singapore; 41 Glaucoma Service, The Rotterdam Eye Hospital, Rotterdam 3011 BH, The Netherlands; 42 Department of Medicine, National University of Singapore and National University Health System, Singapore 119077, Singapore; 43 Department of Ophthalmology, University of Groningen, University Medical Center Groningen, Groningen 9700 RB, The Netherlands; 44 Department of Ophthalmology, Medical Faculty Mannheim of the Ruprecht-Karls-University of Heidelberg, Seegartenklinik Heidelberg, Heidelberg 69117, Germany; 45 NIHR Biomedical Research Centre, Moorfields Eye Hospital NHS Foundation Trust and UCL Institute of Ophthalmology, London EC1V 2PD, UK; 46 Centre for Ophthalmology and Visual Science, Lions Eye Institute, University of Western Australia, Perth, Western Australia 6009, Australia; 47 Department of Clinical Genetics, Academic Medical Center, Amsterdam 1105 AZ, The Netherlands; 48 Centre for Vision Research, Department of Ophthalmology and Westmead Millennium Institute, University of Sydney, Sydney, New South Wales, Australia.; 49 University of Newcastle, Newcastle, New South Wales, Australia.; 50 Department of Ophthalmology, Centre for Eye Research Australia, University of Melbourne, Melbourne, Florida, USA.; 51 Walter and Elisa Hall Institute of Medical Research, Melbourne, Victoria, Australia.; 52 NIHR Biomedical Research Centre, Moorfields Eye Hospital NHS Foundation Trust and UCL Institute of Ophthalmology, London, UK.; 53 National University of Singapore, Singapore, Singapore.; 54 Department of Ophthalmology, Duke University Medical Center, Durham, North Carolina, USA.; 55 Center for Human Genetics, Marshfield Clinic Research Foundation, Marshfield,Wisconsin, USA.; 56 Department of Ophthalmology, University of North Carolina, Chapel Hill, North Carolina, USA.; 57 Center for Human Genetics Research, Vanderbilt University School of Medicine, Nashville, Tennessee, USA.; 58 Department of Epidemiology and Biostatistics, Case Western Reserve University, Cleveland, Ohio, USA.; 59 Department of Medicine, Brigham and Women's Hospital, Boston, Massachusetts, USA.; 60 Department of Ophthalmology, College of Medicine, University of Iowa, Iowa City, Iowa, USA.; 61 Department of Anatomy/Cell Biology, College of Medicine, University of Iowa, Iowa City, Iowa, USA.; 62 Wilmer Eye Institute, John Hopkins University, Baltimore, Maryland, USA.; 63 Eye Doctors of Washington, Chevy Chase, Maryland, USA.; 64 Scripps Genome Center, University of California at San Diego, San Diego, California, USA.; 65 Department of Medicine, Duke University Medical Center, Durham, North Carolina, USA.; 66 Channing Division of Network Medicine, Brigham and Women's Hospital, Harvard Medical School, Boston, Massachusetts, USA.; 67 Department of Biostatistics, Harvard School of Public Health, Boston, Massachusetts, USA.; 68 Bascom Palmer Eye Institute, University of Miami Miller School of Medicine, Miami, Florida, USA.; 69 Department of Ophthalmology and Visual Sciences, University of Michigan, Ann Arbor, Michigan, USA.; 70 Department of Ophthalmology, Harvard Medical School and Massachusetts Eye and Ear Infirmary, Boston, Massachusetts, USA.; 71 Institute for Human Genomics, University of Miami Miller School of Medicine, Miami, Florida, USA.; 72 Department of Ophthalmology, WVU Eye Institute, Morgantown, West Virginia, USA.; 73 Department of Ophthalmology, UPMC Eye Center, University of Pittsburgh, Pittsburgh, Pennsylvania, USA.; 74 Department of Ophthalmology, Stanford University, Palo Alto, California, USA.; 75 Department of Ophthalmology, Mayo Clinic, Rochester, Minnesota, USA.; 76 Department of Genetics, Stanford University, Palo Alto, California, USA.; 77 Department of Ophthalmology, Hamilton Eye Center, University of California, San Diego, California, USA.; 78 Wellcome Trust Centre for Human Genetics, University of Oxford, Roosevelt Drive, Oxford OX3 7BN, UK.; 79 Dept Statistics, University of Oxford, Oxford OX1 3TG, UK.; 80 Wellcome Trust Sanger Institute, Wellcome Trust Genome Campus, Hinxton, Cambridge CB10 1SA, UK.; 81 Telethon Institute for Child Health Research, Centre for Child Health Research, University of Western Australia, 100 Roberts Road, Subiaco, Western Australia 6008, Australia.; 82 Cambridge Institute for Medical Research, University of Cambridge School of Clinical Medicine, Cambridge CB2 0XY, UK.; 83 Department of Psychosis Studies, NIHR Biomedical Research Centre for Mental Health at the Institute of Psychiatry, King's College London and The South London and Maudsley NHS Foundation Trust, Denmark Hill, London SE5 8AF, UK.; 84 University of Queensland Diamantina Institute, Brisbane, Queensland, Australia.; 85 Department of Epidemiology and Population Health, London School of Hygiene and Tropical Medicine, London WC1E 7HT, UK and Department of Epidemiology and Public Health, University College London, London WC1E 6BT, UK.; 86 Neuropsychiatric Genetics Research Group, Institute of Molecular Medicine, Trinity College Dublin, Dublin 2, Ireland.; 87 Molecular and Physiological Sciences, The Wellcome Trust, London NW1 2BE, UK.; 88 Department of Oncology, Old Road Campus, University of Oxford, Oxford OX3 7DQ, UK, Digestive Diseases Centre, Leicester Royal Infirmary, Leicester LE7 7HH, UK.; 89 Centre for Digestive Diseases, Queen Mary University of London, London E1 2AD, UK.; 90 Clinical Neurosciences, St George's University of London, London SW17 0RE, UK.; 91 King's College London Department of Medical and Molecular Genetics, King's Health Partners, Guy's Hospital, London SE1 9RT, UK.; 92 Biomedical Research Centre, Ninewells Hospital and Medical School, Dundee DD1 9SY, UK.; 93 King's College London Social, Genetic and Developmental Psychiatry Centre, Institute of Psychiatry, Denmark Hill, London SE5 8AF, UK.; 94 Department of Clinical Neurosciences, University of Cambridge, Addenbrooke's Hospital, Cambridge CB2 0QQ, UK.; 95 Department of Molecular Neuroscience, Institute of Neurology, Queen Square, London WC1N 3BG, UK.

## Abstract

Glaucoma is characterized by irreversible optic nerve degeneration and is the most frequent cause of irreversible blindness worldwide. Here, the International Glaucoma Genetics Consortium conducts a meta-analysis of genome-wide association studies of vertical cup-disc ratio (VCDR), an important disease-related optic nerve parameter. In 21,094 individuals of European ancestry and 6,784 individuals of Asian ancestry, we identify 10 new loci associated with variation in VCDR. In a separate risk-score analysis of five case-control studies, Caucasians in the highest quintile have a 2.5-fold increased risk of primary open-angle glaucoma as compared with those in the lowest quintile. This study has more than doubled the known loci associated with optic disc cupping and will allow greater understanding of mechanisms involved in this common blinding condition.

## Abstract (web summary)

Glaucoma is the most common cause of irreversible blindness worldwide. Here, the authors carry out a large meta-analysis of genetic data from individuals of European and Asian ancestry and identify 10 new loci associated with vertical cup-disc ratio, a key factor in the clinical assessment of patients with glaucoma.

## Graphical abstract

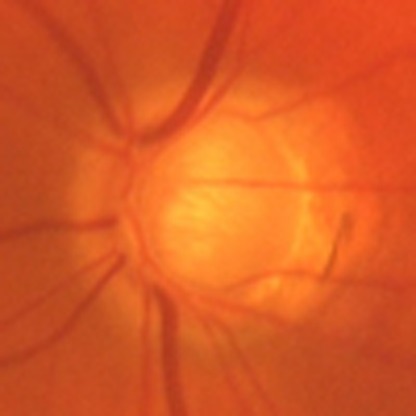

Optic nerve degeneration caused by glaucoma is the most common cause of irreversible blindness worldwide^1^. Glaucomatous optic neuropathy is recognized by changes in the morphology of the optic nerve head, or optic disc, caused by loss of retinal ganglion cells and thinning of the retinal nerve fibre layer. In glaucoma, the nerve fibre layer typically thins in the superior and inferior regions of the nerve creating a vertically elongated depression (the cup). The ratio of the cup to the overall nerve size (the disc), called the vertical cup-disc ratio (VCDR), is a key factor in the clinical assessment and follow-up of patients with glaucoma. VCDR has been shown to be heritable with *h*^*2*^ scores ranging between 0.48 and 0.66^2^,^3^,^4^,^5^,^6^,^7^. At least seven loci have been associated with VCDR in previous genome-wide association studies (GWAS) and three of these were subsequently implicated in primary open-angle glaucoma (POAG)^8^,^9^,^10^,^11^. So far, the explained variance of open-angle glaucoma by age, sex, intraocular pressure and established POAG genes is still small (4–6%)^12^. As with other complex diseases, large sample sizes are needed to ensure sufficient power to fully define the underlying genetic architecture.

Here, we report the largest genome-wide meta-analysis for VCDR, with data from 14 studies from Europe, the United States, Australia and Asia, as part of the International Glaucoma Genetics Consortium. The aim of the study is to identify loci associated with VCDR, and to determine whether these variants are also associated with glaucoma.

We perform the meta-analysis in four stages. In the first stage, we meta-analyse summary data from 10 populations of European ancestry comprising 21,094 individuals. In the second stage, we test the cross-ancestry transferability of the statistically genome-wide-significant associations from the first stage in 6,784 individuals from four Asian cohorts. In the third stage, we examine whether the associations are independent of disc area and/or spherical equivalent. We also combine the genome-wide-significant effects into a genetic risk score and associate this score with the POAG risk in five populations. Finally, we perform gene-based tests and pathway analysis.

We find 10 new loci associated with VCDR, which together increase the risk on POAG 2.5 times. Our findings will help us to unravel the pathogenesis of glaucoma.

## Results

### Meta-analysis of GWAS

**Figure 1:**
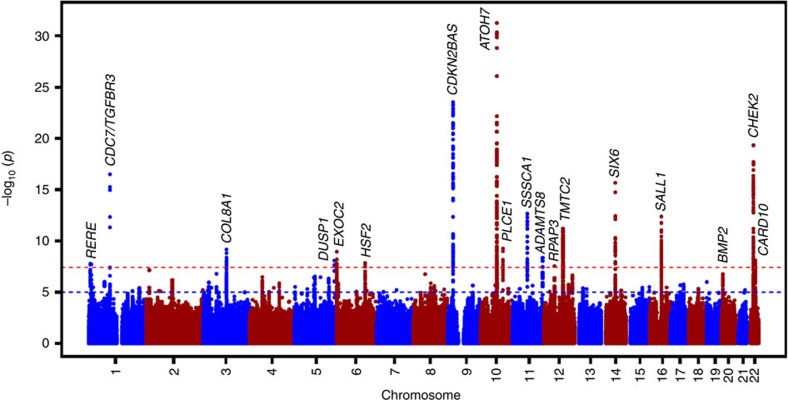
Manhattan plot of the GWAS meta-analysis for vertical cup-disc ratio in the combined analysis (***n*****=27,878).** The plot shows −log_10_-transformed *P* values for all SNPs (*z*-statistic). The red-dotted horizontal line represents the genome-wide significance threshold of *P*<5.0 × 10^−8^; the blue-dotted line indicates *P* value of 1 × 10^−5^.

In stage 1, we analysed ~2.5 million HapMap stage 2 single-nucleotide polymorphisms (SNPs)—either directly genotyped or imputed in 21,094 subjects of European ancestry (Supplementary Fig. 1; Supplementary Table 1; Supplementary Methods). The inflation factors (*λ*) varied between 0.98 and 1.12, implying adequate within-study control of population substructure (Supplementary Table 2; Supplementary Figs 2 and 3). The overall *λ* was 1.05. This analysis yielded 440 genome-wide-significant SNPs (*P*<5.0 × 10^−8^) located across 15 chromosomal regions (Table 1; Supplementary Fig. 4a). In stage 2, we investigated the SNP with the strongest association at each region in the Asian populations and found that eight were nominally significant (*P*<0.05) with an effect in the same direction and generally the same order of magnitude (Table 1; Supplementary Fig. 4b). Five of the seven loci that did not reach nominal significance in those of Asian descent had a similar effect in the same direction. Supplementary Table 3 shows the most significant SNPs in Asians within 100,000 base pairs from the most significant associated SNP in Europeans. Meta-analysis of only the Asian populations did not result in new genome-wide-significant findings. The combined analysis of the European and Asian populations resulted in three additional genome-wide-significant associations on chromosomes 1, 6 and 22 (Table 1; Fig. 1). The level of heterogeneity across the samples are shown in Table 1. Of the 18 genome-wide-significant loci, 10 are novel for the VCDR outcome (*COL8A1*, *DUSP1*, *EXOC2*, *PLCE1*, *ADAMTS8*, *RPAP3*, *SALL1*, *BMP2*, *HSF2* and *CARD10*) (Supplementary Fig. 5). There were no significant differences in terms of allele frequencies across the different cohorts (Supplementary Table 4). The effect estimates from the participating cohorts appear not to be influenced by main demographic characteristics, such as mean age and sex ratio (Supplementary Fig. 6).

### Adjustment for disc area and spherical equivalent

Four of the 18 genome-wide-significant loci have been previously associated with optic disc area (*CDC7/TGFBR3*, *ATOH7*, *SALL1* and *CARD10*)^10^,^13^. Because the size of the optic nerve varies between individuals and is correlated to the VCDR^14^, we adjusted the association to VCDR for optic nerve (disc) area. This resulted in a reduced effect size and significance (*P*=3.48 × 10^−11^ to *P*=9.00 × 10^−3^) at the *CDC7–TGFBR3* locus, suggesting the VCDR association at this locus is explained primarily by its known association with disc area (Supplementary Table 5a–c). A similar reduction in effect was seen for *ATOH7*. However, for this locus there remains a significant disc-area-independent effect (*P*=7.28 × 10^−9^). There was no change in association significance for any of the 10 new loci reported here, suggesting they do not act primarily on disc area.

It is of interest that two genes (*SIX6* and *BMP2*) overlap with those implicated in myopia^15^, an important risk factor for POAG^16^. The correlation between VCDR and spherical equivalent is low (Supplementary Table 6), and adjusting for spherical equivalent did not lead to any major changes in the effects for these or other loci in European populations (Supplementary Table 7a), suggesting a joint genetic aetiology for POAG and myopia. In Asian cohorts, the direction of effect on VCDR at the chromosome 11 locus (*MIR612-SSSCA1* region) was not consistent with the European populations (Supplementary Table 7b). However, after adjusting for spherical equivalent the direction of effect on VCDR was similar to both populations. At the *BMP2* myopia locus, we observed a large difference in allele frequency between those of European and Asian ancestry (Table 1), which may explain the difference in effect direction.

### Risk for POAG

The 18 loci, together with age and sex, explain 5.1–5.9% of the VCDR phenotypic variability in Europeans (measured in the Rotterdam Study I, II and III), of which 1.6–1.8% is explained by the new loci. The phenotypic variability explained by all common SNPs is 41–53% in these cohorts, which is in line with the heritability estimates from family-based studies. In addition to confirming the previously published *CDKN2BAS* and *SIX1/6* POAG risk loci, we found nominally significant (*P*<0.05) associations with POAG for six newly identified genetic variants (*P*=8.1 × 10^−5^ from binomial test for chance of seeing six or more such nominally significant associations in 16 tests) (Supplementary Table 8), with odds ratios varying between 0.73 and 1.20. In the combined case-control studies, we found that the sum of all effects of these genes increased the risk of POAG 2.5-fold (Supplementary Table 9) for those in the highest quintile compared with those in the lowest quintile.

### Gene-based test

To identify new loci not previously found through individual SNP-based tests, we performed gene-based tests using VEGAS software^17^. Because of the smaller number of tests (17,872 genes tested), our gene-based significance threshold is *P*_gene-based_<0.05/17,872=2.80 × 10^−6^. In addition to the SNPs identified as significant (*P*<5 × 10^−8^) in a SNP-based test, we also found two new genes significantly associated with VCDR using the VEGAS gene-based test (Supplementary Table 10). These were *REEP5* (*P*=7.48 × 10^−7^) and *PITPNB* (*P*=4.89 × 10^−7^). *PITPNB* is ~800 kb from another gene with a significant SNP association (*CHEK2*, rs1547014) (Supplementary Fig. 7). Although the association signal centred over *CHEK2* extends a long distance towards *PITPNB*, a separate association peak over *PITPNB* can be observed, which is unrelated (no linkage disequilibrium (LD)) to the *CHEK2* peak. The results we obtained using the specified definition of the gene unit were substantially the same when alternative cutoff points from the transcription initiation and end sites were used (Supplementary Table 11). The *REEP5* gene showed no association with POAG (Supplementary Table 12). The *PITPNB* gene showed evidence for association with POAG in Australian & New Zealand Registry of Advanced Glaucoma (ANZRAG) (*P*=0.03) in the gene-based test, with a best single SNP *P* value of 0.003, but this was not confirmed in two other studies.

### Pathway analysis

To test whether gene-based statistics identified were enriched in 4,628 pre-specified Gene Ontology pathways, we performed pathway analysis using Pathway-VEGAS^18^. We used a pathway-wide significance threshold to be 1.08 × 10^−5^ (0.05/4,628). The only pathway exceeding the pathway-wide significance level was ‘negative regulation of cyclin-dependent protein kinase activity’ (Supplementary Table 13). The second top-pathway ‘negative regulation of epithelial cell proliferation’ is related to the top pathway, both suggesting retardation of cell growth. The ‘negative regulation of cyclin-dependent protein kinase activity’ finding was driven not only by the result at the *CDKN2A* locus but also by the result at *APC*, a gene close to *REEP5*.

### Regulatory elements and expression data

Six of the 18 most associated SNPs are located in DNase I hypersensitivity sites (Supplementary Table 14). The retinal pigment epithelium has the highest signal of all 125 available cell lines in one of these DNase I hypersensitivity sites. Thus, these results are suggesting that some of the SNPs may have their effect on VCDR by altering regulatory functions. We investigated the expression of the genes implicated in VCDR by these analyses in human ocular gene expression databases or the published literature. Most of these genes are expressed in eye tissues, including the optic nerve (Supplementary Tables 15 and 16).

## Discussion

This study reports 10 novel loci associated with VCDR, with an additional two loci identified using gene-based testing. Pathway analysis suggests retardation of cell growth as a major biological mechanism. The results for the most associated pathways ‘negative regulation of cyclin-dependent protein kinase activity’ and ‘negative regulation of epithelial cell proliferation’ are primarily driven by the *CDKN2A* and *CDKN2B* genes, respectively, but in both pathways the gene-based result at *APC* (*P*=7.20 × 10^−5^ in Caucasians and *P*=8.80 × 10^−3^ in Asians) also contributes to the pathway result. The *APC* gene has previously been reported to be a critical gene regulating retinal pigment epithelium proliferation and development^19^. These results add to our earlier findings on the role of growth and the transforming growth factor beta (TGFB) pathways in VCDR^10^. Various new genes fall into these pathways. The protein encoded by the *BMP2* (bone morphogenetic protein 2) gene on chromosome 20 belongs to the TGFB super-family. Two other new genes regulate apoptosis: *RPAP3* (RNA polymerase II-associated protein 3) on chromosome 12^20^ and *CARD10*, a gene that was previously found to be associated with disc area^13^. Another new VCDR association previously associated with disc area is *SALL1*^10^. This gene is implicated in ocular development.

Our findings offer new insights in the aetiology of optic nerve degeneration. *COL8A1* (collagen, type VIII, alpha 1) is part of a collagen pathway recently implicated in corneal thickness^18^, an ocular trait also associated with glaucoma risk. Missense mutations in *COL8A2* (collagen, type VIII, alpha2) were found in POAG patients with a very thin central corneal thickness (CCT)^21^. The collagen SNP (rs2623325) was not significantly associated with CCT (in Caucasians: *β*=−0.044, *P*=0.19; in Asians: *β*=0.007, *P*=0.89) or intraocular pressure (in Caucasians and Asians combined: *β*=−0.02, *P*=0.73) in largely the same cohorts^18^,^22^, suggesting that the collagen involvement in VCDR is not due to the influence by CCT or intraocular pressure. We also found several genes involved in cellular stress response. *DUSP1* (dual specificity phosphatase 1) is the nearest gene to the most strongly associated SNP on chromosome 5. This gene, inducible by oxidative stress and heat shock, may play a role in environmental stress response^23^, and may also participate in the negative regulation of cellular proliferation. *HSF2* (heat shock transcription factor 2), one of the genes at the chromosome 6 locus, also is part of the cellular stress response pathway. Deficiency of this factor causes various central nervous system defects in mice^24^,^25^. Another pathway emerging in this study is that of exocytosis. The SNP on the other chromosome 6 locus is located in *EXOC2* (exocyst complex component 2). The encoded protein is one of the eight proteins of the exocyst complex^26^. This multi-protein complex is important for directing exocytic vesicles to the plasma membrane, a mechanism that also has been implicated in neuronal degeneration in the brain^27^. Lipid metabolism emerges as another pathway. The gene on chromosome 10, *PLCE1* (phospholipase C, epsilon 1), belongs to the phospholipase C family, which plays a role in the generation of second messengers^28^. Various processes affecting cell growth, differentiation and gene expression are regulated by these second messengers. From a clinical perspective, the findings on *ADAMTS8* are of interest. ADAMTS enzymes have different functions, including the formation and turnover of the extracellular matrix^29^. Strikingly, a variant in *ADAMTS10* has been linked to a form of glaucoma in dogs^30^,^31^.

In summary, we have now identified 10 novel loci associated with cupping of the optic nerve, a key determinant of glaucoma. Together, these genetic risk variants increased the risk of POAG in case-control validation studies. Pathway analysis implicated negative regulation of cell growth and cellular response to environmental stress as key pathological pathways in glaucoma, and that novel therapies targeting these pathways may be neuro-protective in glaucoma.

## Methods

### Study design

We performed a meta-analysis on directly genotyped and imputed SNPs from individuals of European ancestry in 10 studies, with a total of 21,094 individuals. Subsequently, we evaluated significantly associated SNPs in 6,784 subjects of Asian origin including four different studies and performed a meta-analysis on all studies combined.

### Subjects and phenotyping

All studies included in this meta-analysis are part of the International Glaucoma Genetics Consortium. The ophthalmological examination of each study included an assessment of the optic nerve head to measure the VCDR (Supplementary Table 17a). Unreliable optic nerve data were excluded.

The meta-analysis of stage 1 was based on 10 studies of European ancestry: Brisbane Adolescent Twin Study, Blue Mountains Eye Study, Erasmus Rucphen Family Study, Gutenberg Health Study (GHS I/GHS II), Glaucoma Genes and Environment (controls only), National Eye Institute Glaucoma Human Genetics Collaboration (NEIGHBOR; controls only), Raine Study, Rotterdam Study (RS-I/RS-II/RS-III), Twins Eye Study in Tasmania and TwinsUK. Stage 2 comprised four Asian studies: Beijing Eye Study, Singapore Chinese Eye Study, Singapore Malay Eye Study and Singapore Indian Eye Study. For each SNP with the strongest association at each locus the association with POAG was tested in five case-control studies: ANZRAG, deCODE, Massachusetts Eye and Ear Infirmary, NEIGHBOR and Southampton.

Information on general methods, demographics, phenotyping and genotyping methods of the study cohorts can be found in Supplementary Tables 1 and 17 and the Supplementary Note. All studies were performed with the approval of their local medical ethics committee, and written informed consent was obtained from all participants in accordance with the Declaration of Helsinki.

### Genotyping and imputation

Information on genotyping in each cohort and the particular platforms used to perform genotyping can be found in more detail in Supplementary Table 17b. To produce consistent data sets and enable a meta-analysis of studies across different genotyping platforms, the studies performed genomic imputation on available HapMap Phase 2 genotypes with MACH^32^ or IMPUTE^33^, using the appropriate ancestry groups as templates.

Each study applied stringent quality control procedures before imputation, including minor allele frequency cutoffs, Hardy–Weinberg equilibrium, genotypic success rate, mendelian inconsistencies, exclusion of individuals with >5% shared ancestry (exception made for family-based cohorts in which due adjustment for family relationship was made) and removal of all individuals whose ancestry as determined through genetic analysis did not match the prevailing ancestry group of the corresponding cohort (Supplementary Note). SNPs with low imputation quality were filtered using metrics specific to the imputation method and thresholds used in previous GWAS analyses. For each cohort, only SNPs with imputation quality scores >0.6 (proper-info of IMPUTE) or R2>0.6 (MACH) were included into the meta-analysis.

### Statistical analysis

In subjects drawn from their respective populations in which the prevalence of glaucomatous changes is relatively low, the correlation between left and right eye is high^34^. Therefore, we used the mean VCDR of both eyes. In cases of missing or unreliable data for one eye, data of the other eye was taken. Each individual study did a linear regression model between the VCDR and the SNPs under the assumption of an additive model for the effect of the risk allele. Analyses were adjusted for age, sex and the first two principal components (for population-based studies) or family structure (for family-based studies). Secondary analyses were done with adjustments for disc area or spherical equivalent. In the Rotterdam Studies, we calculated the phenotypic variability explained by the new loci, and explained by all common SNPs using the ‘Genome-wide Complex Trait Analysis’ tool^35^,^36^.

We performed an inverse variance weighted fixed-effect meta-analysis. This was performed with METAL software^37^. *P* values for the association results were calculated by using the *z*-statistic. *P* values for heterogeneity were calculated by using the Cochran’s *Q*-test for heterogeneity. In addition to this, *I*^2^ values were calculated to assess heterogeneity^38^. *F*_st_ values were calculated to assess the genetic variation due to subdivision of populations. All study effect estimates were corrected using genomic control and were oriented to the positive strand of the NCBI Build 36 reference sequence of the human genome, which was the genomic build on which most available genotyping platforms were based. Coordinates and further annotations for the SNPs were converted into Build 37, the most recent version of the available builds at the time of this study.

In stage 1, a *P* value <5.0 × 10^−8^ (the genome-wide threshold of association) was considered significant. In stage 2, a *P* value <0.05 was considered significant. Manhattan, regional and forest plots were made using *R*^39^, LocusZoom^40^ and Stata/SE 12.0 (StataCorp LP, College Station, TX, USA).

### Risk-score models

In five case-control studies, a weighted genetic risk score per individual was calculated. Standardized regression coefficients were used as weighting factor. The weighted risk scores were divided into quintiles. Odds ratios were calculated for each quintile, using the first quintile as a reference.

### Gene-based test using VEGAS

There are different gene-based tests of which VEGAS is one of the most powerful tests^41^. We therefore performed gene-based testing using VEGAS software^17^, which combines the test statistics of all SNPs present within and 50 kb upstream/downstream of each gene. LD between the markers is accounted for through simulations from the multivariate normal distribution, based on estimates of LD from reference populations. Since Asian and European ancestry populations show different LD patterns, we performed separate gene-based tests for each population. Hapmap 2 CEU population was used as a reference to calculate LD for European ancestry data, whereas Hapmap 2 JPT and CHB combined population was used as a reference for Asian ancestry data. After calculation of gene-based test statistics for Asian and European ancestry populations separately, meta-analysis was conducted using Fisher’s method for combining *P* values. VEGAS was applied to the summary data from the full VCDR analysis (as in Table 1) and to three of the POAG data sets; ANZRAG, Massachusetts Eye and Ear Infirmary glaucoma clinic and Glaucoma Genes and Environment (Supplementary Note).

### Pathway-analysis using pathway-VEGAS

Pre-specified pathways from the Gene Ontology database with size ranging in 5–500 genes were used to perform pathway analysis. Pathway-VEGAS combines VEGAS gene-based test statistics based on pre-specified biological pathways^18^. Pathway *P* values were computed by summing *χ*^2^-test statistics derived from VEGAS *P* values. Empirical ‘VEGAS-pathway’ *P* values for each pathway were computed by comparing the real-data-summed *χ*^2^-test statistics with 500,000 simulations where the relevant number (as per size of pathway) of randomly drawn *χ*^2^-test statistics was summed. To ensure clusters of genes did not adversely affect results, within each pathway, gene sets were pruned such that each gene was >500 kb from all other genes in the pathway. Where required, all but one of the clustered genes was dropped at random when genes were clustered. Pathway-VEGAS was performed separately for European and Asian ancestry data sets. Meta-analysis was conducted using Fisher’s method for combining *P* values.

### Regulatory functions

We used the ENCyclopedia Of DNA Elements^42^ data in the UCSC Genome Browser^43^ to look at DNase I hypersensitivity sites and other functional elements.

### Gene expression in human eye tissue

We examined the expression of genes that reached significance in the individual SNP-based test or gene-based test. We used published literature or human ocular gene expression databases (Supplementary Tables 15 and 16).

## Author contributions

H.S., R.H., A.Mishra, P.G.H., C.-C.K. and S.J.L. contributed equally to this work. N.P., T.-Y.W., L.R.P., A.W.H., C.M.v.D. and C.J.H. jointly supervised this work. H.S., R.H., P.G.H., T.-Y.W., L.R.P., A.W.H., C.M.v.D. and C.J.H. performed analyses and drafted the manuscript. J.B.J., A.C.V., C.C.W.K., J.E.C., S.M., D.A.M., A.J.L., J.L.W., N.P., T.-Y.W., L.R.P., A.W.H., C.M.v.D. and C.J.H. jointly conceived the project and supervised the work. W.D.R., E.V., M.E.N., G.W.M., L.X., J.E.M., Y.L., N.A., L.C.K., K.-S.S., E.M.v.L., A.I.I., V.J.M.V., M.A.H., S.-C.L., D.D.G.D., A.N., C.V., P.G.S., A.S., J.H.K., J.L., F.J., A.J.C., L.M.E.v.K., F.R., E.S., V.J., G.M., R.N.W., P.T.V.M.d.J., B.A.O., A.G.U., A.H., S.E., T.D.S., A.Mirshahi, S.-M.S., J.R.V., Y.-Y.T., R.C.W.W., H.G.L., E.-S.T., N.M.J., C.-Y.C., T.A., Blue Mountains Eye Study-GWAS Group, NEIGHBORHOOD Consortium, and Wellcome Trust Case Control Consortium 2 (WTCCC 2) were responsible for study-specific data. H.S., S.J.L., J.N.C.B., J.G., G.T., P.G., U.T., K.P.B., J.L.H., J.E.C., A.J.L., K.S. and J.L.W. were involved in the genetic risk-score analysis. S.F.J., X.L., A.A.B.B. and T.L.Y. performed the data expression experiments. A.Mishra and S.M. were involved in pathway analyses. A.Mishra, C.-C.K., W.D.R., P.T.V.M.d.J., H.G.L., N.M.J., J.B.J., A.C.V., C.C.W.K., J.E.C., S.M., D.A.M., A.J.L. and J.L.W. critically reviewed the manuscript.

## Additional information

**How to cite this article:** Springelkamp, H. *et al*. Meta-analysis of genome-wide association studies identifies novel loci that influence cupping and the glaucomatous process. *Nat. Commun.* 5:4883 doi: 10.1038/ncomms5883 (2014).

## Supplementary Material

Supplementary Information Supplementary Figures 1-7, Supplementary Tables 1-17, Supplementary Note, Supplementary Methods and Supplementary References

## Figures and Tables

**Table 1:** Summary of the results of the meta-analyses of genome-wide association studies.

## References

[b1] Quigley H. A. & Broman A. T. The number of people with glaucoma worldwide in 2010 and 2020. Br. J. Ophthalmol. 90, 262–267 (2006). 16488940 10.1136/bjo.2005.081224 PMC1856963

[b2] Chang T. C. . Determinants and heritability of intraocular pressure and cup-to-disc ratio in a defined older population. Ophthalmology 112, 1186–1191 (2005). 15939473 10.1016/j.ophtha.2005.03.006 PMC3124001

[b3] Charlesworth J. . The path to open-angle glaucoma gene discovery: endophenotypic status of intraocular pressure, cup-to-disc ratio, and central corneal thickness. Invest. Ophthalmol. Vis. Sci. 51, 3509–3514 (2010). 20237253 10.1167/iovs.09-4786 PMC2904007

[b4] Coleman A. L. Glaucoma. Lancet 354, 1803–1810 (1999). 10577657 10.1016/S0140-6736(99)04240-3

[b5] Klein B. E., Klein R. & Lee K. E. Heritability of risk factors for primary open-angle glaucoma: the Beaver Dam Eye Study. Invest. Ophthalmol. Vis. Sci. 45, 59–62 (2004). 14691154 10.1167/iovs.03-0516

[b6] van Koolwijk L. M. . Major genetic effects in glaucoma: commingling analysis of optic disc parameters in an older Australian population. Invest. Ophthalmol. Vis. Sci. 50, 5275–5280 (2009). 19458335 10.1167/iovs.08-3065

[b7] Sanfilippo P. G., Hewitt A. W., Hammond C. J. & Mackey D. A. The heritability of ocular traits. Surv. Ophthalmol. 55, 561–583 (2010). 20851442 10.1016/j.survophthal.2010.07.003

[b8] Burdon K. P. . Genome-wide association study identifies susceptibility loci for open angle glaucoma at TMCO1. and CDKN2B-AS1. Nat. Genet. 43, 574–578 (2011). 10.1038/ng.824 21532571

[b9] Macgregor S. . Genome-wide association identifies ATOH7 as a major gene determining human optic disc size. Hum. Mol. Genet. 19, 2716–2724 (2010). 20395239 10.1093/hmg/ddq144 PMC2883339

[b10] Ramdas W. D. . A genome-wide association study of optic disc parameters. PLoS Genet. 6, e1000978 (2010). 20548946 10.1371/journal.pgen.1000978 PMC2883590

[b11] Ramdas W. D. . Common genetic variants associated with open-angle glaucoma. Hum. Mol. Genet. 20, 2464–2471 (2011). 21427129 10.1093/hmg/ddr120

[b12] Ramdas W. D. . Clinical implications of old and new genes for open-angle glaucoma. Ophthalmology 118, 2389–2397 (2011). 21872936 10.1016/j.ophtha.2011.05.040

[b13] Khor C. C. . Genome-wide association studies in Asians confirm the involvement of ATOH7 and TGFBR3, and further identify CARD10 as a novel locus influencing optic disc area. Hum. Mol. Genet. 20, 1864–1872 (2011). 21307088 10.1093/hmg/ddr060

[b14] Ramdas W. D. . Heidelberg Retina Tomograph (HRT3) in population-based epidemiology: normative values and criteria for glaucomatous optic neuropathy. Ophthalmic Epidemiol. 18, 198–210 (2011). 21961509 10.3109/09286586.2011.602504

[b15] Verhoeven V. J. . Genome-wide meta-analyses of multiancestry cohorts identify multiple new susceptibility loci for refractive error and myopia. Nat. Genet. 45, 314–318 (2013). 23396134 10.1038/ng.2554 PMC3740568

[b16] Kwon Y. H., Fingert J. H., Kuehn M. H. & Alward W. L. Primary open-angle glaucoma. N. Engl. J. Med. 360, 1113–1124 (2009). 19279343 10.1056/NEJMra0804630 PMC3700399

[b17] Liu J. Z. . A versatile gene-based test for genome-wide association studies. Am. J. Hum. Genet. 87, 139–145 (2010). 20598278 10.1016/j.ajhg.2010.06.009 PMC2896770

[b18] Lu Y. . Genome-wide association analyses identify multiple loci associated with central corneal thickness and keratoconus. Nat. Genet. 45, 155–163 (2013). 23291589 10.1038/ng.2506 PMC3720123

[b19] Marcus D. M. . Retinal pigment epithelium abnormalities in mice with adenomatous polyposis coli gene disruption. Arch. Ophthalmol. 115, 645–650 (1997). 9152133 10.1001/archopht.1997.01100150647013

[b20] Ni L. . RPAP3 interacts with Reptin to regulate UV-induced phosphorylation of H2AX and DNA damage. J. Cell. Biochem. 106, 920–928 (2009). 19180575 10.1002/jcb.22073

[b21] Desronvil T. . Distribution of COL8A2 and COL8A1 gene variants in Caucasian primary open angle glaucoma patients with thin central corneal thickness. Mol. Vis. 16, 2185–2191 (2010). 21139683 PMC2994337

[b22] Hysi P. G. . Genome-wide analysis of multi-ancestry cohorts identifies new loci influencing intraocular pressure and susceptibility to glaucoma. Nat. Genet. 10.1038/ng.3087 (2014). PMC4177225 25173106

[b23] Keyse S. M. & Emslie E. A. Oxidative stress and heat shock induce a human gene encoding a protein-tyrosine phosphatase. Nature 359, 644–647 (1992). 1406996 10.1038/359644a0

[b24] Kallio M. . Brain abnormalities, defective meiotic chromosome synapsis and female subfertility in HSF2 null mice. EMBO J. 21, 2591–2601 (2002). 12032072 10.1093/emboj/21.11.2591 PMC125382

[b25] Wang G., Zhang J., Moskophidis D. & Mivechi N. F. Targeted disruption of the heat shock transcription factor (hsf)-2 gene results in increased embryonic lethality, neuronal defects, and reduced spermatogenesis. Genesis 36, 48–61 (2003). 12748967 10.1002/gene.10200

[b26] Lipschutz J. H. & Mostov K. E. Exocytosis: the many masters of the exocyst. Curr. Biol. 12, R212–R214 (2002). 11909549 10.1016/s0960-9822(02)00753-4

[b27] Coleman P. D. & Yao P. J. Synaptic slaughter in Alzheimer's disease. Neurobiol. Aging 24, 1023–1027 (2003). 14643374 10.1016/j.neurobiolaging.2003.09.001

[b28] Lopez I., Mak E. C., Ding J., Hamm H. E. & Lomasney J. W. A novel bifunctional phospholipase c that is regulated by Galpha 12 and stimulates the Ras/mitogen-activated protein kinase pathway. J. Biol. Chem. 276, 2758–2765 (2001). 11022047 10.1074/jbc.M008119200

[b29] Apte S. S. A disintegrin-like and metalloprotease (reprolysin type) with thrombospondin type 1 motifs: the ADAMTS family. Int. J. Biochem. Cell Biol. 36, 981–985 (2004). 15094112 10.1016/j.biocel.2004.01.014

[b30] Kuchtey J. . Screening ADAMTS10 in dog populations supports Gly661Arg as the glaucoma-causing variant in beagles. Invest. Ophthalmol. Vis. Sci. 54, 1881–1886 (2013). 23422823 10.1167/iovs.12-10796 PMC3604907

[b31] Kuchtey J. . Mapping of the disease locus and identification of ADAMTS10 as a candidate gene in a canine model of primary open angle glaucoma. PLoS Genet. 7, e1001306 (2011). 21379321 10.1371/journal.pgen.1001306 PMC3040645

[b32] Li Y., Willer C. J., Ding J., Scheet P. & Abecasis G. R. MaCH: using sequence and genotype data to estimate haplotypes and unobserved genotypes. Genet. Epidemiol. 34, 816–834 (2010). 21058334 10.1002/gepi.20533 PMC3175618

[b33] Marchini J., Howie B., Myers S., McVean G. & Donnelly P. A new multipoint method for genome-wide association studies by imputation of genotypes. Nat. Genet. 39, 906–913 (2007). 17572673 10.1038/ng2088

[b34] Li H., Healey P. R., Tariq Y. M., Teber E. & Mitchell P. Symmetry of optic nerve head parameters measured by the heidelberg retina tomograph 3 in healthy eyes: the Blue Mountains Eye study. Am. J. Ophthalmol. 155, 518–523 e1 (2013). 23218692 10.1016/j.ajo.2012.09.019

[b35] Yang J., Lee S. H., Goddard M. E. & Visscher P. M. GCTA: a tool for genome-wide complex trait analysis. Am. J. Hum. Genet. 88, 76–82 (2011). 21167468 10.1016/j.ajhg.2010.11.011 PMC3014363

[b36] Yang J. . Common SNPs explain a large proportion of the heritability for human height. Nat. Genet. 42, 565–569 (2010). 20562875 10.1038/ng.608 PMC3232052

[b37] Willer C. J., Li Y. & Abecasis G. R. METAL: fast and efficient meta-analysis of genomewide association scans. Bioinformatics 26, 2190–2191 (2010). 20616382 10.1093/bioinformatics/btq340 PMC2922887

[b38] Higgins J. P. & Thompson S. G. Quantifying heterogeneity in a meta-analysis. Stat. Med. 21, 1539–1558 (2002). 12111919 10.1002/sim.1186

[b39] R Core Team. R: a language and environment for statistical computing, http://www.R-project.org (2014).

[b40] Pruim R. J. . LocusZoom: regional visualization of genome-wide association scan results. Bioinformatics 26, 2336–2337 (2010). 20634204 10.1093/bioinformatics/btq419 PMC2935401

[b41] Li M. X., Gui H. S., Kwan J. S. & Sham P. C. GATES: a rapid and powerful gene-based association test using extended Simes procedure. Am. J. Hum. Genet. 88, 283–293 (2011). 21397060 10.1016/j.ajhg.2011.01.019 PMC3059433

[b42] Consortium E. P. A user's guide to the encyclopedia of DNA elements (ENCODE). PLoS Biol. 9, e1001046 (2011). 21526222 10.1371/journal.pbio.1001046 PMC3079585

[b43] Kent W. J. . The human genome browser at UCSC. Genome Res. 12, 996–1006 (2002). 12045153 10.1101/gr.229102 PMC186604

