# Supplementary material for: Meta-analysis of genome-wide association studies identifies novel loci that influence cupping and the glaucomatous process
Source: Nat Commun. 2014 Sep 22;5:4883. doi: 10.1038/ncomms5883 (PMC4199103; doi:10.1038/ncomms5883)
Supplement: Supplementary Information — Supplementary Figures 1-7, Supplementary Tables 1-17, Supplementary Note, Supplementary Methods and Supplementary References [file ncomms5883-s1.pdf]

Supplementary Figure 1: Histograms of the vertical cup-disc ratio (VCDR) distribution based on individuals which are included in the analysis

BATS

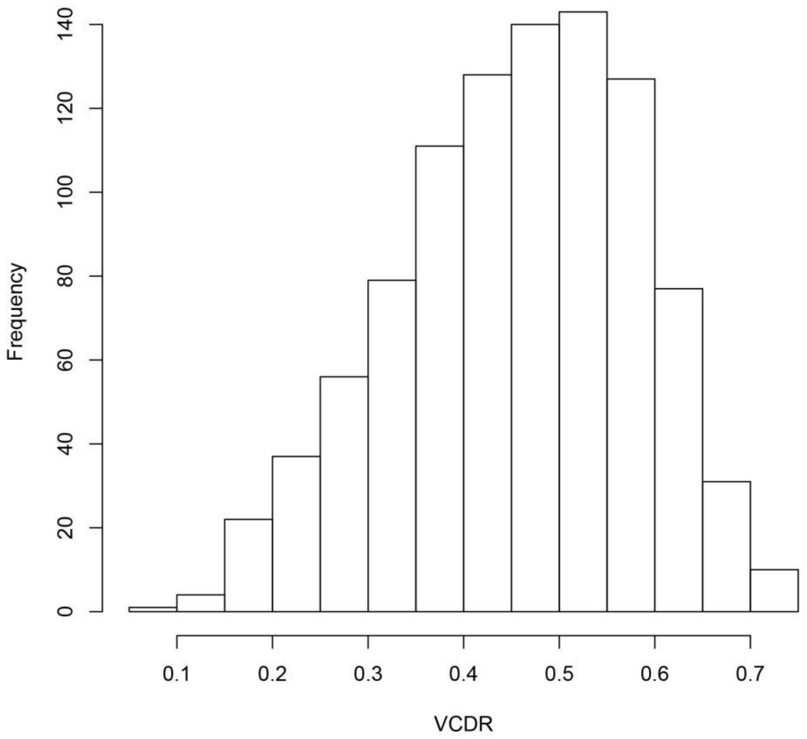

BMES

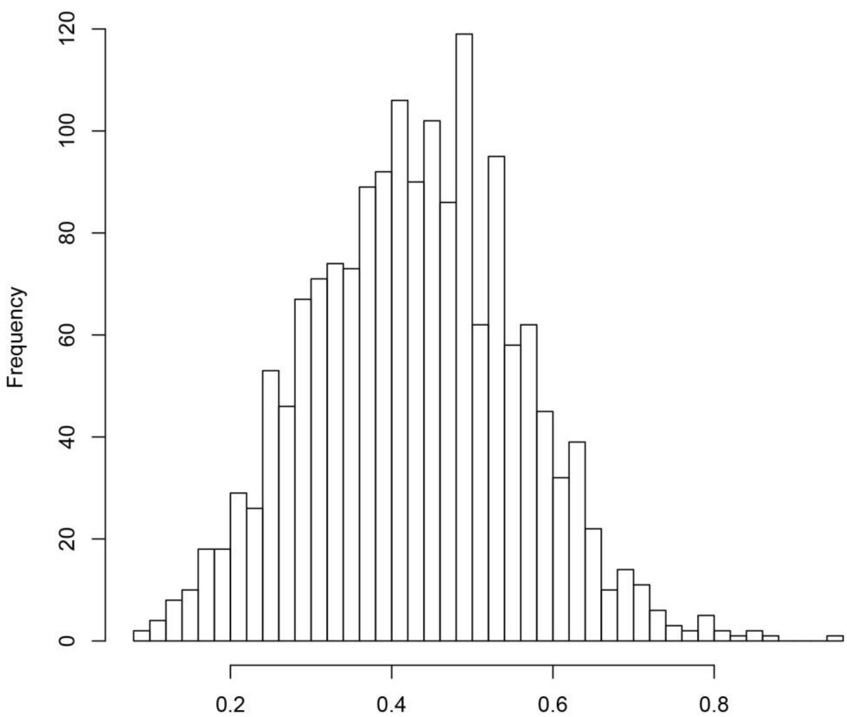

ERF

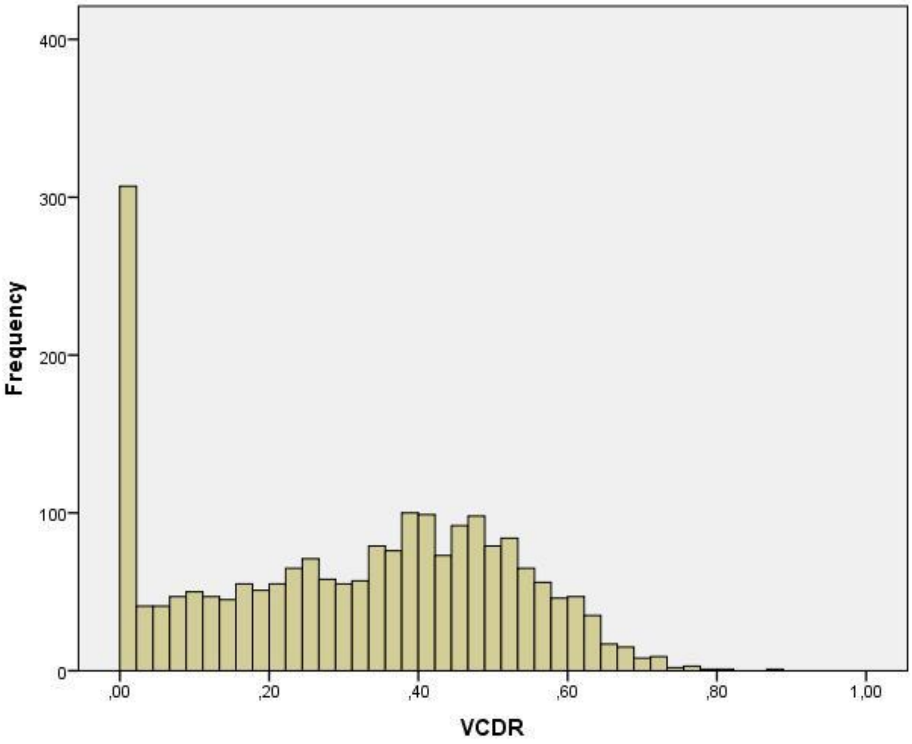

400
300
200
100
0
Frequency
,00
,20
,40
,60
,80
1,00
VCDR

GHS I

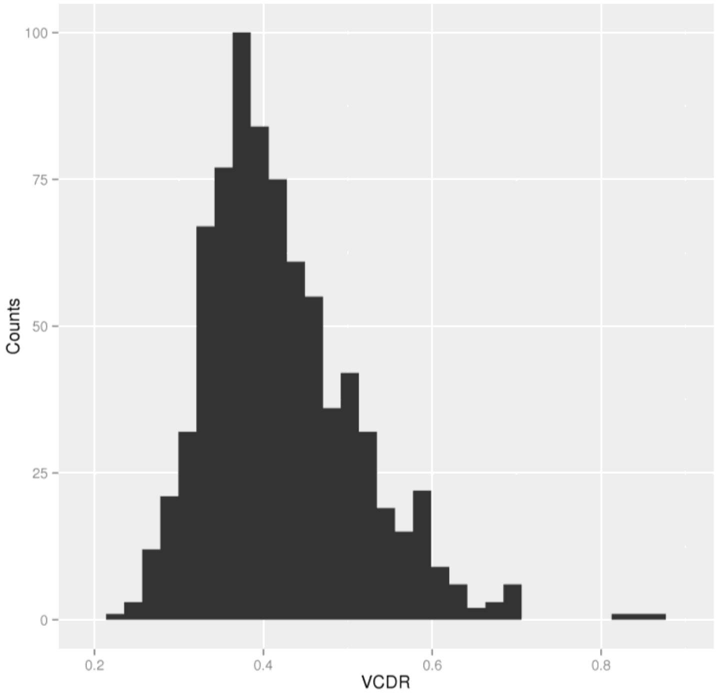

100
75
50
25
0
Counts
0.2
0.4
0.6
0.8
VCDR

GHS II

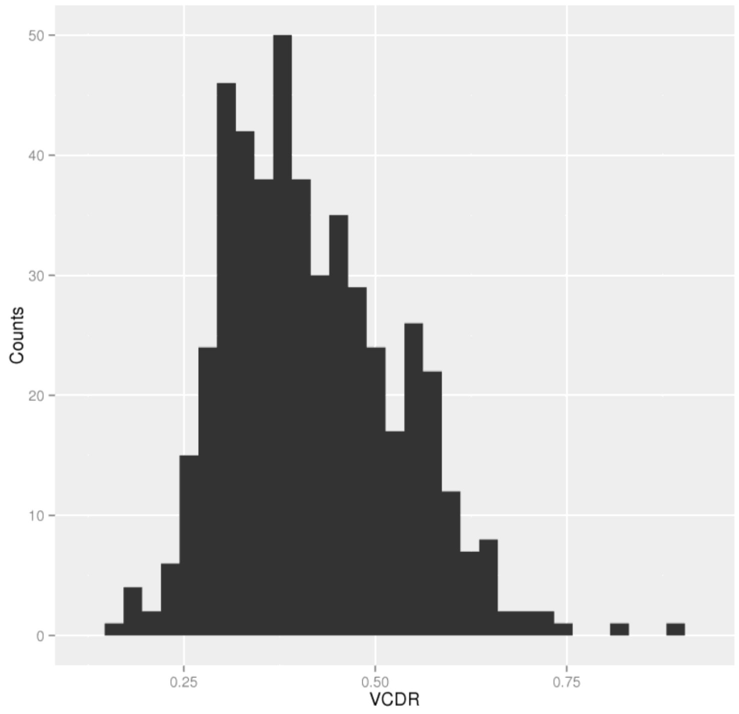

GLAUGEN

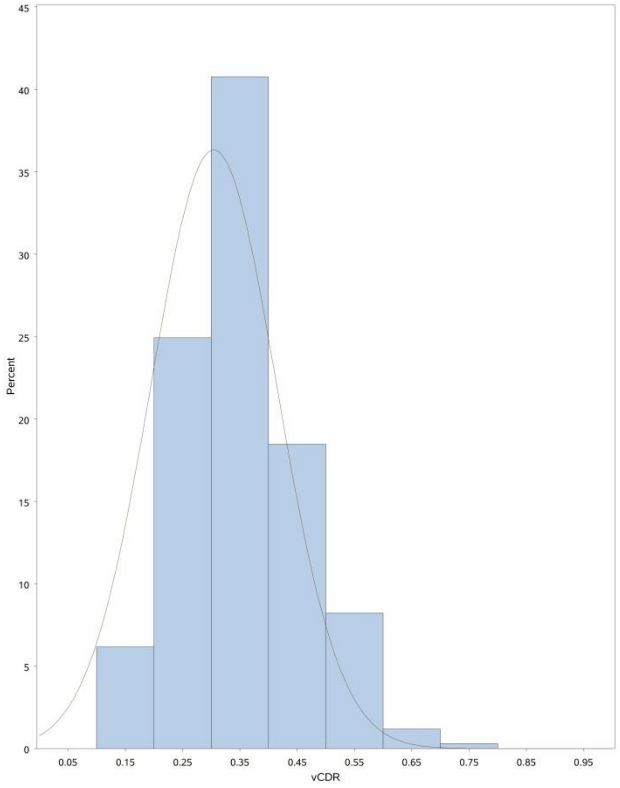

NEIGHBOR

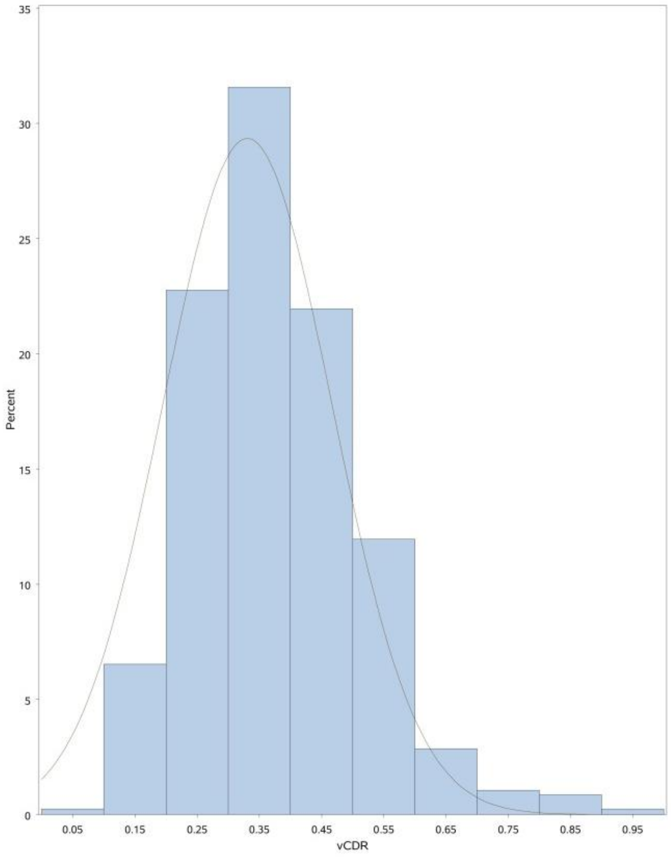

RAINE

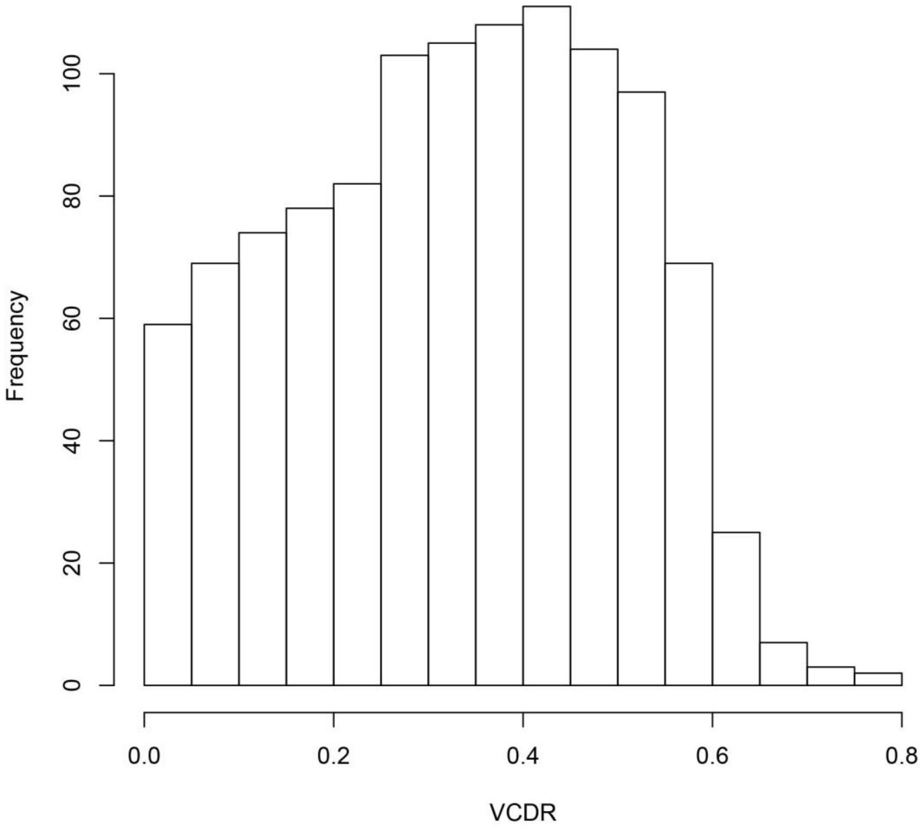

Rotterdam Study I

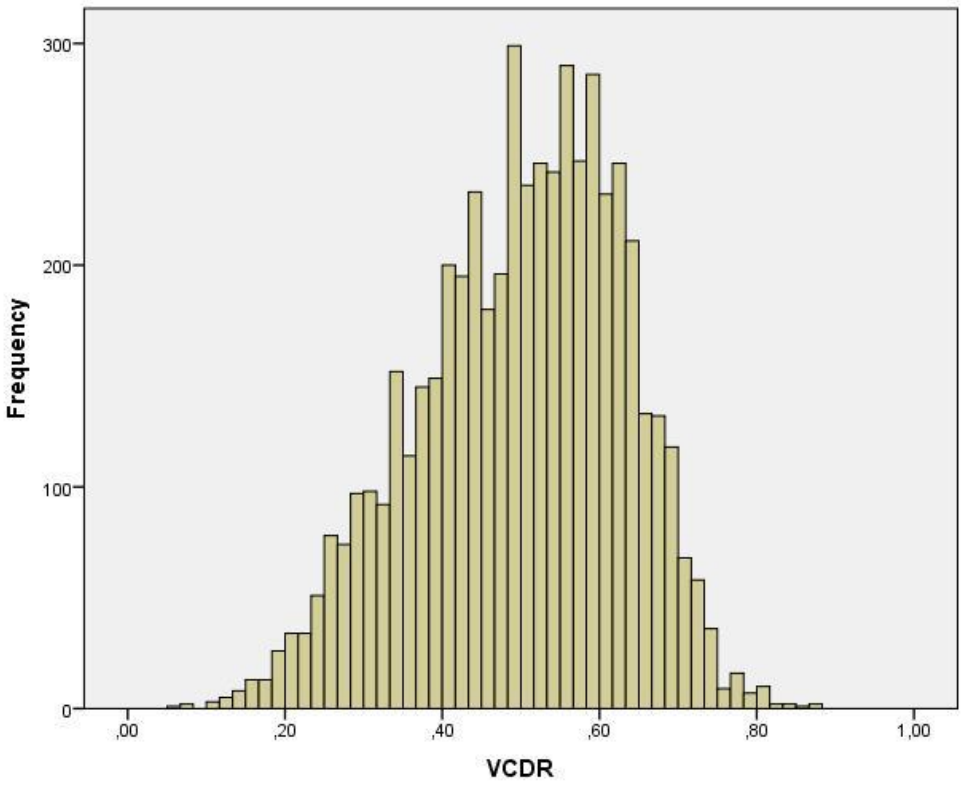

Rotterdam Study II

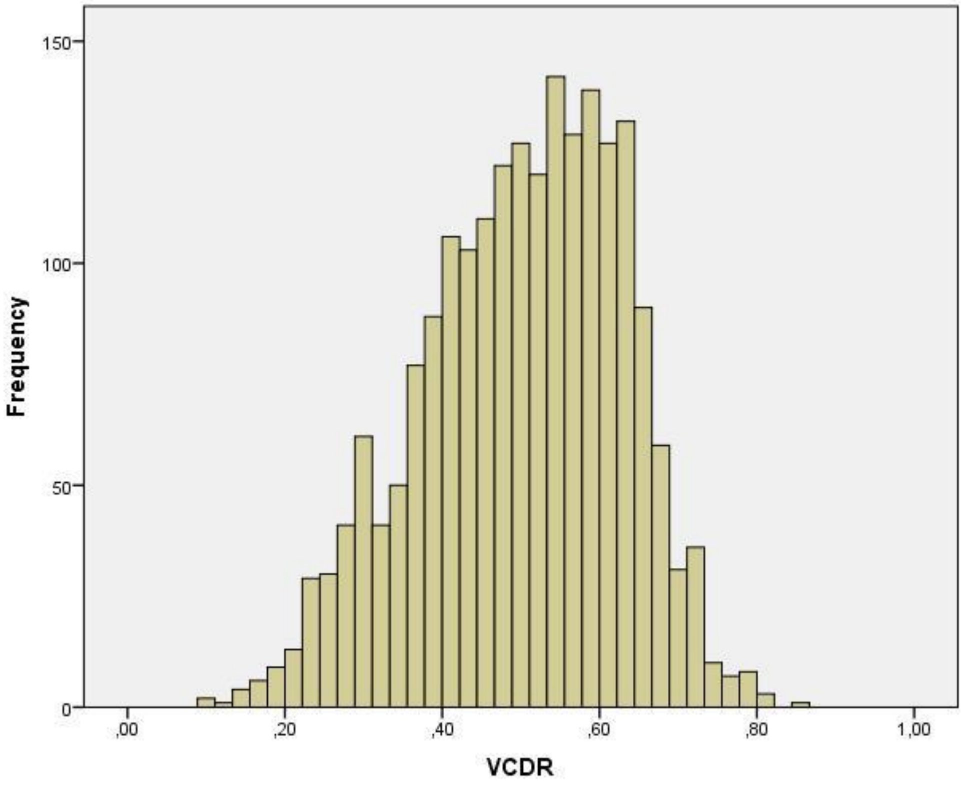

Rotterdam Study III

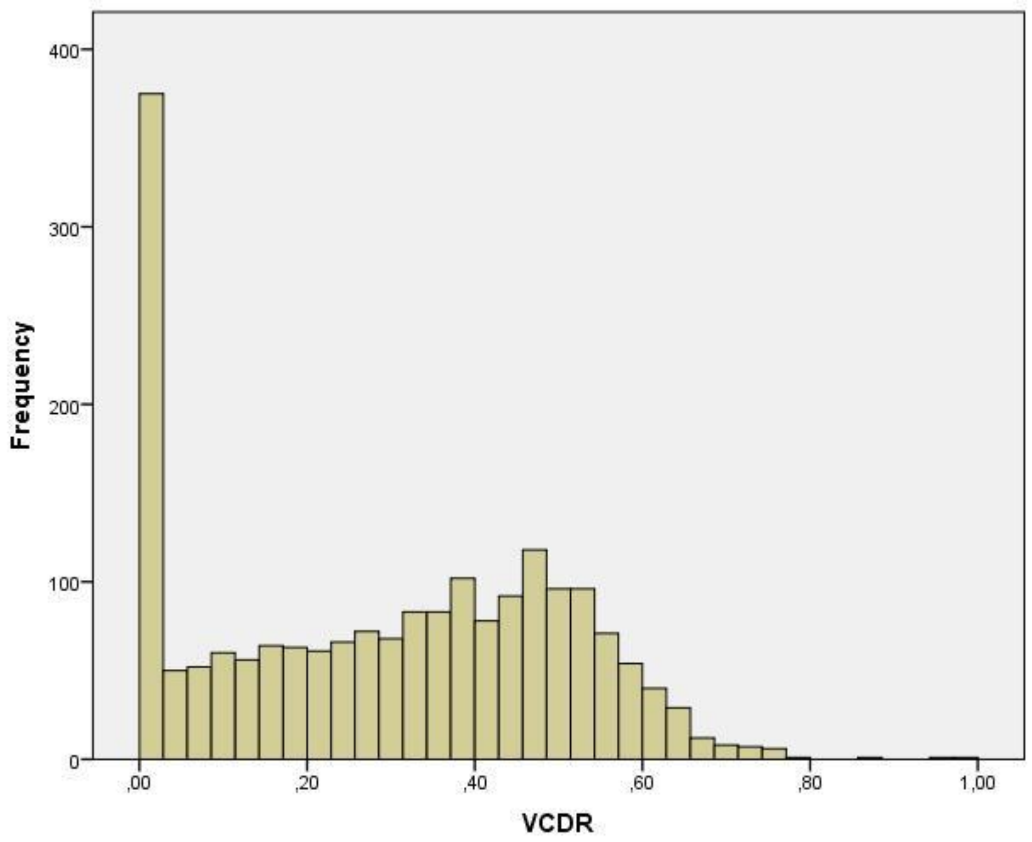

TEST

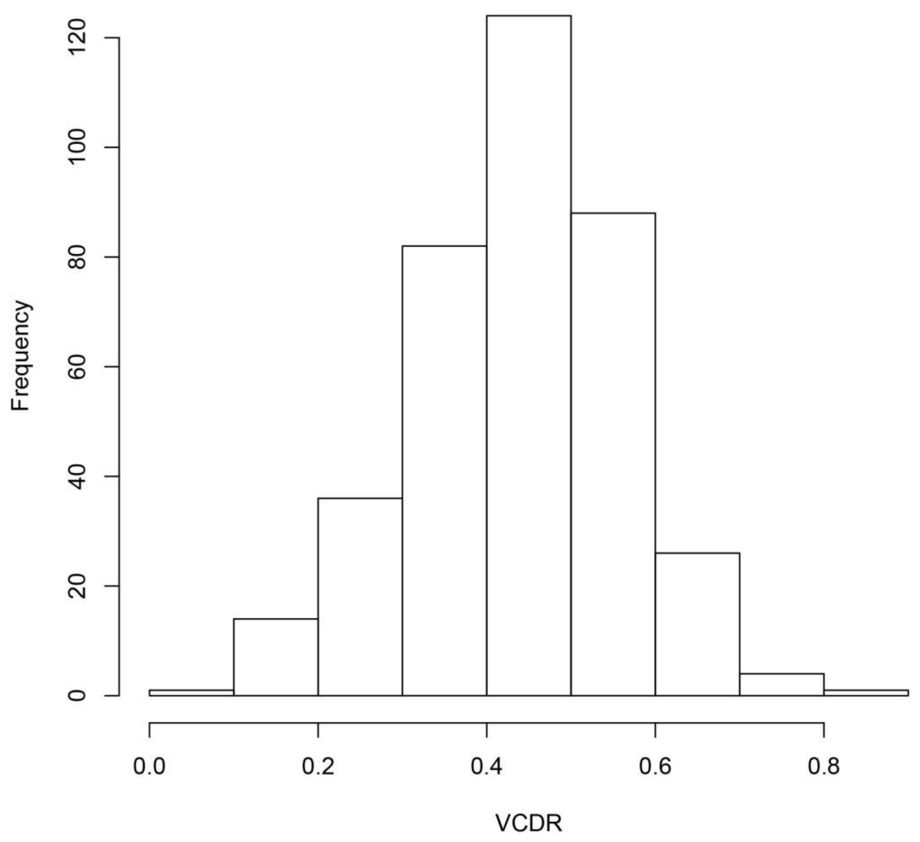

TwinsUK

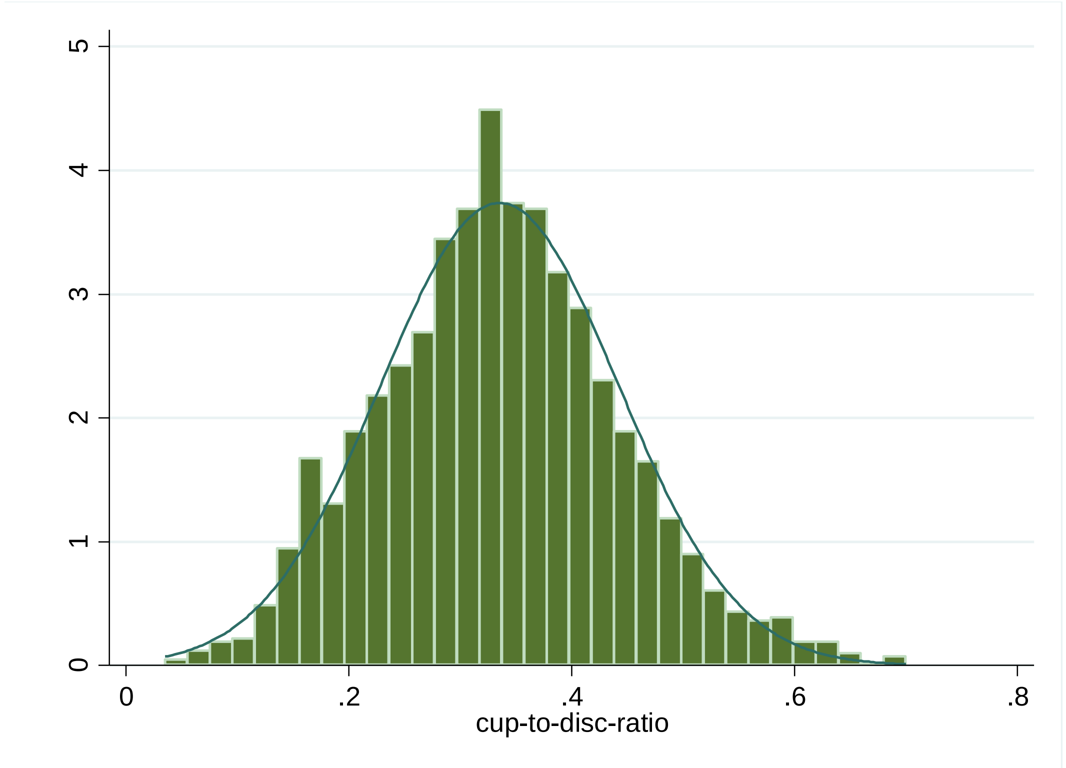

Beijing Eye Study

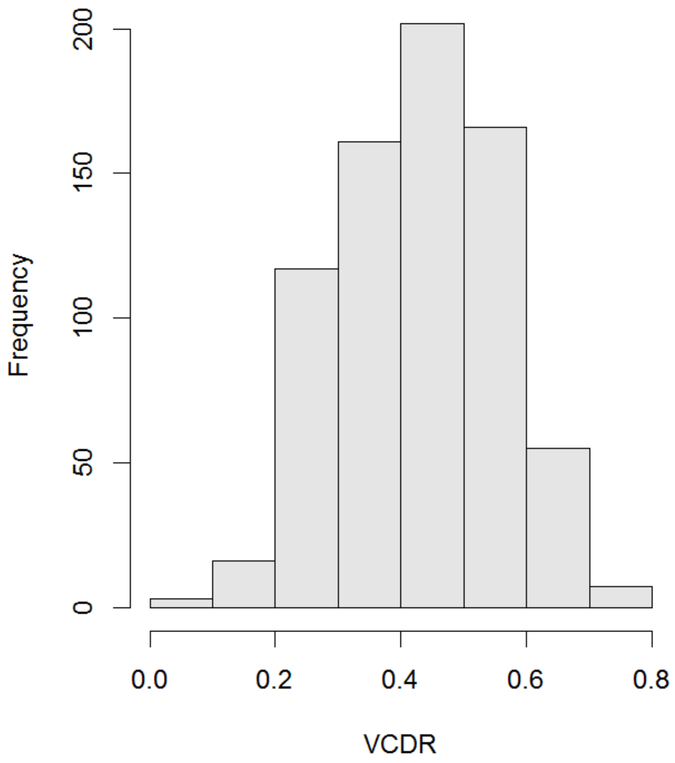

SCES

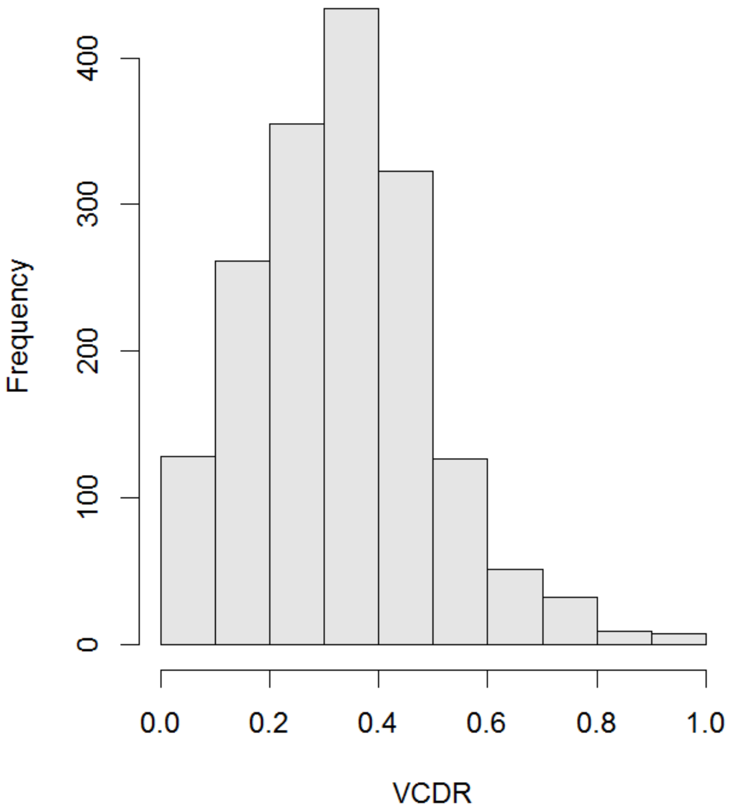
Frequency
0
100
200
300
400
0.0
0.2
0.4
0.6
0.8
1.0
VCDR

SIMES

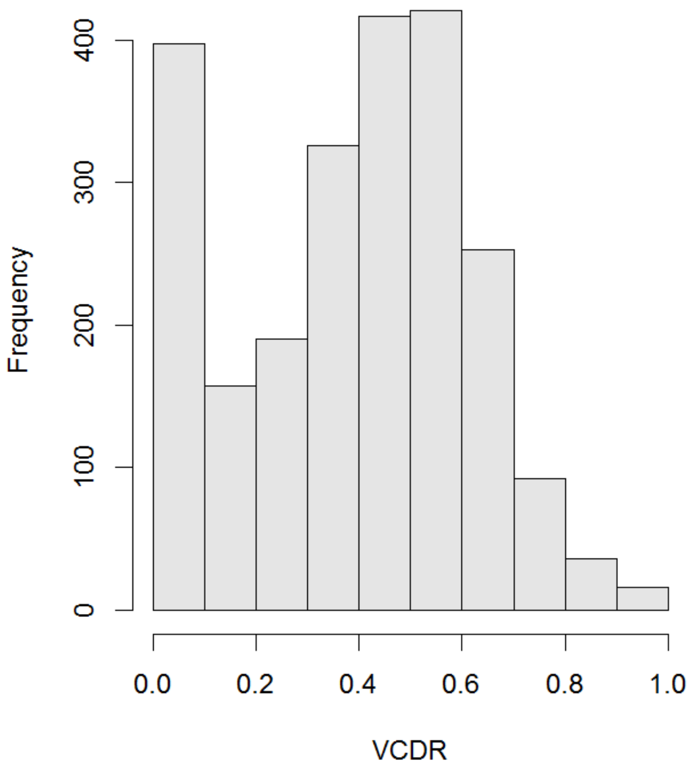
Frequency
0
100
200
300
400
0.0
0.2
0.4
0.6
0.8
1.0
VCDR

SINDI

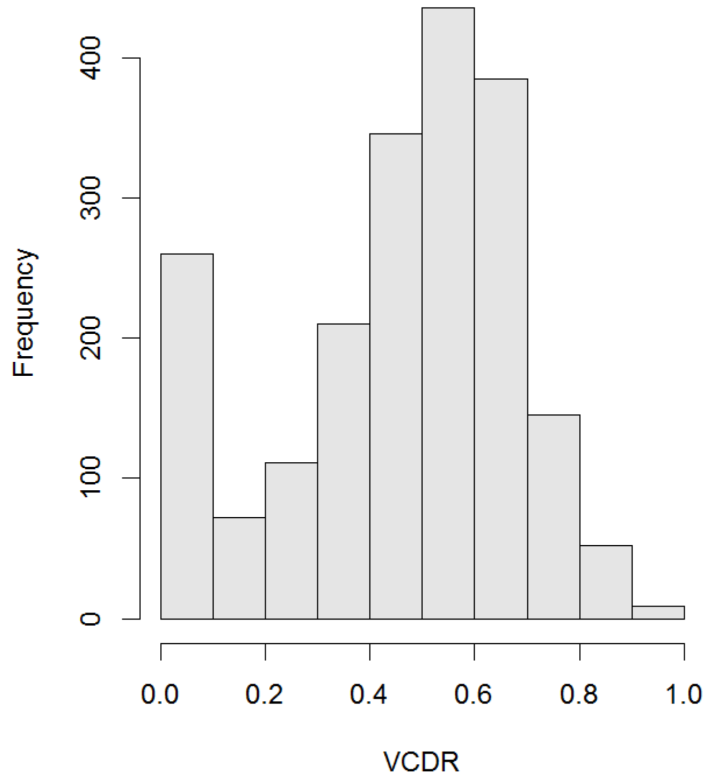

Supplementary Figure 2a. Quantile-quantile (QQ) plot of vertical cup-disc ratio (VCDR) in subjects of European ancestry

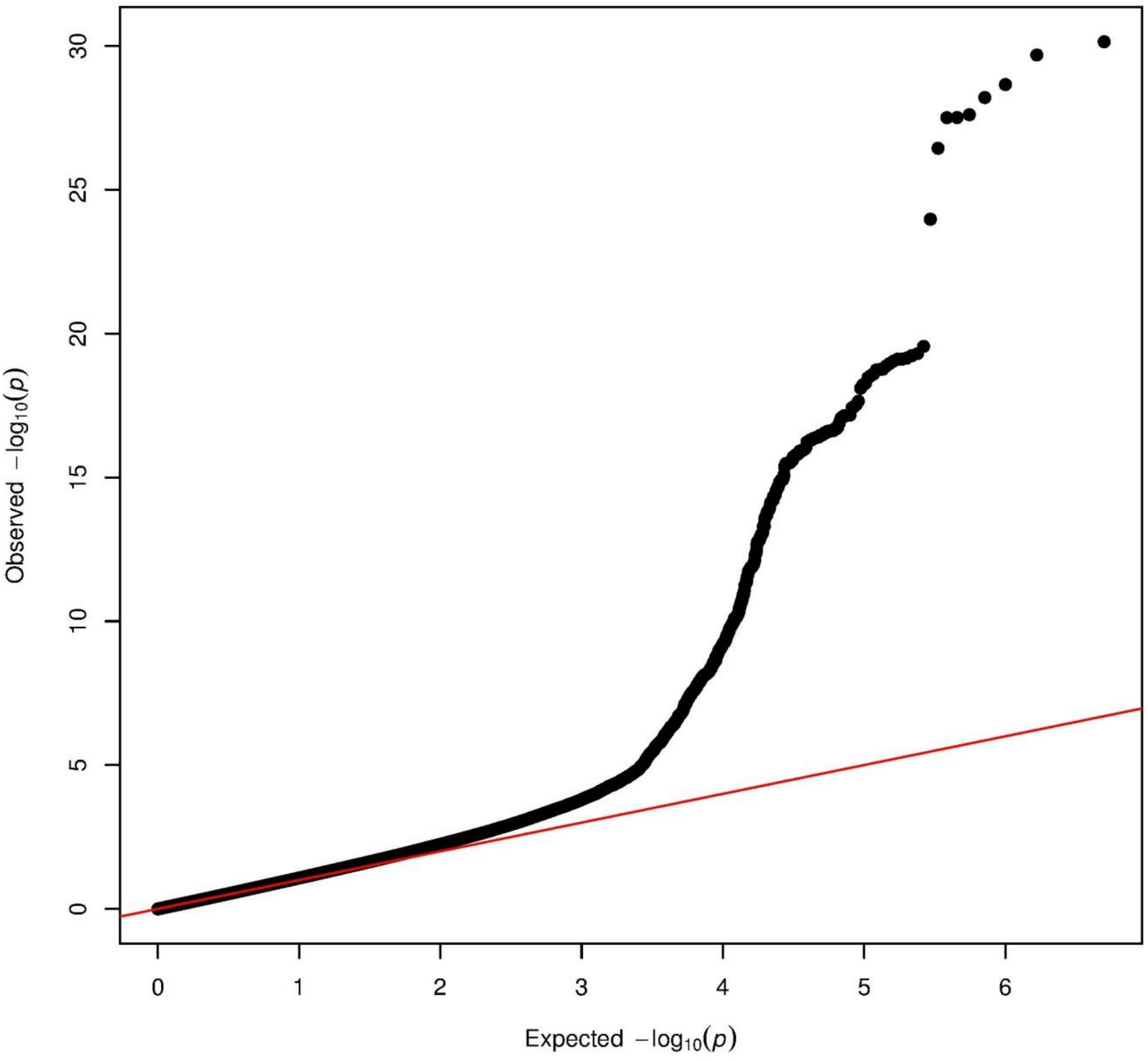

The QQ plot for association between the VCDR and all SNPs analyzed. Each black dot represents an observed statistic ($-\log_{10}P$) versus the corresponding expected statistic. The red line corresponds to the null distribution.

Supplementary Figure 2b. QQ plot of vertical cup-disc ratio in subjects of Asian ancestry

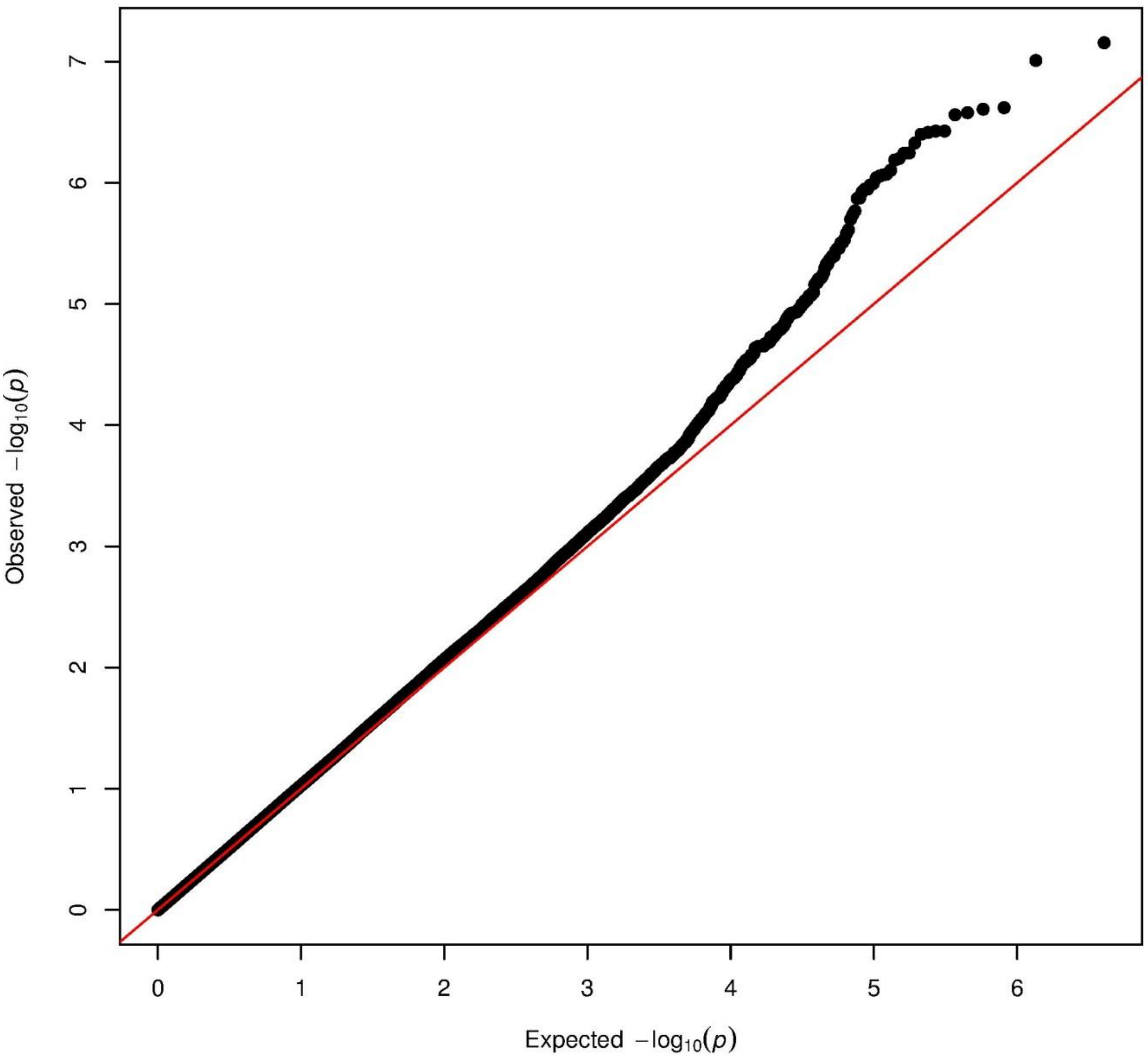

The QQ plot for association between the VCDR and all SNPs analyzed. Each black dot represents an observed statistic ($-\log_{10}P$) versus the corresponding expected statistic. The red line corresponds to the null distribution.

Supplementary Figure 2c. QQ plot of vertical cup-disc ratio in subjects of European and Asian ancestry combined

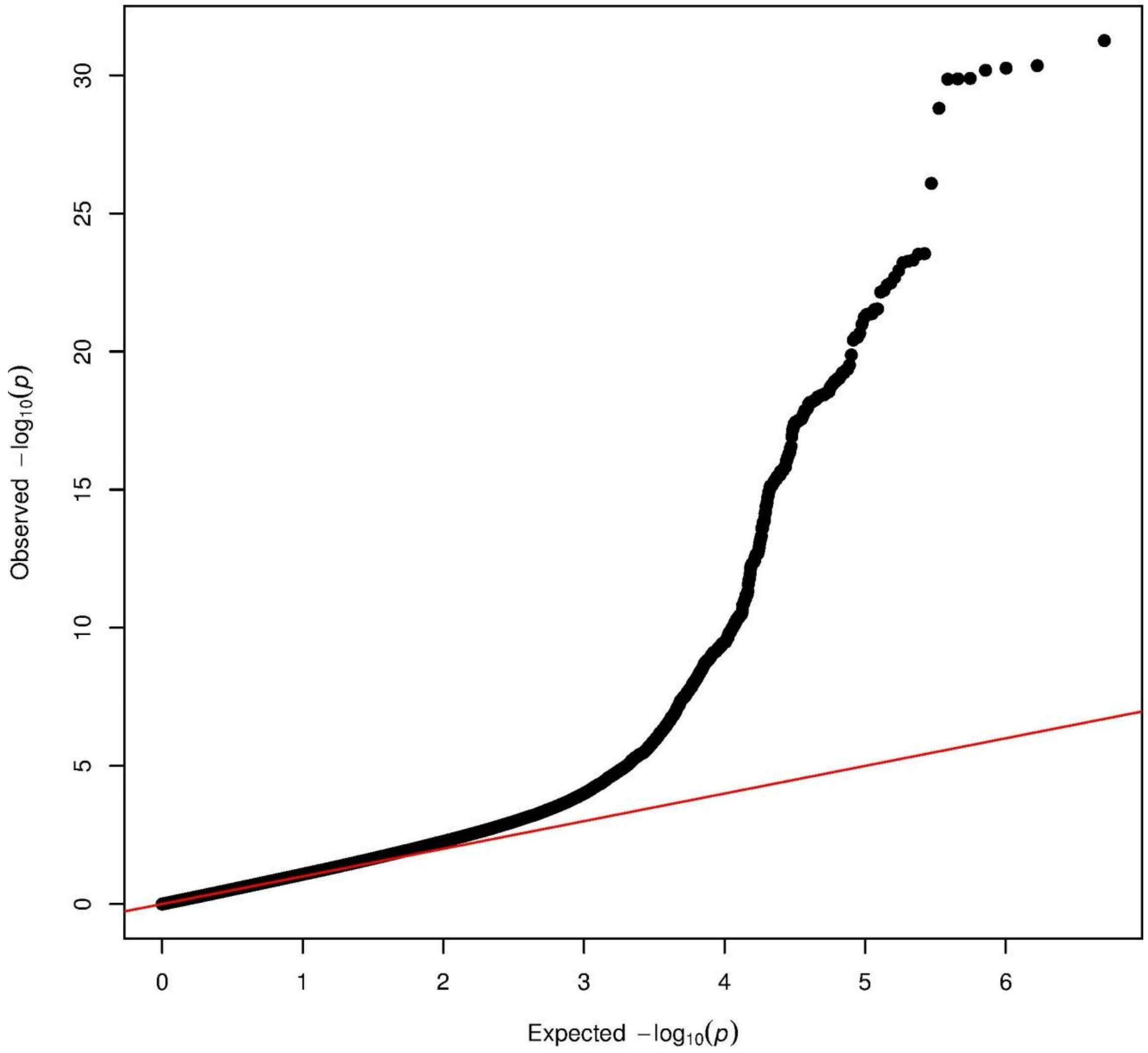

The QQ plot for association between the VCDR and all SNPs analyzed. Each black dot represents an observed statistic ($-\log_{10}P$) versus the corresponding expected statistic. The red line corresponds to the null distribution.

Supplementary Figure 3. QQ plot of vertical cup-disc ratio of each individual study

BATS

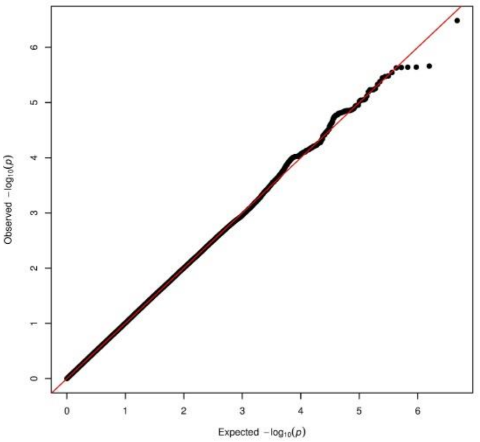

Blue Mountain Eye Study

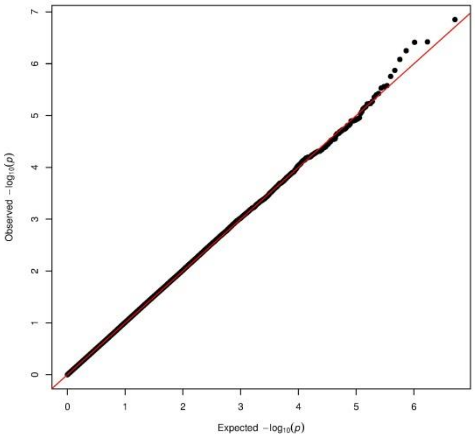

ERF

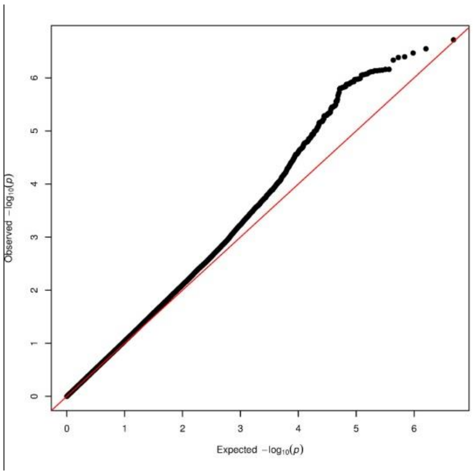

GHS I

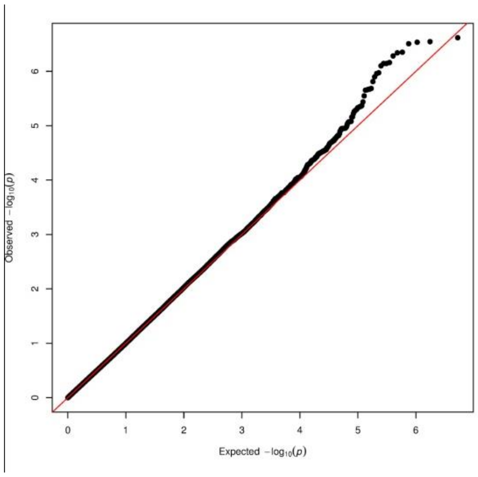

GHS II

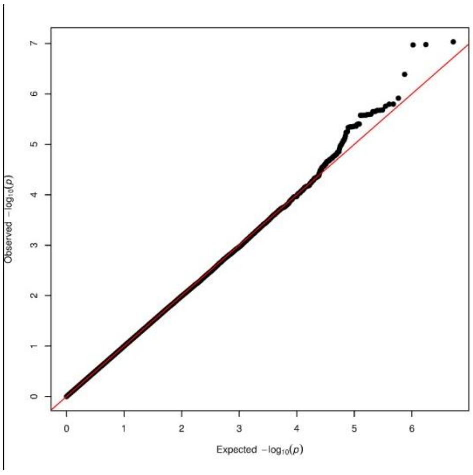

GLAUGEN (controls)

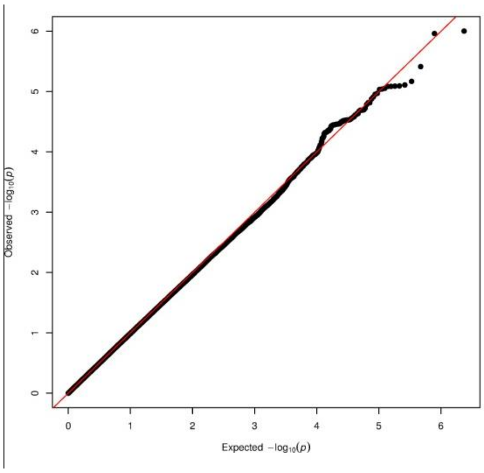

NEIGHBOR (controls)

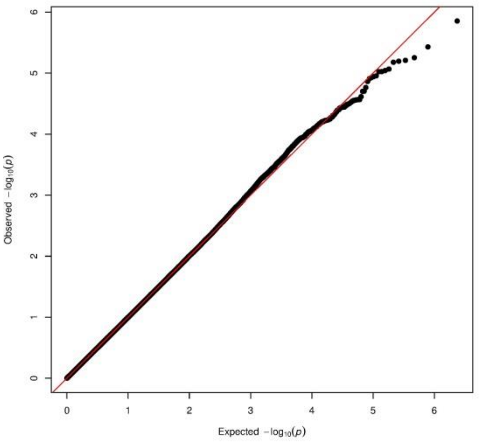

RAINE

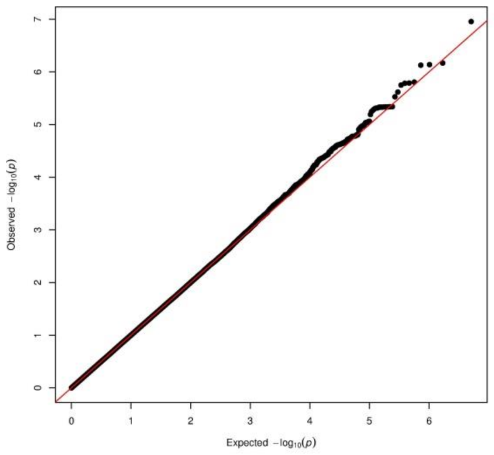

Rotterdam Study I

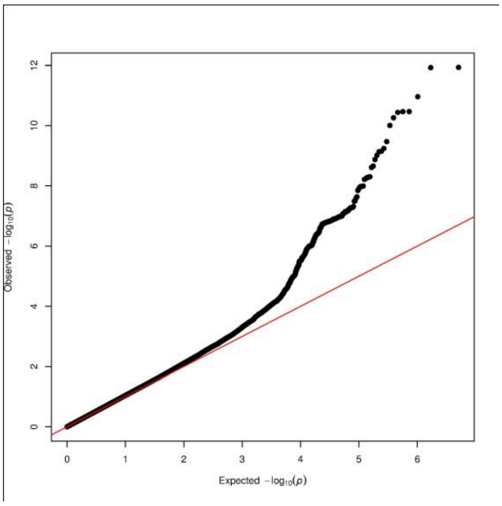

Rotterdam Study II

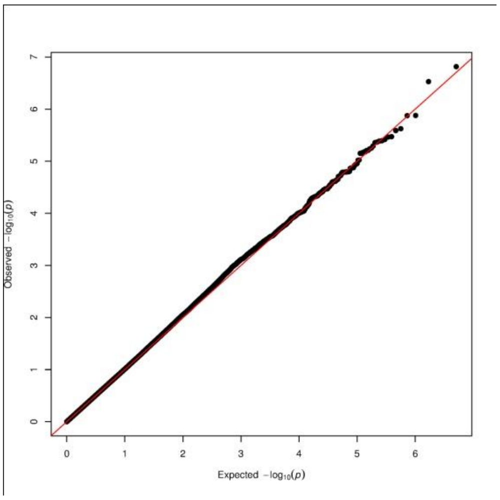

Rotterdam Study III

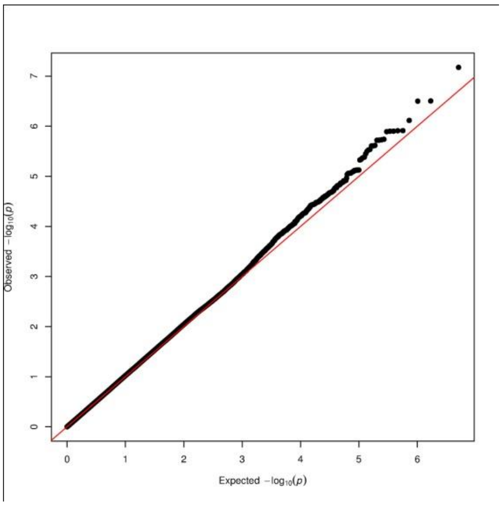

TEST

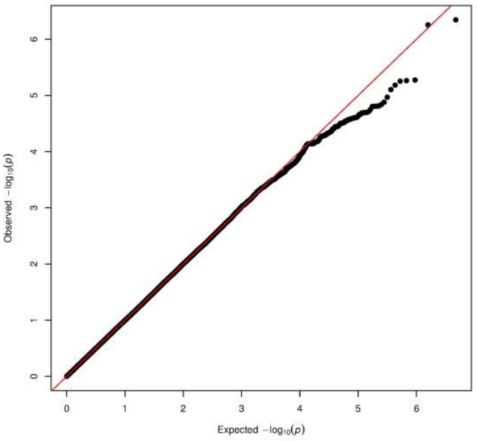

TwinsUK

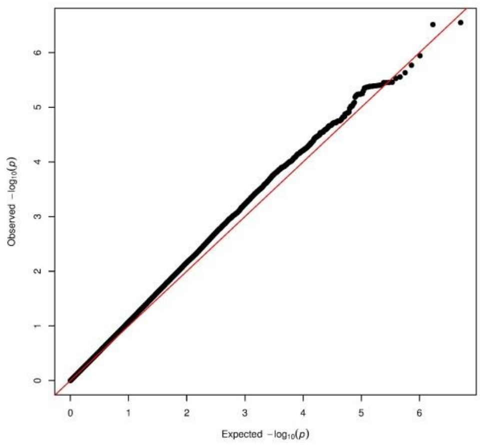

Beijing Eye Study

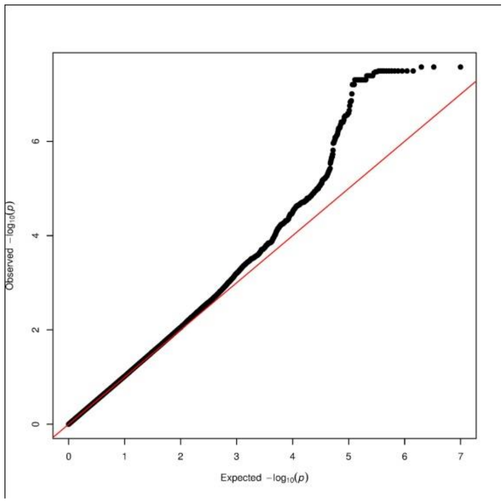

SCES

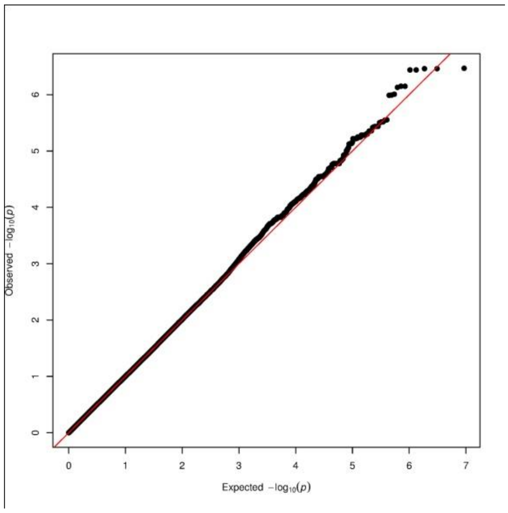

SIMES

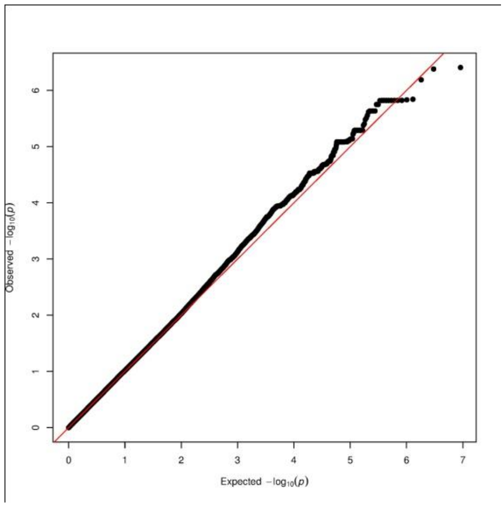

SINDI

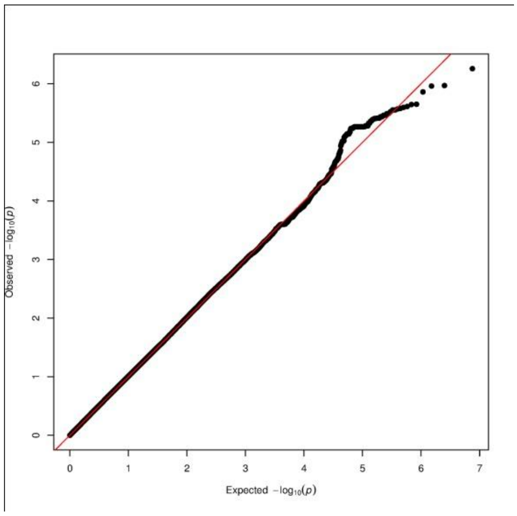

Supplementary Figure 4a. Manhattan plot of the GWAS meta-analysis for vertical cup-disc ratio in subjects of European ancestry (n = 21,094)

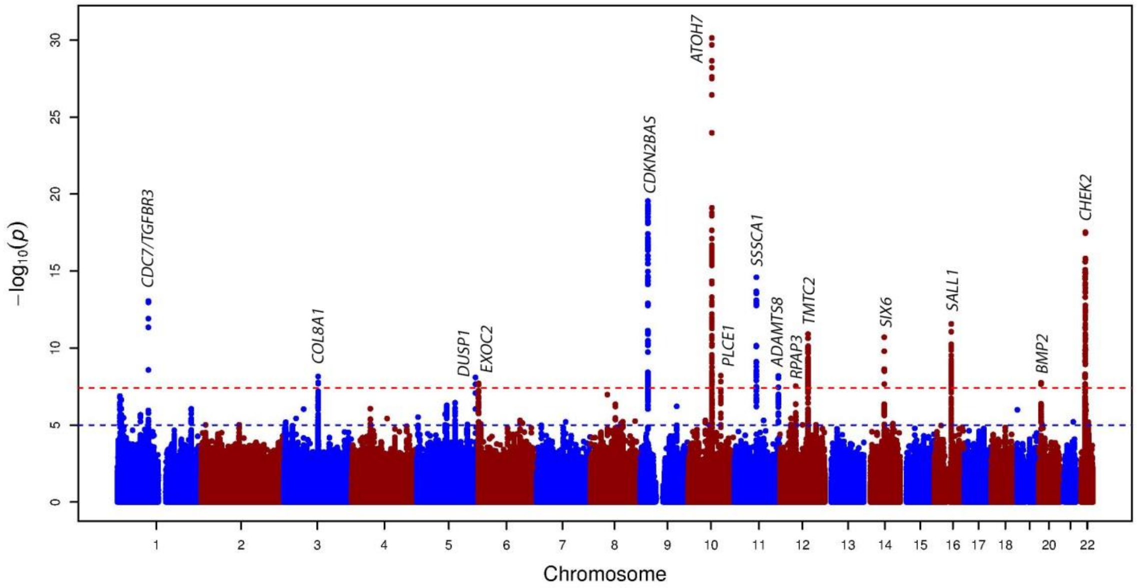

The plot shows $-\log_{10}$-transformed P values for all SNPs. The red dotted horizontal line represents the genome-wide significance threshold of $P < 5.0 \times 10^{-8}$; the blue dotted line indicates P value of 1×10^{-5}.

Supplementary Figure 4b. Manhattan plot of the GWAS meta-analysis for vertical cup-disc ratio in subjects of Asian ancestry (n = 6,784)

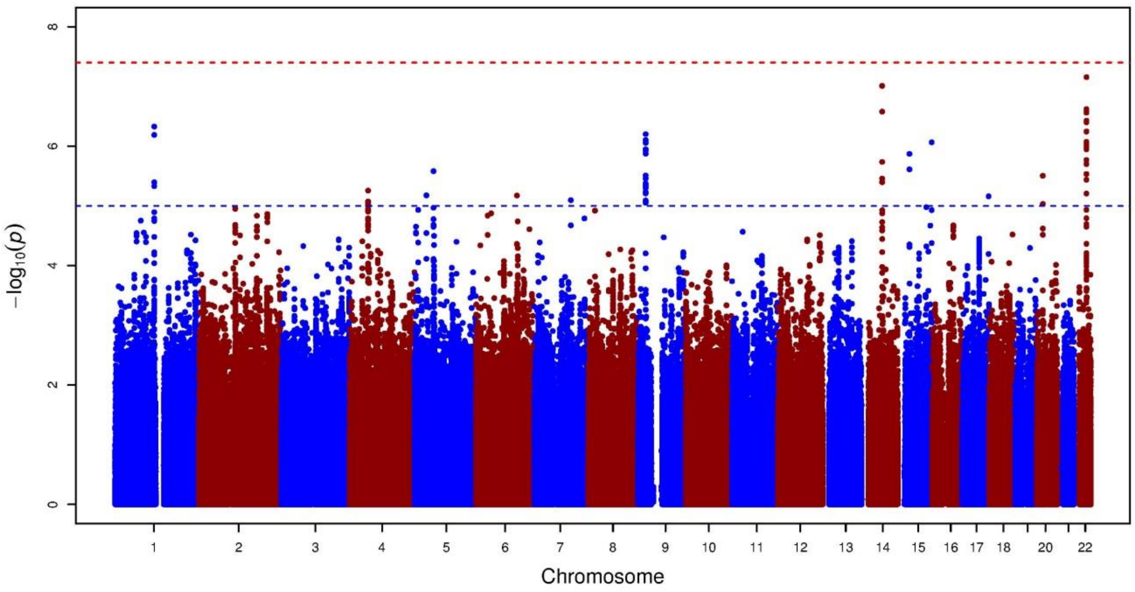

The plot shows $-\log_{10}$-transformed P values for all SNPs. The red dotted horizontal line represents the genome-wide significance threshold of $P < 5.0 \times 10^{-8}$; the blue dotted line indicates P value of 1×10^{-5}.

Supplementary Figure 5. Regional association and recombination rate plots for all 18 identified VCDR loci

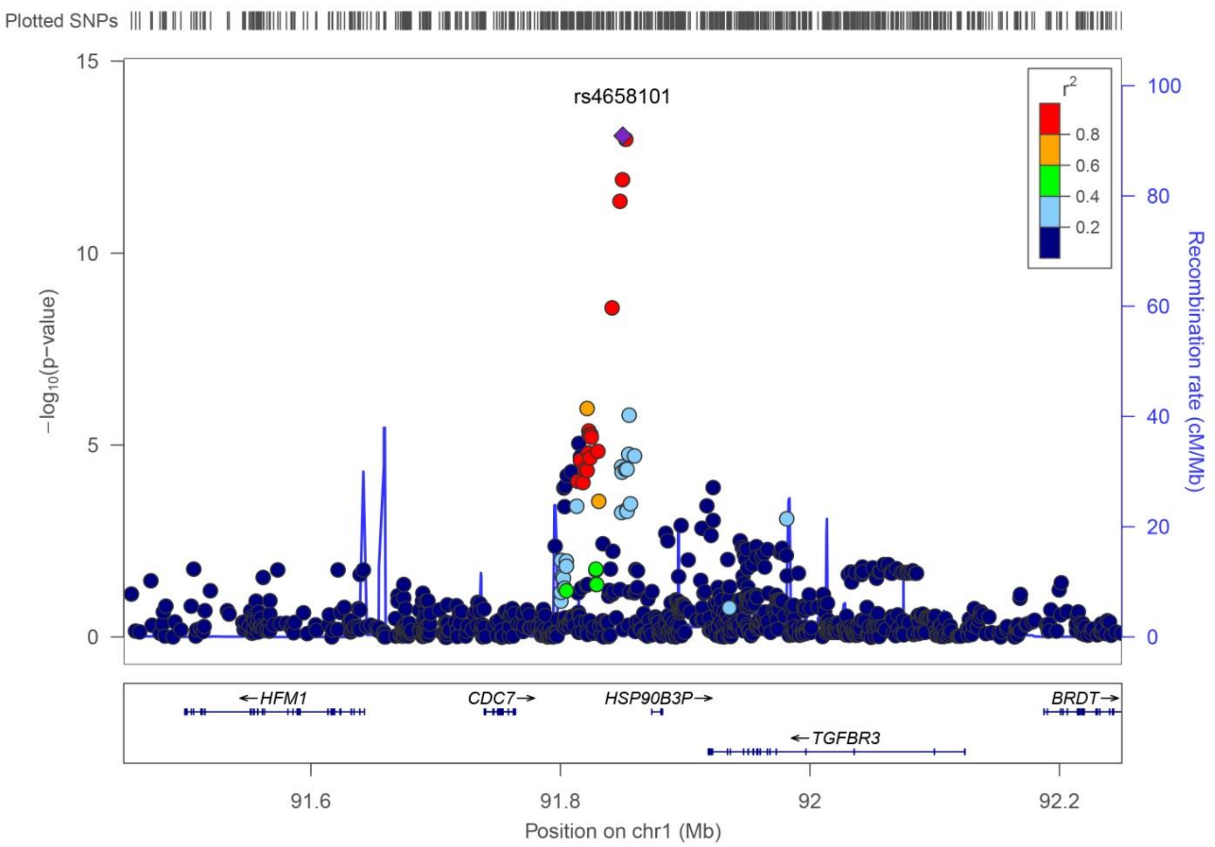

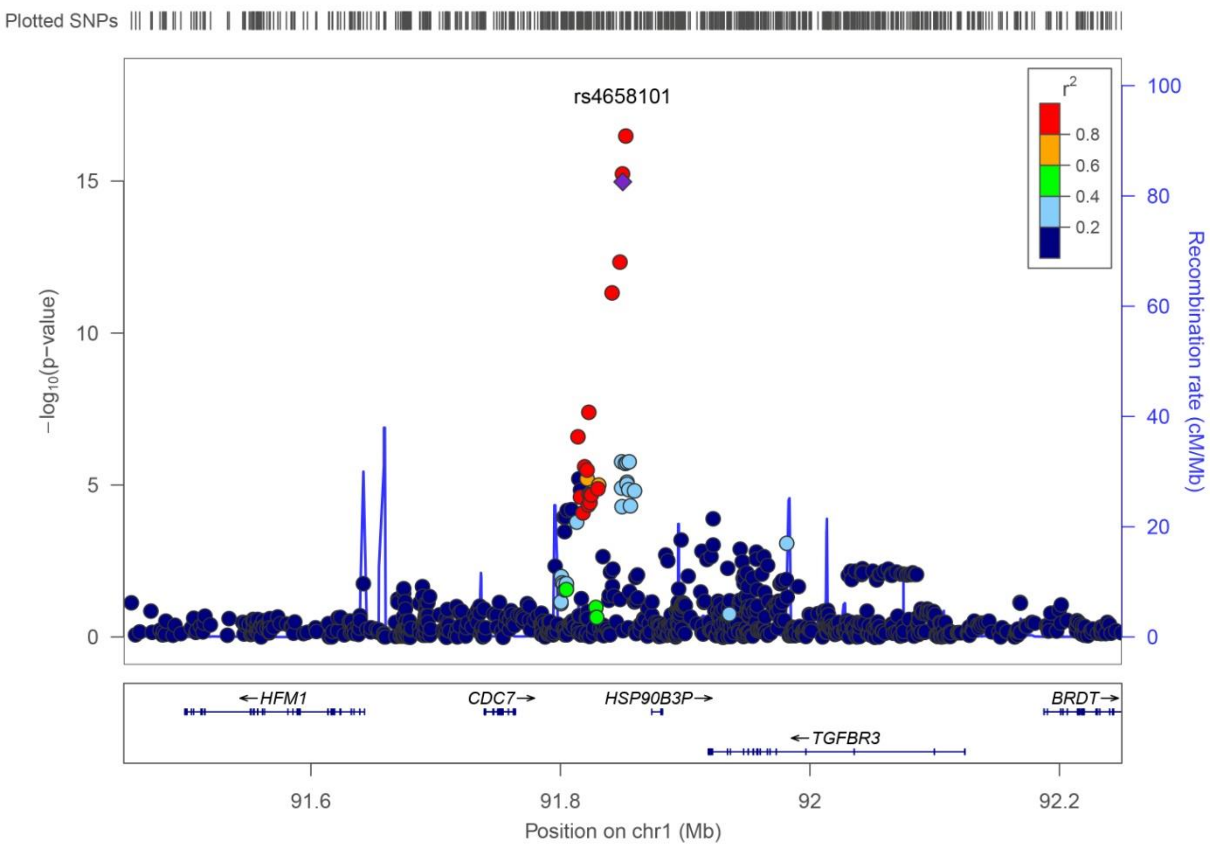

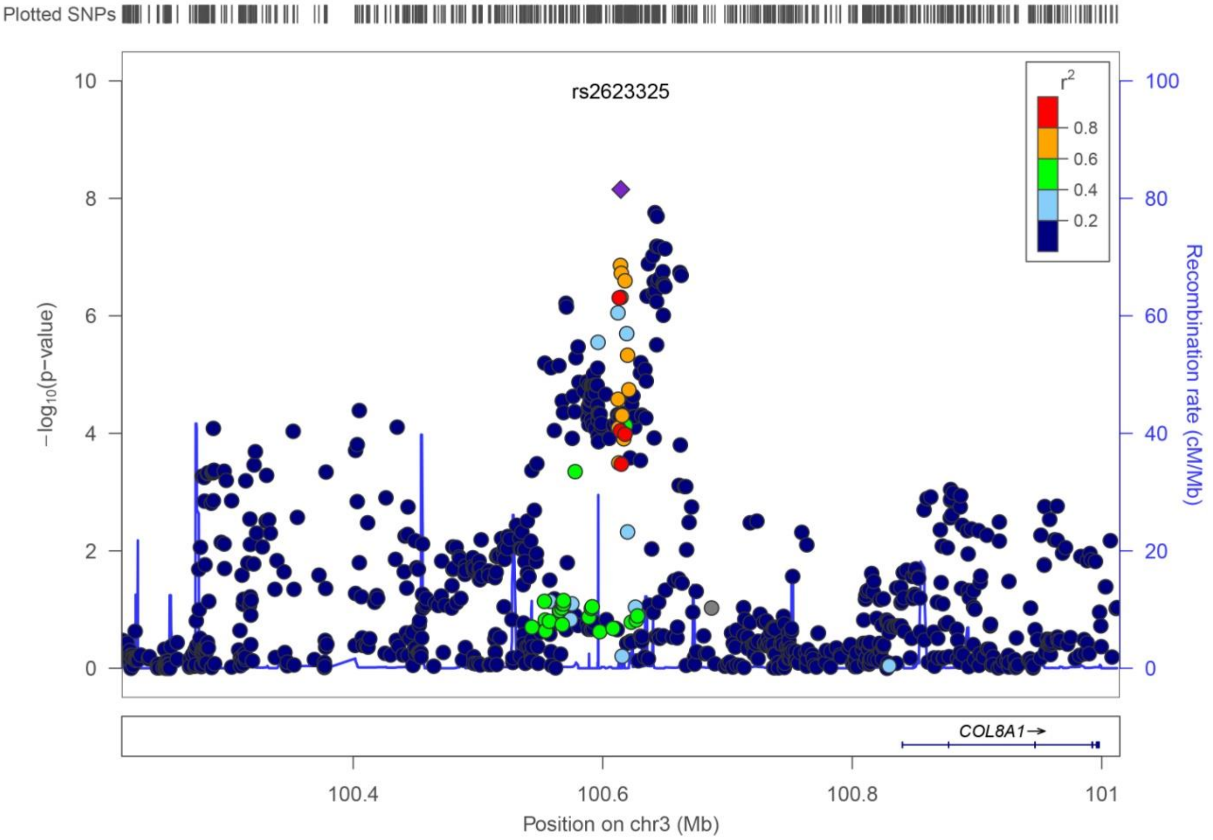
Plotted SNPs
rs2623325
r2
0.8
0.6
0.4
0.2
−log10(p−value)
Recombination rate (cM/Mb)
COL8A1
100.4
100.6
100.8
101
Position on chr3 (Mb)

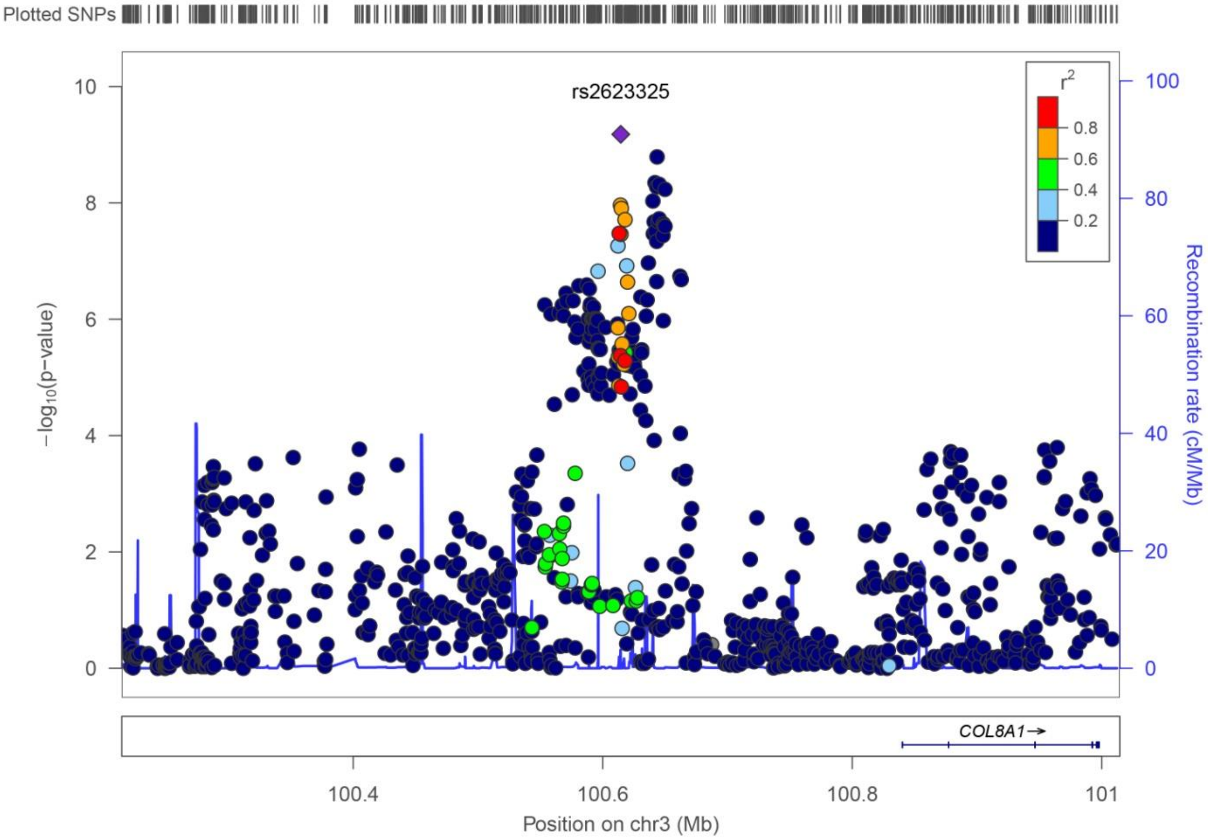
Plotted SNPs
rs2623325
r2
0.8
0.6
0.4
0.2
−log10(p−value)
Recombination rate (cM/Mb)
COL8A1
100.4
100.6
100.8
101
Position on chr3 (Mb)

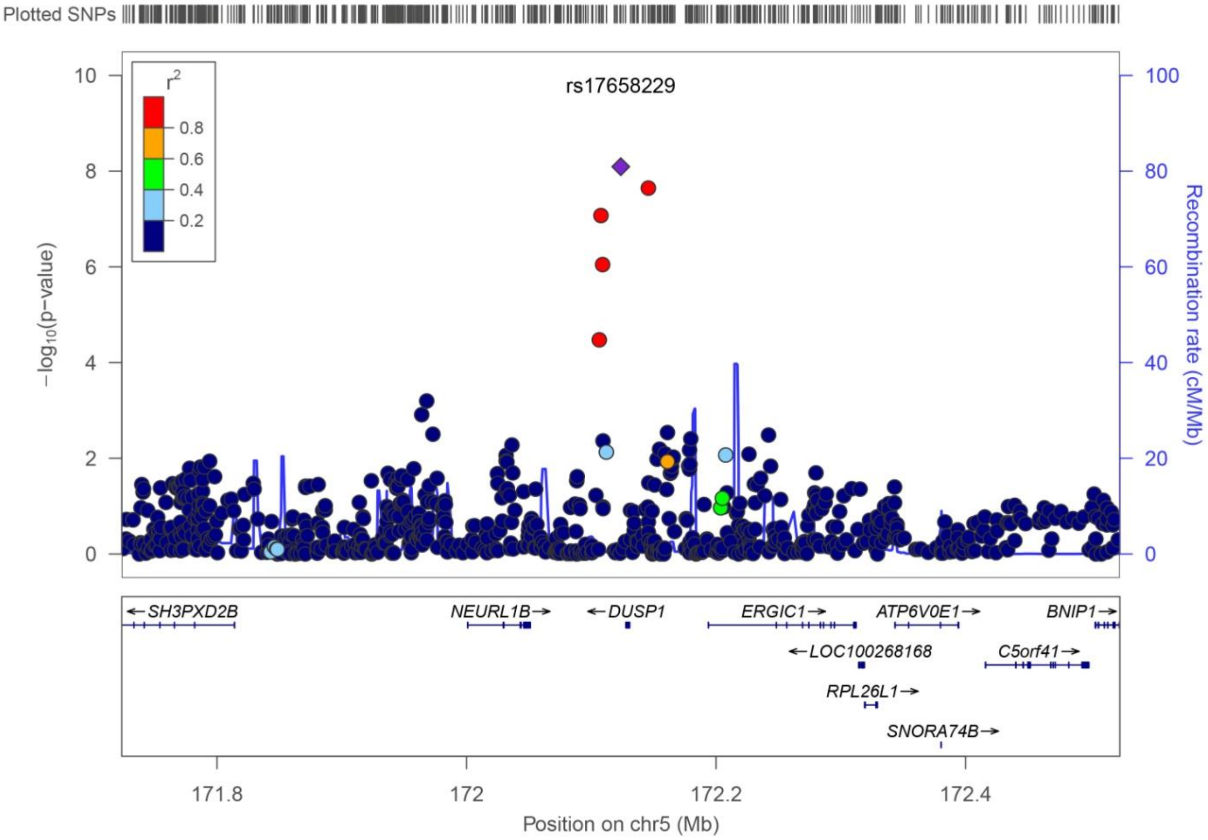
Plotted SNPs
rs17658229
r2
0.8
0.6
0.4
0.2
−log10(p−value)
Recombination rate (cM/Mb)
SH3PXD2B
NEURL1B
DUSP1
ERGIC1
ATP6V0E1
BNIP1
LOC100268168
C5orf41
RPL26L1
SNORA74B
171.8
172
172.2
172.4
Position on chr5 (Mb)

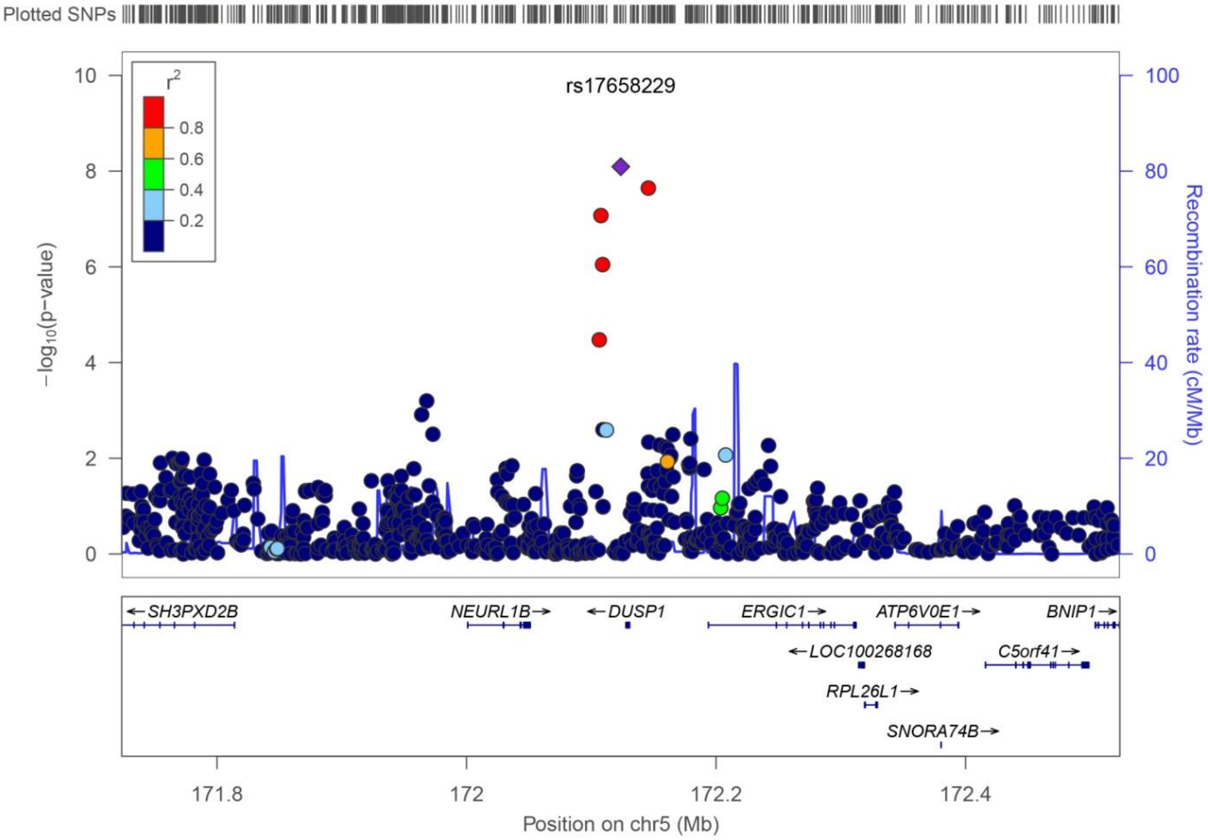
Plotted SNPs
rs17658229
r2
0.8
0.6
0.4
0.2
−log10(p−value)
Recombination rate (cM/Mb)
SH3PXD2B
NEURL1B
DUSP1
ERGIC1
ATP6V0E1
BNIP1
LOC100268168
C5orf41
RPL26L1
SNORA74B
171.8
172
172.2
172.4
Position on chr5 (Mb)

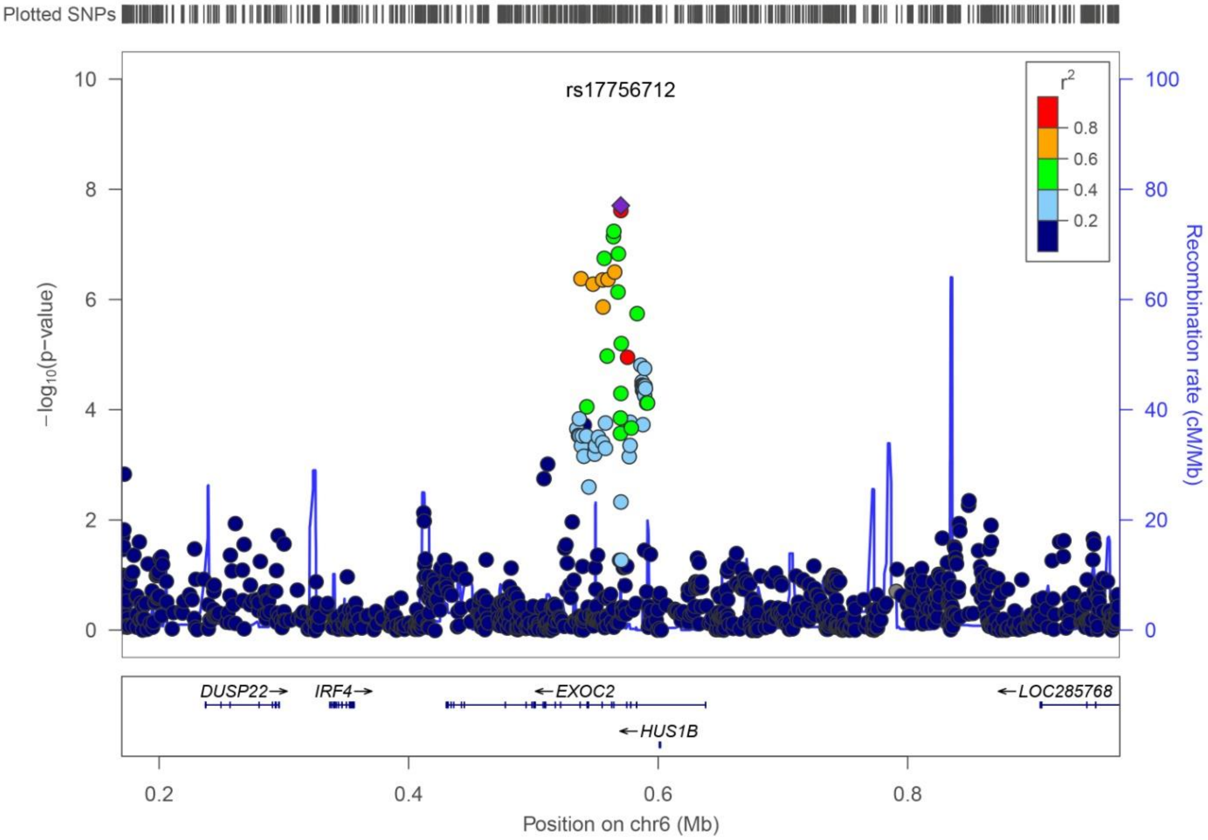
Plotted SNPs
rs17756712
10
8
6
4
2
0
−log10(p−value)
r2
0.8
0.6
0.4
0.2
100
80
60
40
20
0
Recombination rate (cM/Mb)
DUSP22
IRF4
EXOC2
LOC285768
HUS1B
0.2
0.4
0.6
0.8
Position on chr6 (Mb)

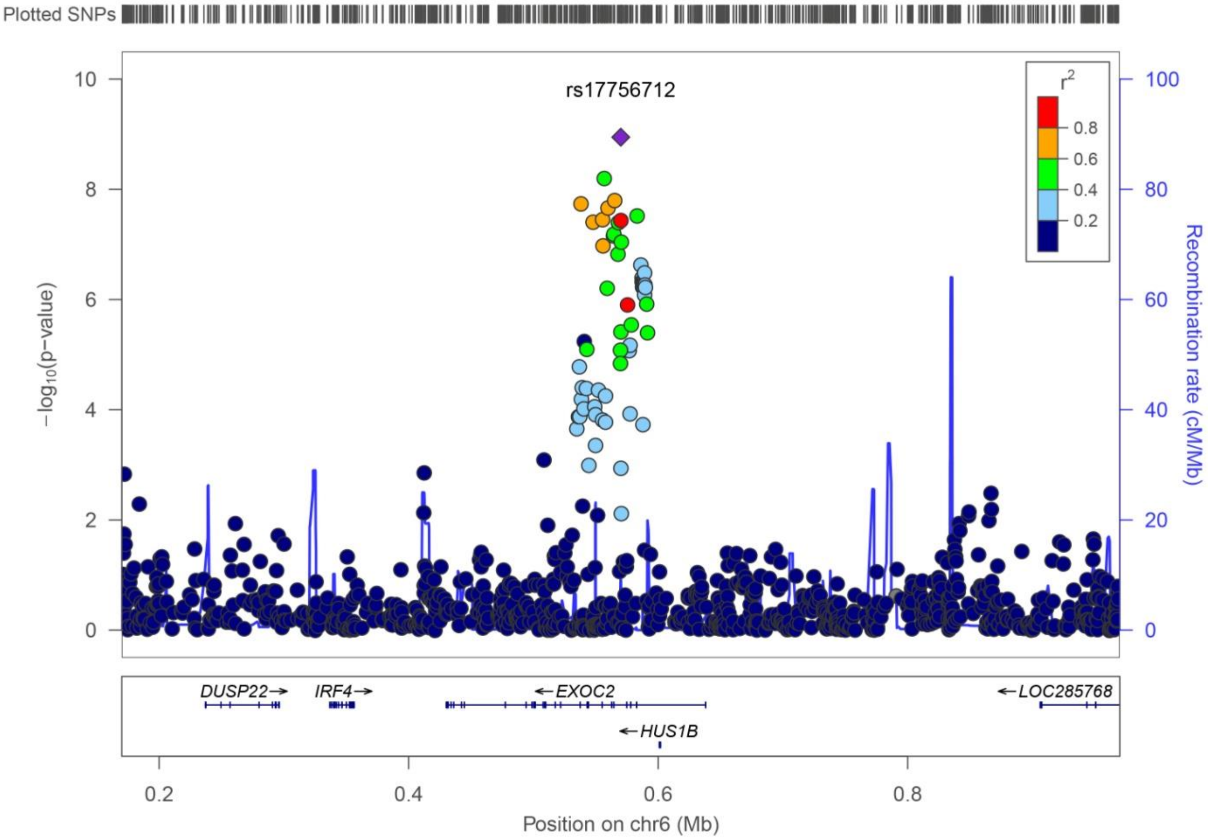
Plotted SNPs
rs17756712
10
8
6
4
2
0
−log10(p−value)
r2
0.8
0.6
0.4
0.2
100
80
60
40
20
0
Recombination rate (cM/Mb)
DUSP22
IRF4
EXOC2
LOC285768
HUS1B
0.2
0.4
0.6
0.8
Position on chr6 (Mb)

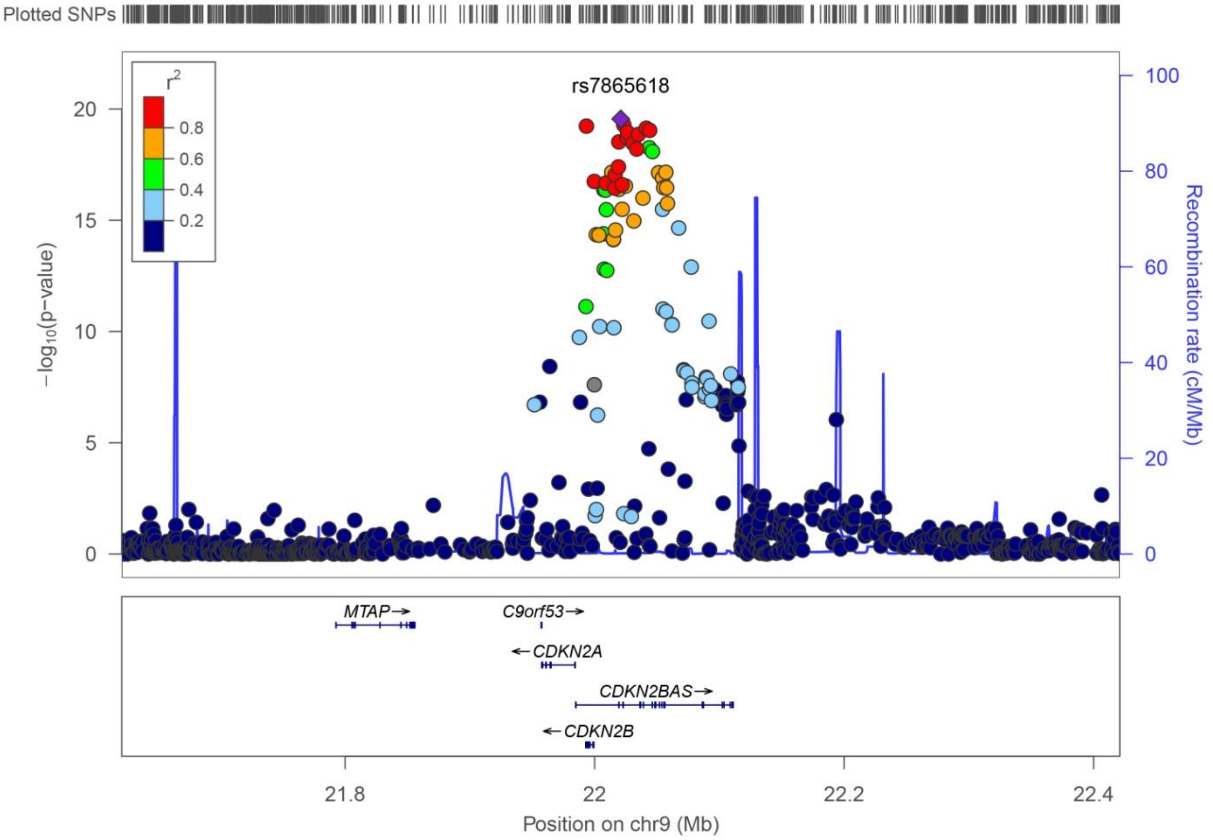
Plotted SNPs
rs7865618
r²
0.8
0.6
0.4
0.2
−log10(p−value)
Recombination rate (cM/Mb)
MTAP
C9orf53
CDKN2A
CDKN2BAS
CDKN2B
Position on chr9 (Mb)
21.8
22
22.2
22.4

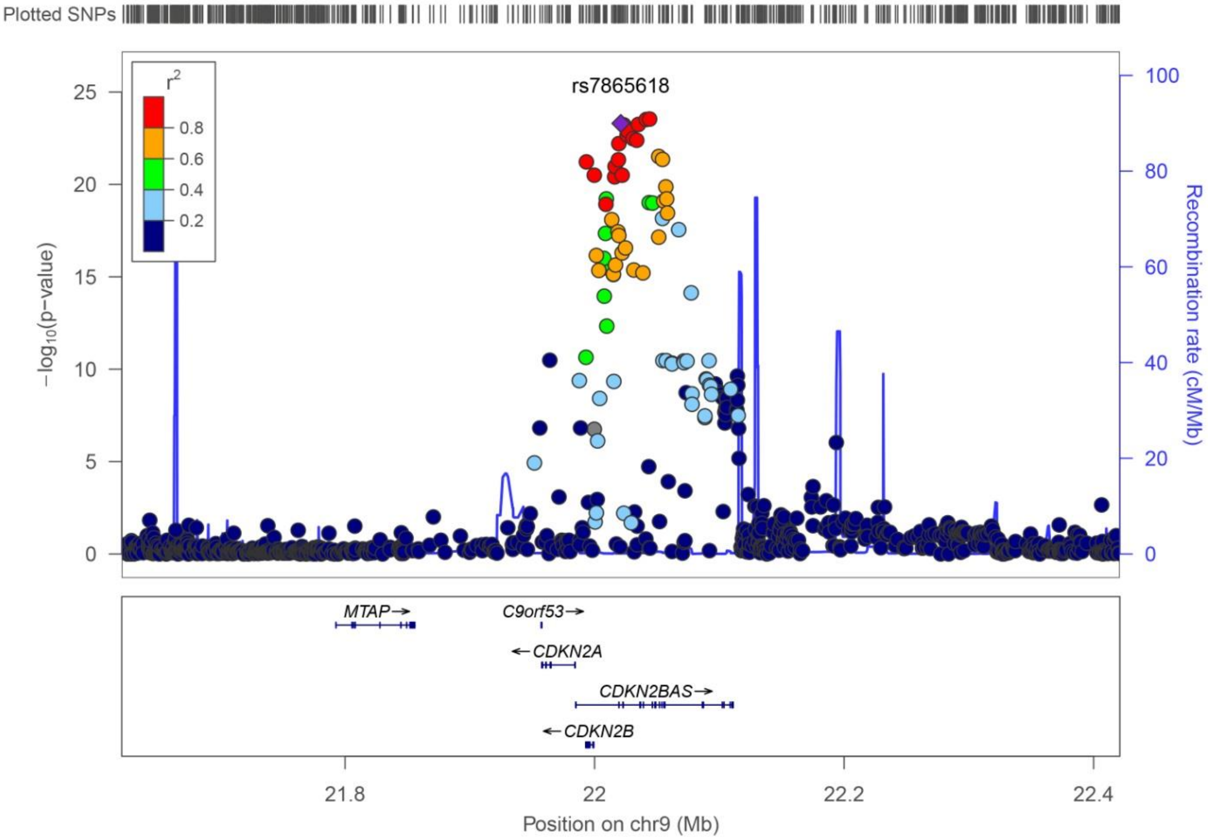
Plotted SNPs
rs7865618
r²
0.8
0.6
0.4
0.2
−log10(p−value)
Recombination rate (cM/Mb)
MTAP
C9orf53
CDKN2A
CDKN2BAS
CDKN2B
Position on chr9 (Mb)
21.8
22
22.2
22.4

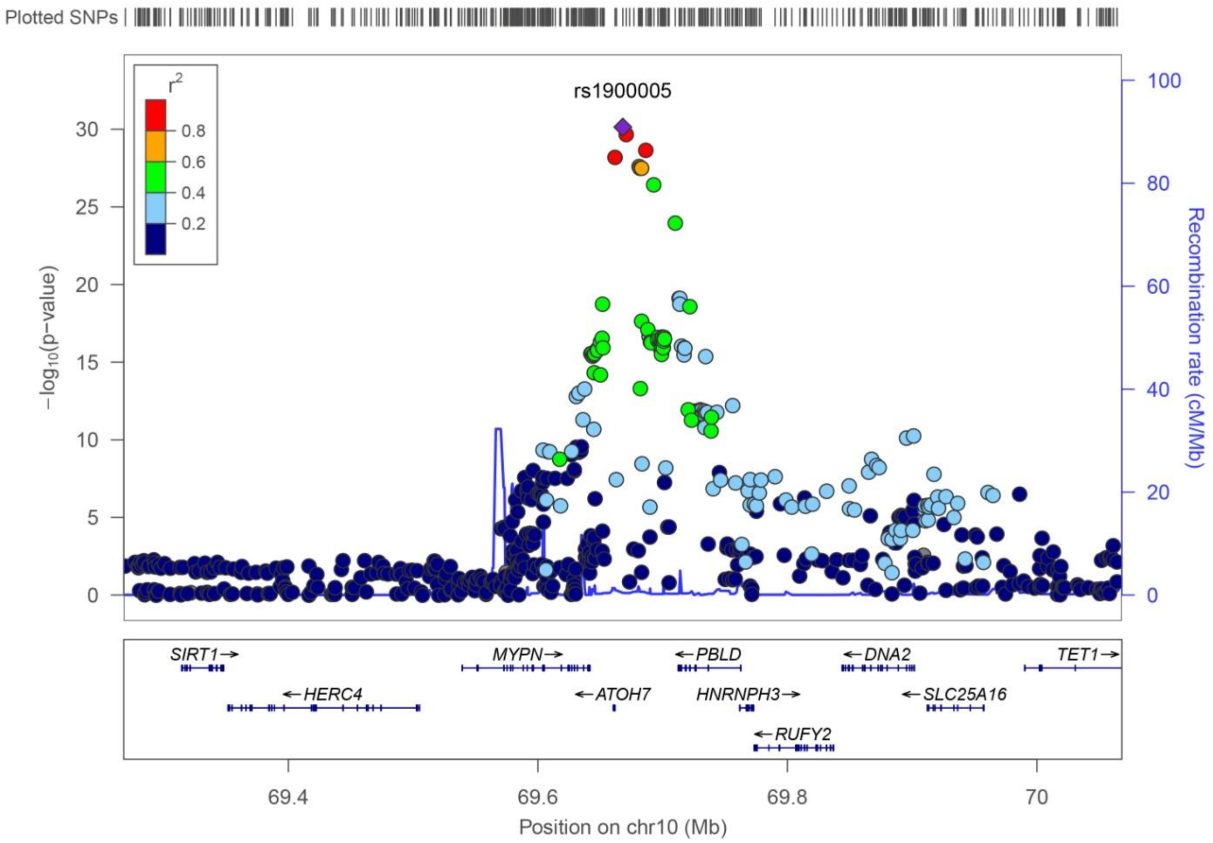
Plotted SNPs
rs1900005
r2
0.8
0.6
0.4
0.2
−log10(p−value)
Recombination rate (cM/Mb)
SIRT1→
MYPN→
←PBLD
←DNA2
TET1→
←HERC4
←ATOH7
HNRNPH3→
←SLC25A16
←RUFY2
69.4
69.6
69.8
70
Position on chr10 (Mb)

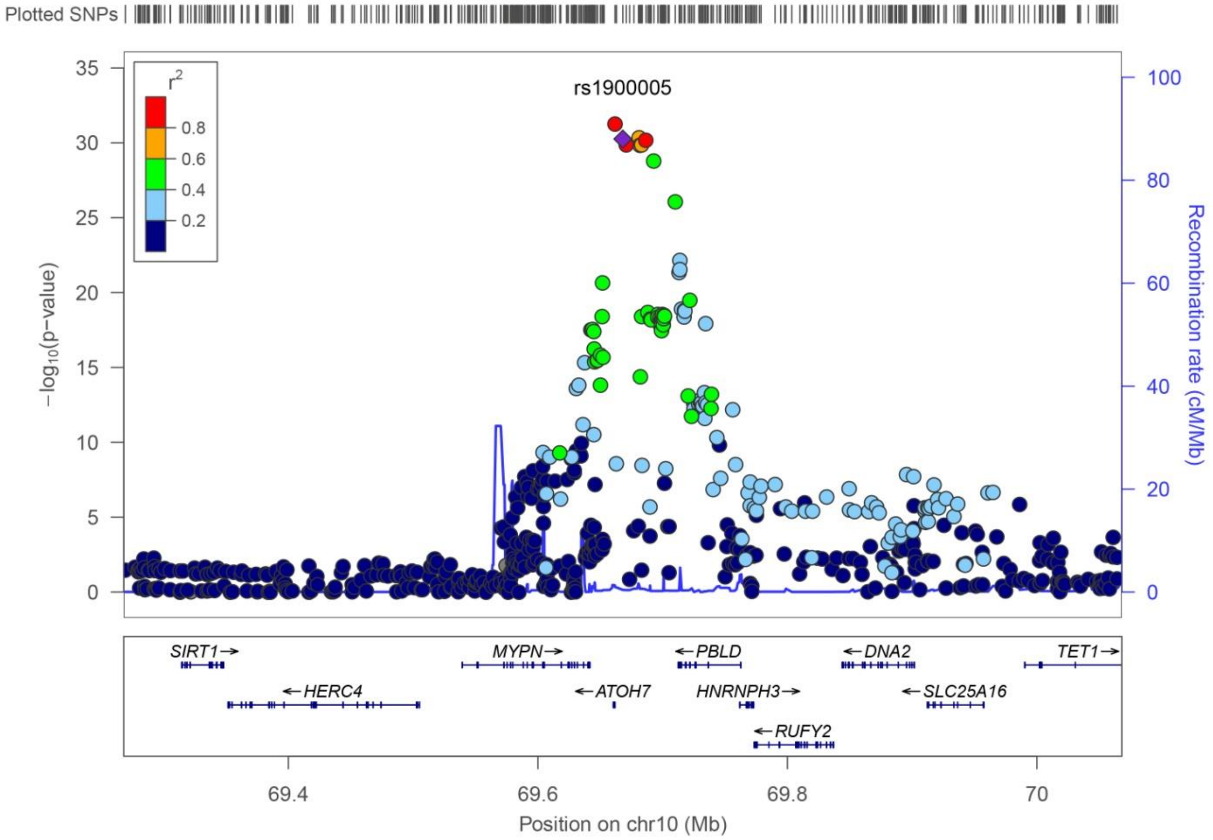
Plotted SNPs
rs1900005
r2
0.8
0.6
0.4
0.2
−log10(p−value)
Recombination rate (cM/Mb)
SIRT1→
MYPN→
←PBLD
←DNA2
TET1→
←HERC4
←ATOH7
HNRNPH3→
←SLC25A16
←RUFY2
69.4
69.6
69.8
70
Position on chr10 (Mb)

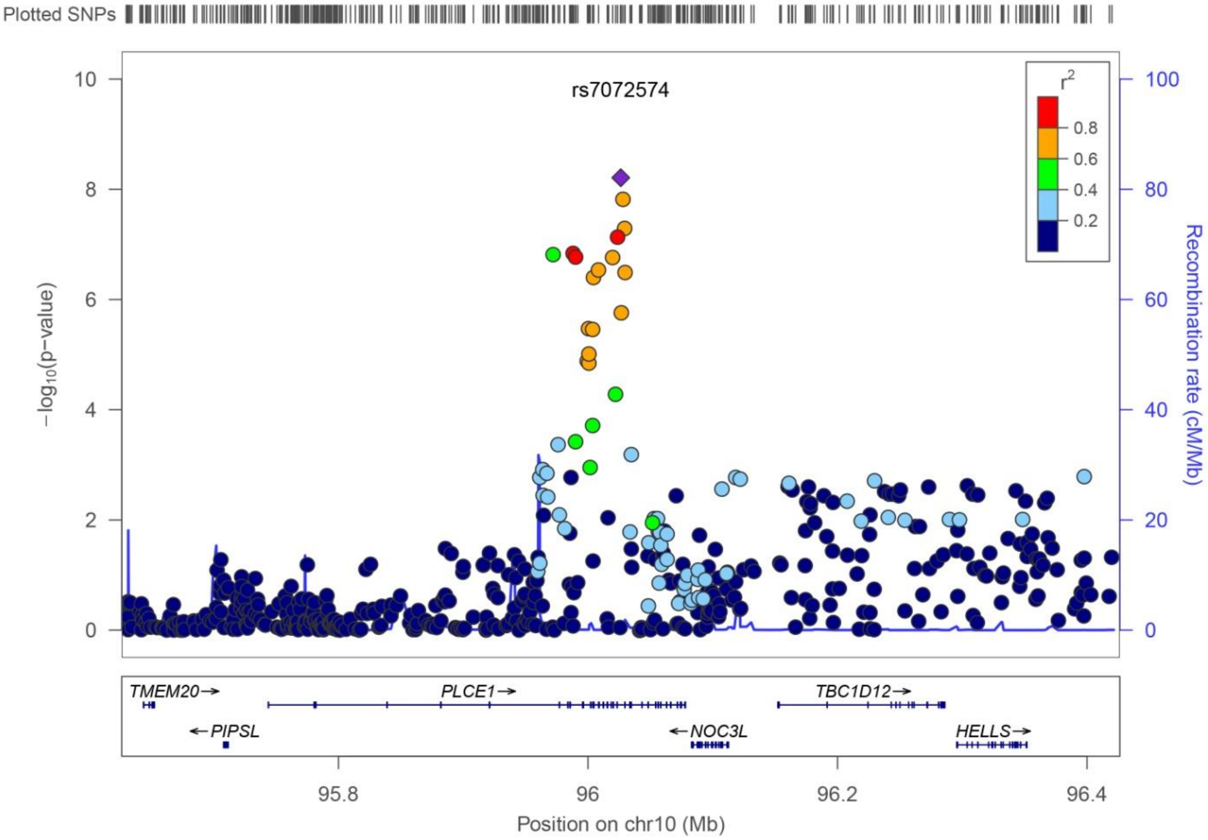

Plotted SNPs
rs7072574
r2
0.8
0.6
0.4
0.2
−log10(p−value)
Recombination rate (cM/Mb)
TMEM20→
PLCE1→
TBC1D12→
←PIPSL
←NOC3L
HELLS→
95.8
96
96.2
96.4
Position on chr10 (Mb)

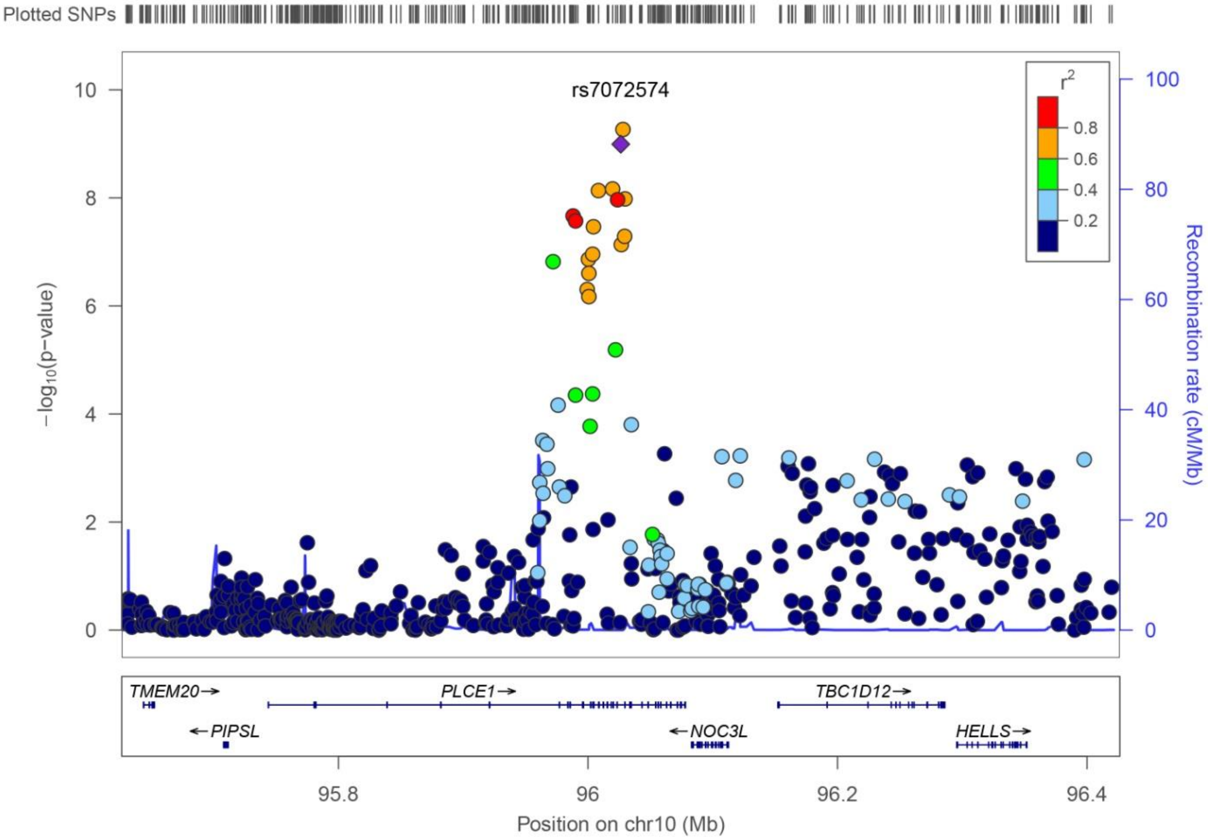

Plotted SNPs
rs7072574
r2
0.8
0.6
0.4
0.2
−log10(p−value)
Recombination rate (cM/Mb)
TMEM20→
PLCE1→
TBC1D12→
←PIPSL
←NOC3L
HELLS→
95.8
96
96.2
96.4
Position on chr10 (Mb)

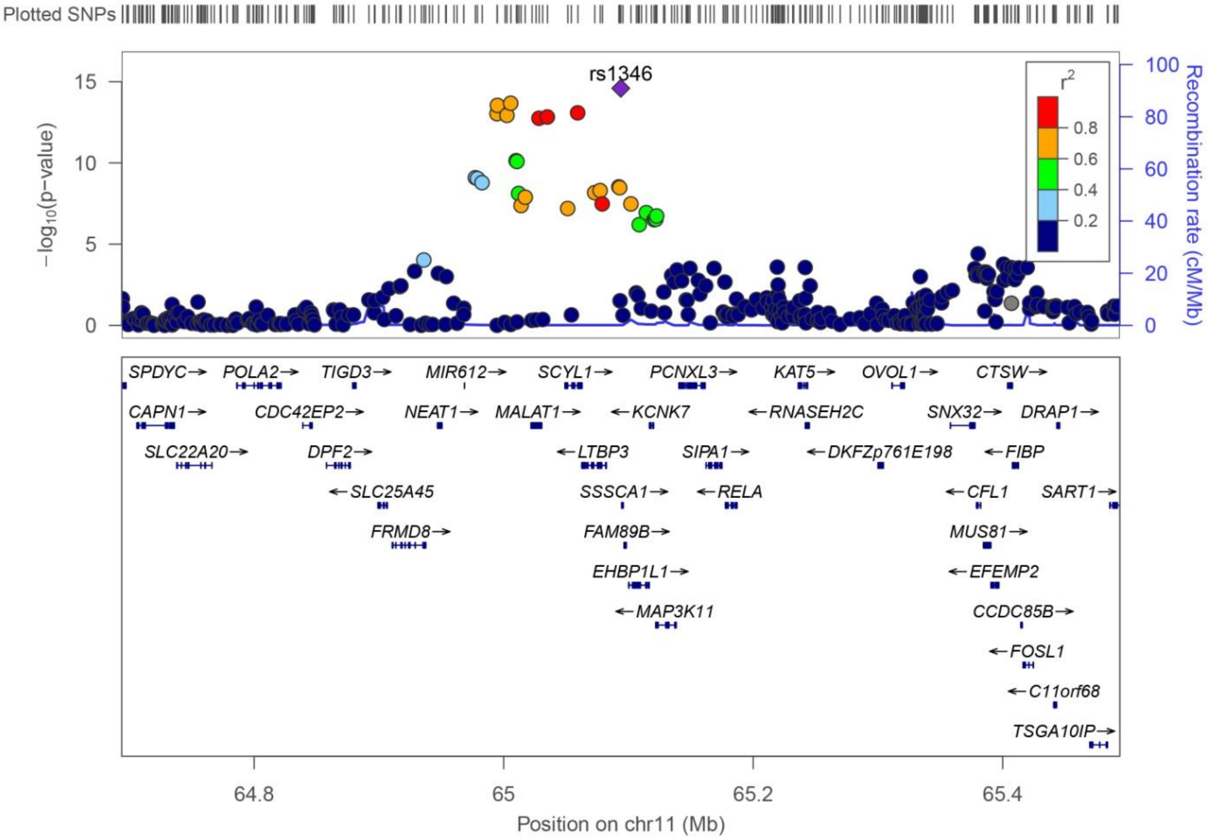
Plotted SNPs
rs1346
−log10(p−value)
Recombination rate (cM/Mb)
r2
0.8
0.6
0.4
0.2
SPDYC→ POLA2→ TIGD3→ MIR612→ SCYL1→ PCNXL3→ KAT5→ OVOL1→ CTSW→
CAPN1→ CDC42EP2→ NEAT1→ MALAT1→ ←KCNK7 ←RNASEH2C SNX32→ DRAP1→
SLC22A20→ DPF2→ ←LTBP3 SIPA1→ ←DKFZp761E198 ←FIBP
←SLC25A45 SSSCA1→ ←RELA ←CFL1 SART1→
FRMD8→ FAM89B→ MUS81→
EHBP1L1→ ←EFEMP2
←MAP3K11 CCDC85B→
←FOSL1
←C11orf68
TSGA10IP→
64.8
65
65.2
65.4
Position on chr11 (Mb)

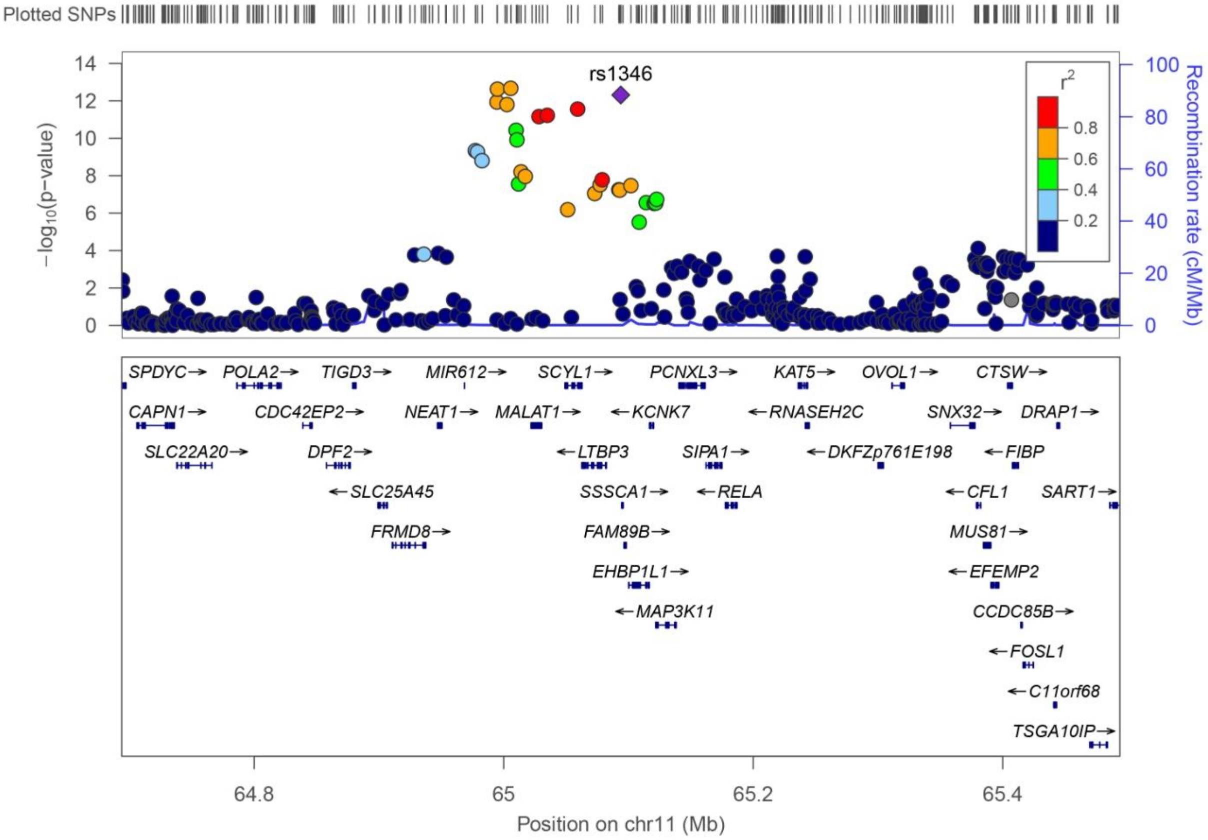
Plotted SNPs
rs1346
−log10(p−value)
Recombination rate (cM/Mb)
r2
0.8
0.6
0.4
0.2
SPDYC→ POLA2→ TIGD3→ MIR612→ SCYL1→ PCNXL3→ KAT5→ OVOL1→ CTSW→
CAPN1→ CDC42EP2→ NEAT1→ MALAT1→ ←KCNK7 ←RNASEH2C SNX32→ DRAP1→
SLC22A20→ DPF2→ ←LTBP3 SIPA1→ ←DKFZp761E198 ←FIBP
←SLC25A45 SSSCA1→ ←RELA ←CFL1 SART1→
FRMD8→ FAM89B→ MUS81→
EHBP1L1→ ←EFEMP2
←MAP3K11 CCDC85B→
←FOSL1
←C11orf68
TSGA10IP→
64.8
65
65.2
65.4
Position on chr11 (Mb)

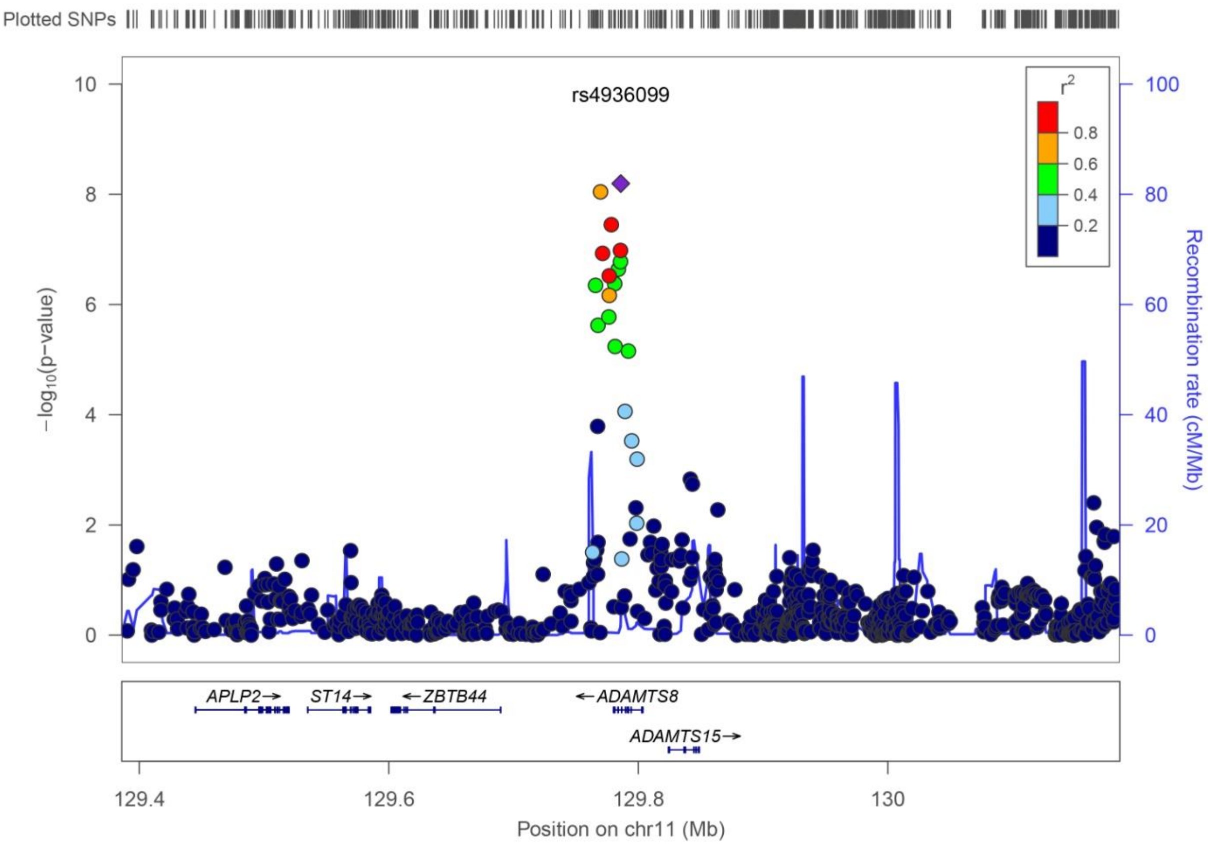
Plotted SNPs
rs4936099
r2
0.8
0.6
0.4
0.2
−log10(p−value)
Recombination rate (cM/Mb)
APLP2→
ST14→
←ZBTB44
←ADAMTS8
ADAMTS15→
129.4
129.6
129.8
130
Position on chr11 (Mb)

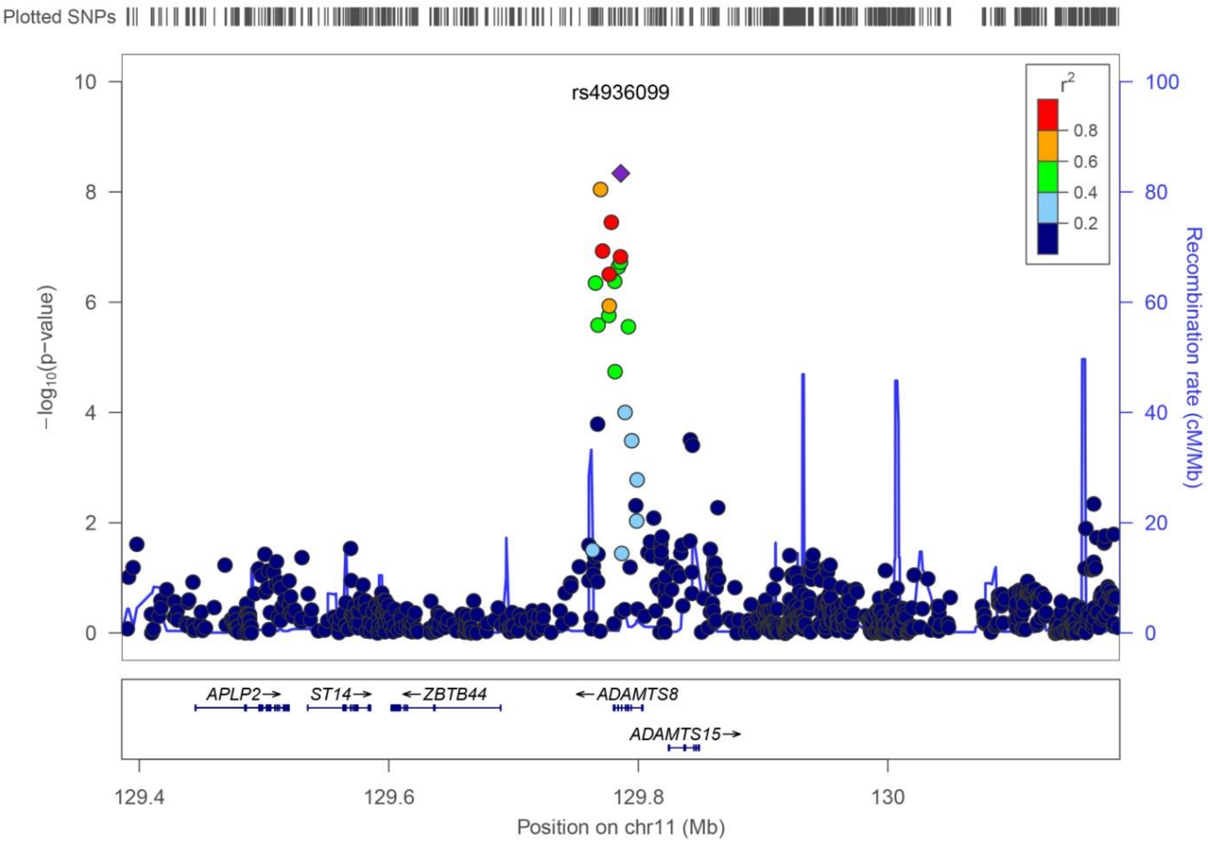
Plotted SNPs
rs4936099
r2
0.8
0.6
0.4
0.2
−log10(p−value)
Recombination rate (cM/Mb)
APLP2→
ST14→
←ZBTB44
←ADAMTS8
ADAMTS15→
129.4
129.6
129.8
130
Position on chr11 (Mb)

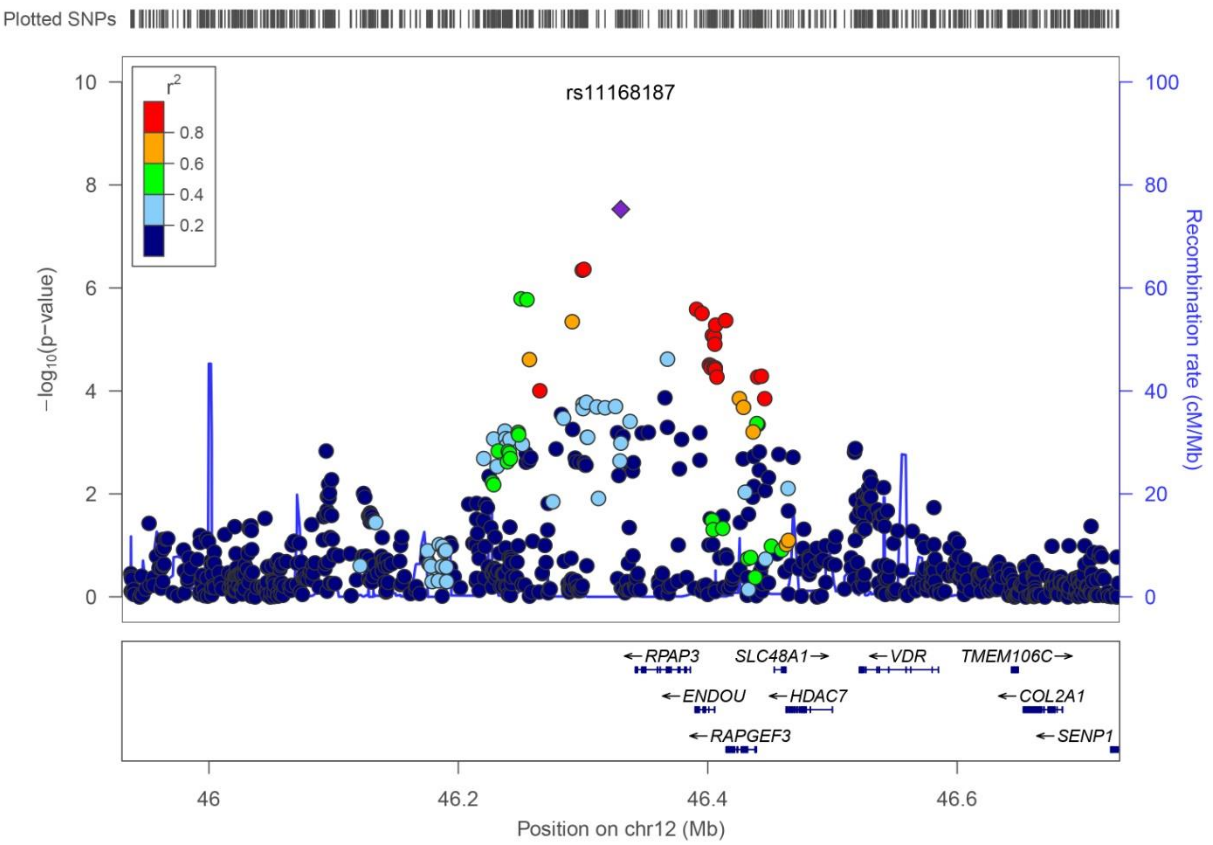

Plotted SNPs
rs11168187
r2
0.8
0.6
0.4
0.2
−log10(p−value)
Recombination rate (cM/Mb)
RPAP3
SLC48A1
VDR
TMEM106C
ENDOU
HDAC7
COL2A1
RAPGEF3
SENP1
Position on chr12 (Mb)

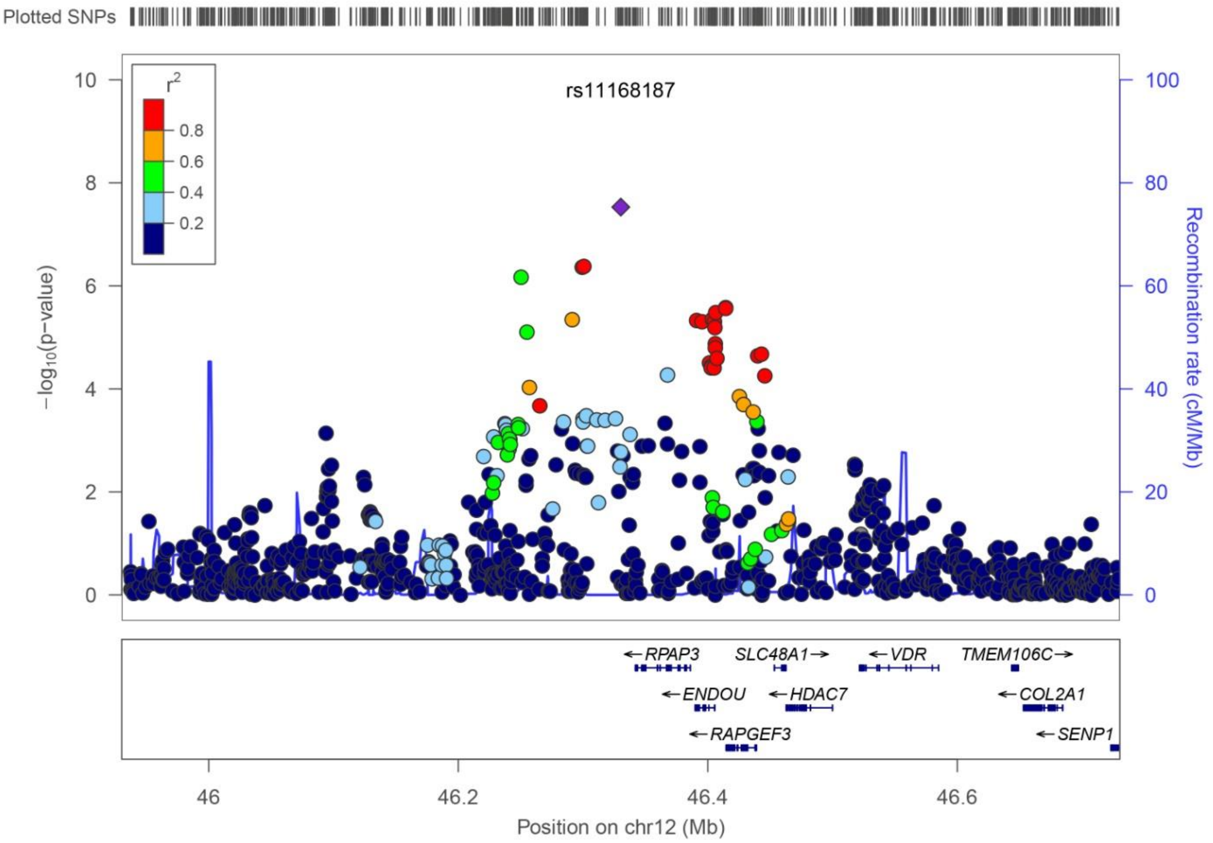

Plotted SNPs
rs11168187
r2
0.8
0.6
0.4
0.2
−log10(p−value)
Recombination rate (cM/Mb)
RPAP3
SLC48A1
VDR
TMEM106C
ENDOU
HDAC7
COL2A1
RAPGEF3
SENP1
Position on chr12 (Mb)

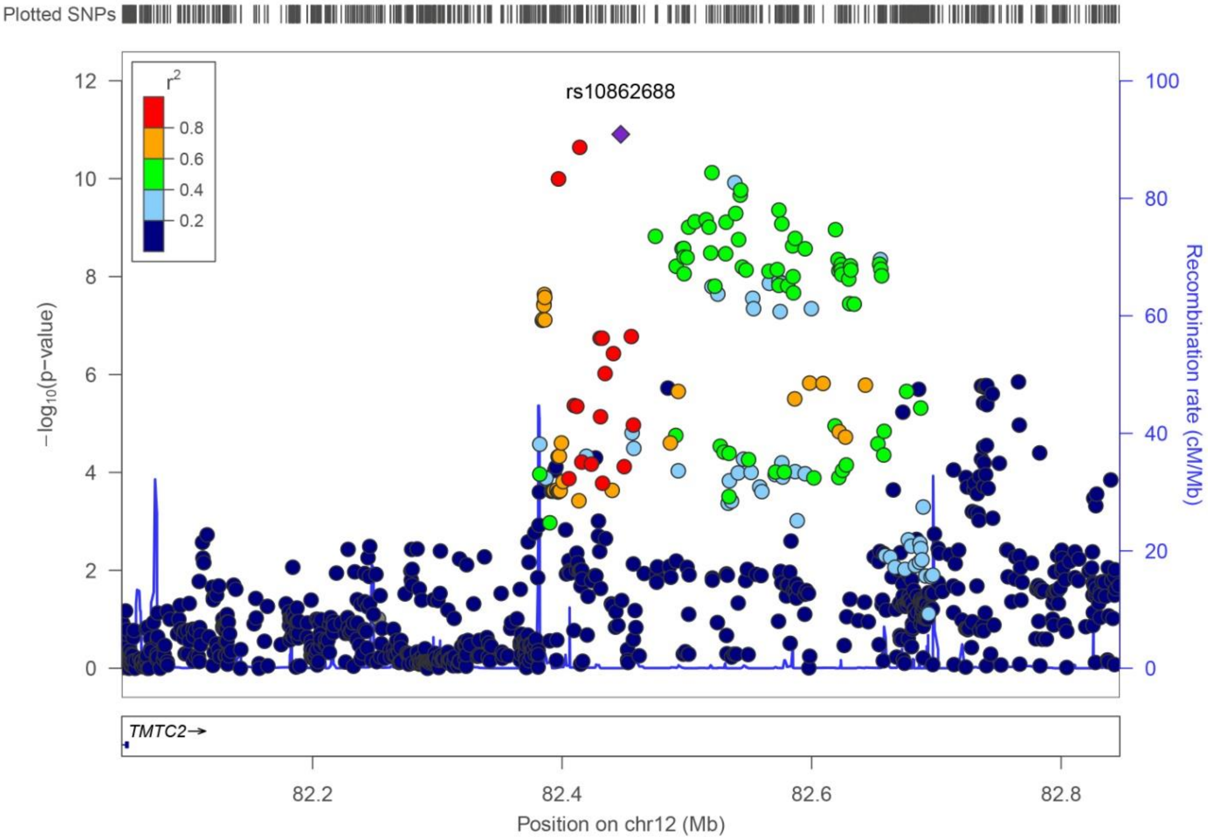
Plotted SNPs
rs10862688
r2
0.8
0.6
0.4
0.2
−log10(p−value)
Recombination rate (cM/Mb)
TMTC2
82.2
82.4
82.6
82.8
Position on chr12 (Mb)

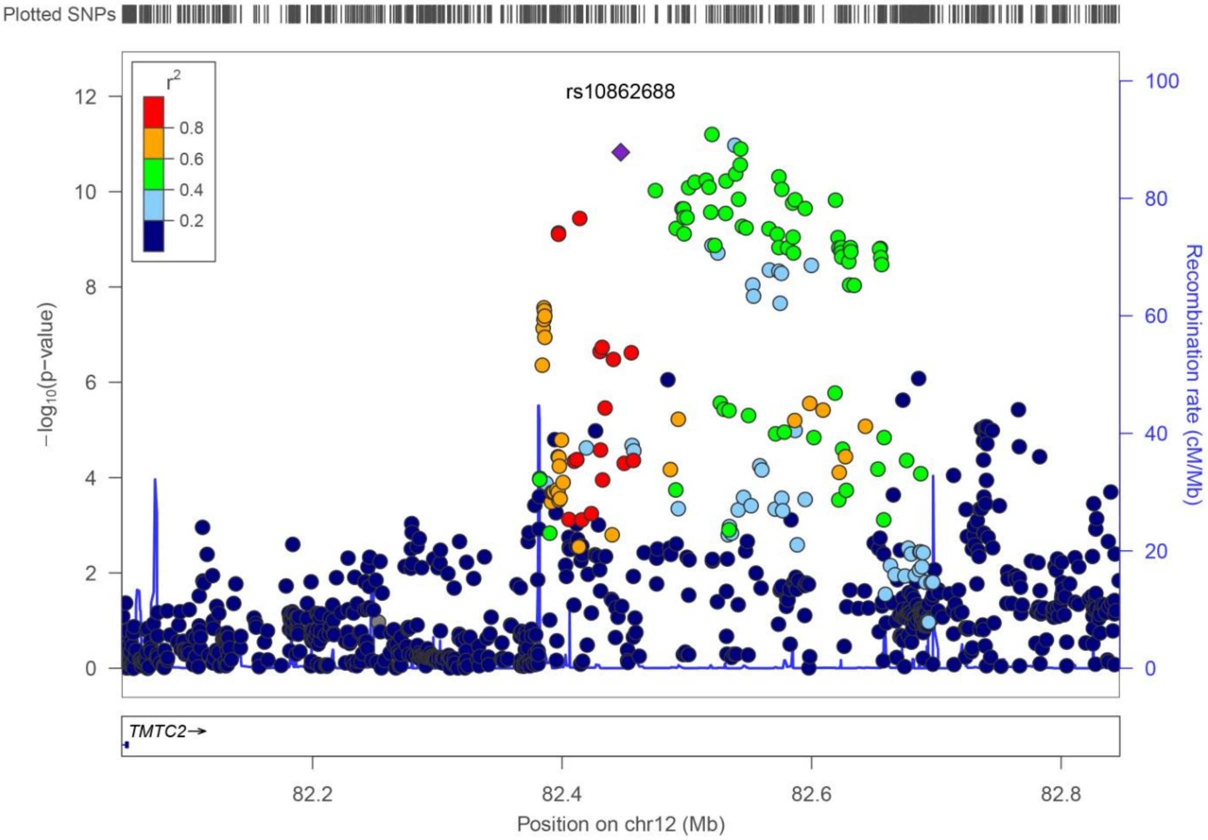
Plotted SNPs
rs10862688
r2
0.8
0.6
0.4
0.2
−log10(p−value)
Recombination rate (cM/Mb)
TMTC2
82.2
82.4
82.6
82.8
Position on chr12 (Mb)

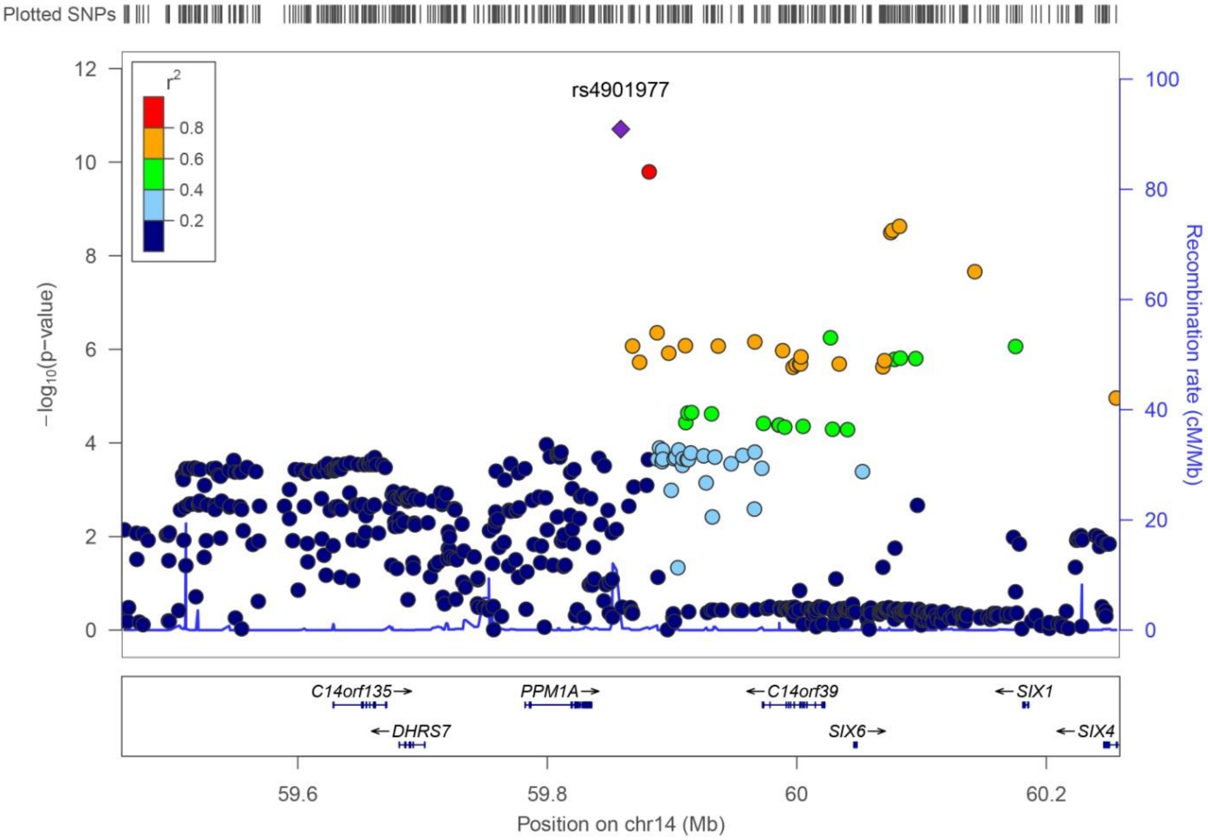

Plotted SNPs
rs4901977
r2
0.8
0.6
0.4
0.2
−log10(p−value)
Recombination rate (cM/Mb)
C14orf135→
PPM1A→
←C14orf39
←SIX1
←DHRS7
SIX6→
←SIX4
59.6
59.8
60
60.2
Position on chr14 (Mb)

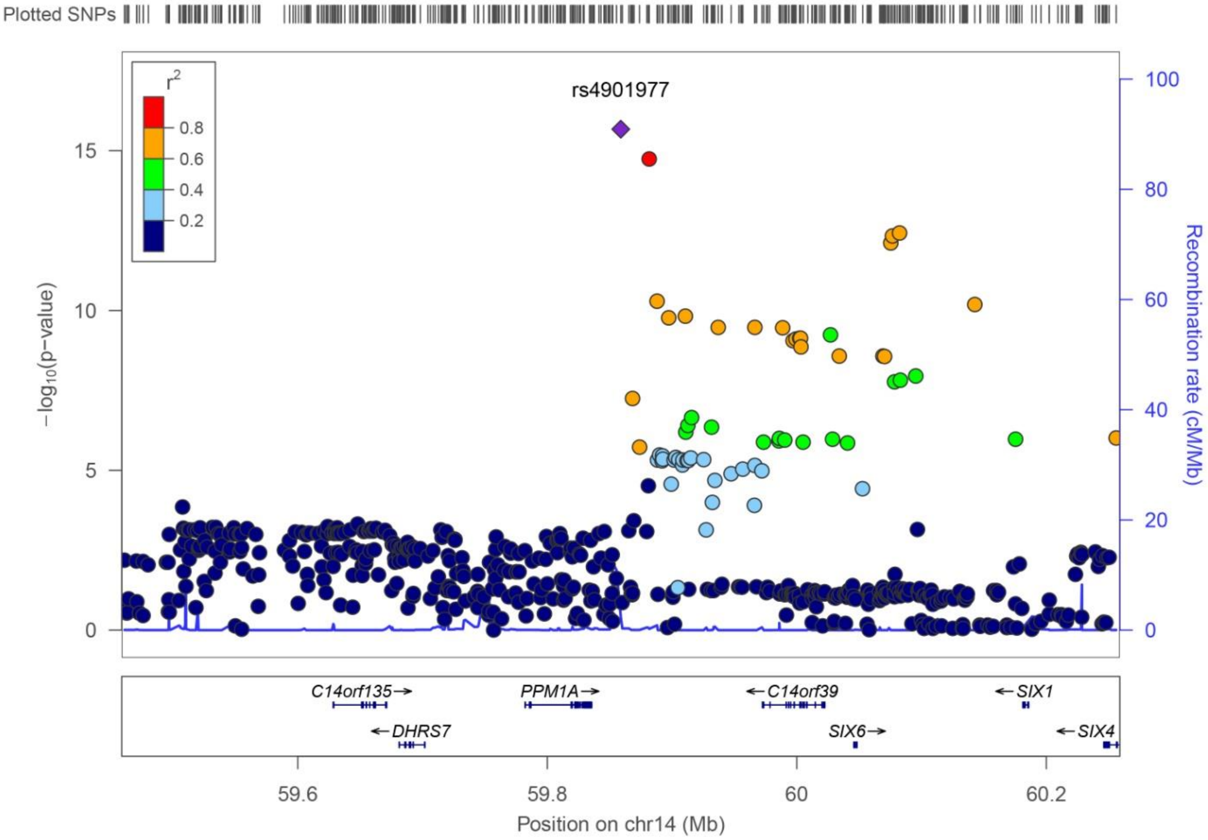

Plotted SNPs
rs4901977
r2
0.8
0.6
0.4
0.2
−log10(p−value)
Recombination rate (cM/Mb)
C14orf135→
PPM1A→
←C14orf39
←SIX1
←DHRS7
SIX6→
←SIX4
59.6
59.8
60
60.2
Position on chr14 (Mb)

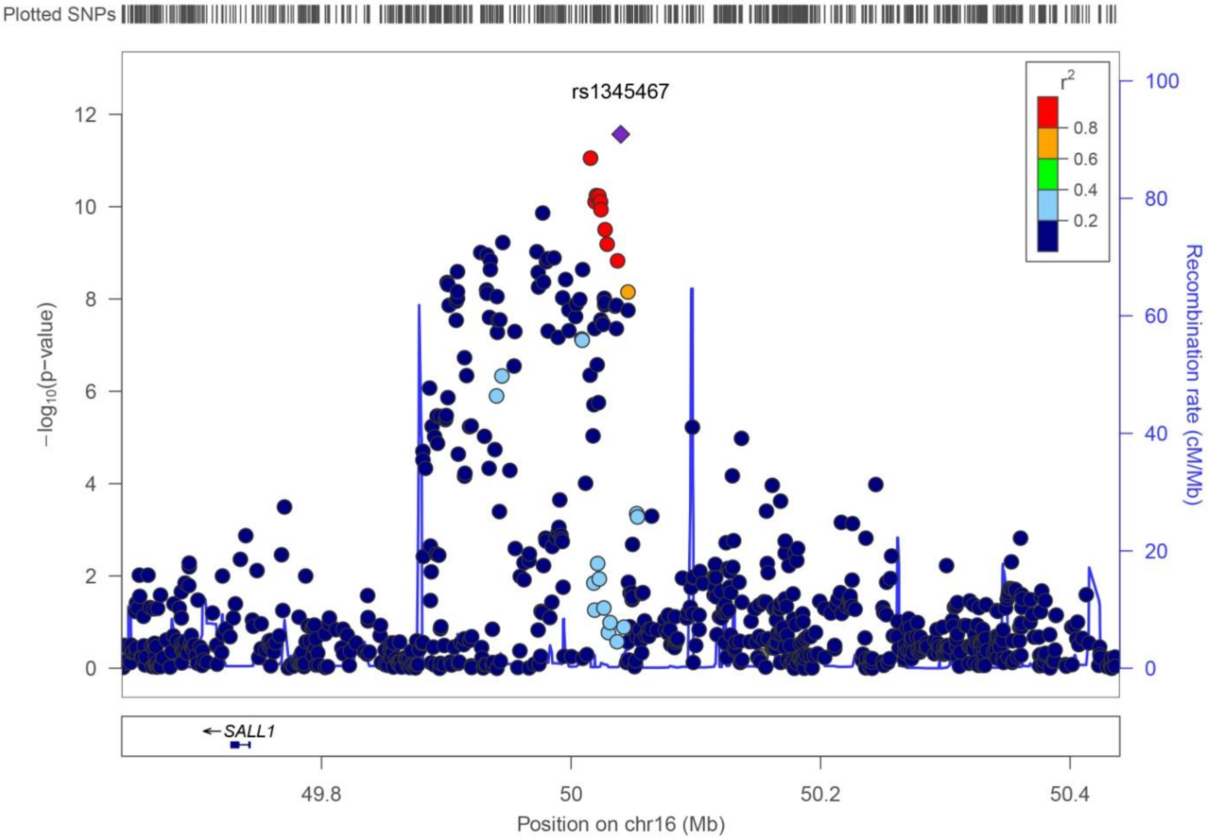
Plotted SNPs
rs1345467
r2
0.8
0.6
0.4
0.2
12
10
8
6
4
2
0
−log10(p−value)
100
80
60
40
20
0
Recombination rate (cM/Mb)
SALL1
49.8
50
50.2
50.4
Position on chr16 (Mb)

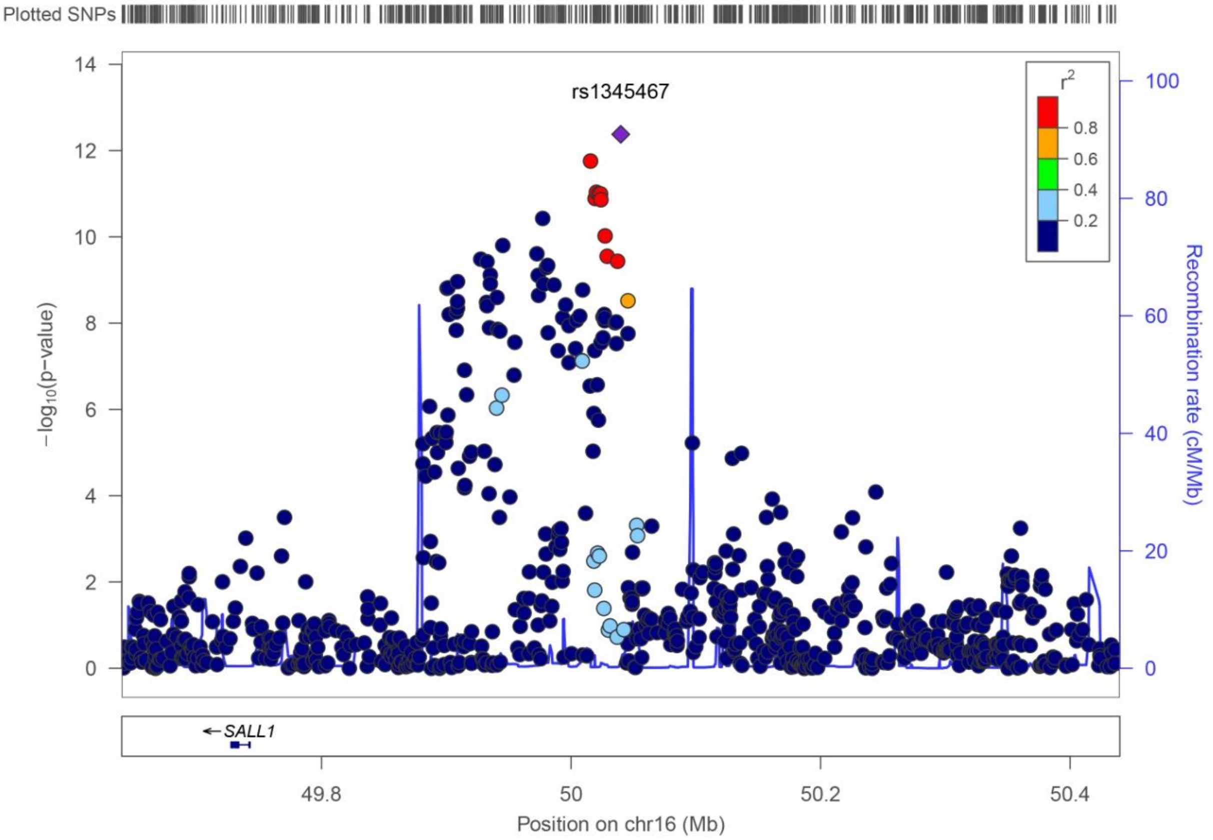
Plotted SNPs
rs1345467
r2
0.8
0.6
0.4
0.2
14
12
10
8
6
4
2
0
−log10(p−value)
100
80
60
40
20
0
Recombination rate (cM/Mb)
SALL1
49.8
50
50.2
50.4
Position on chr16 (Mb)

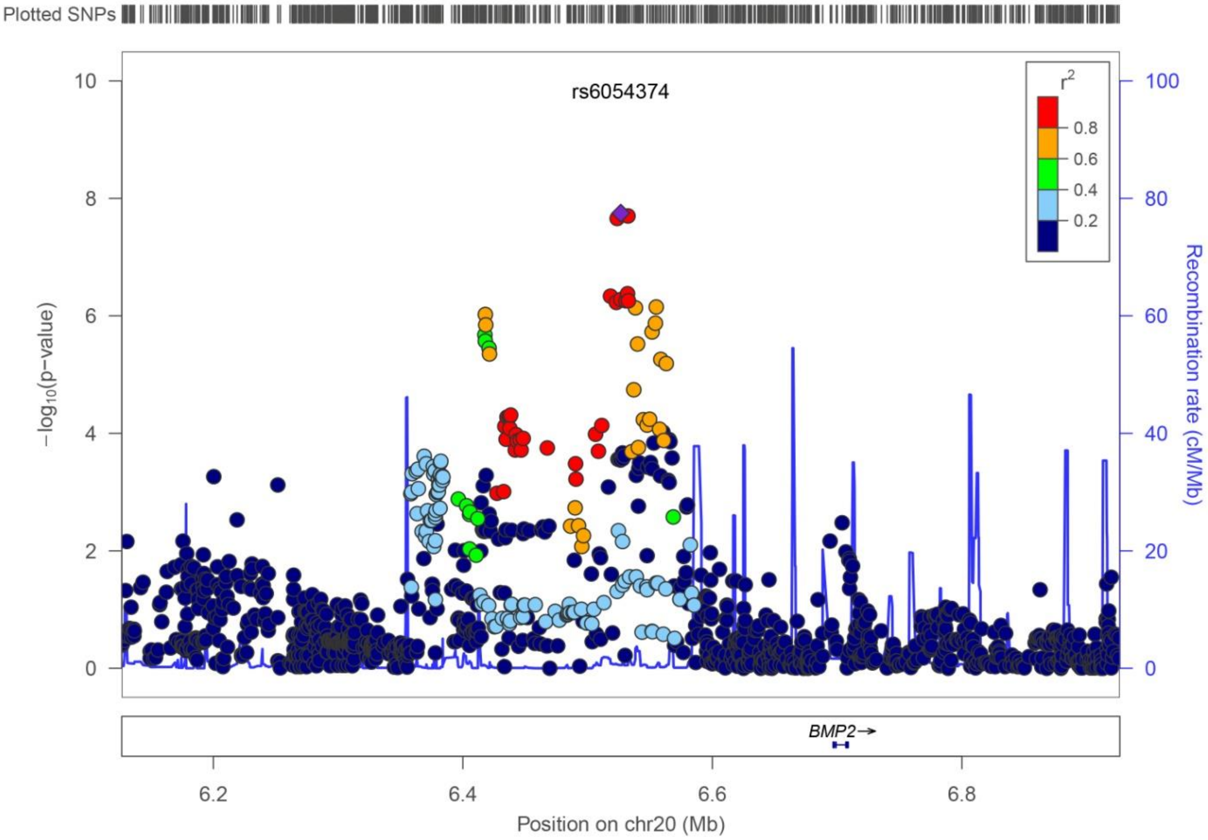
Plotted SNPs
rs6054374
r2
0.8
0.6
0.4
0.2
−log10(p−value)
Recombination rate (cM/Mb)
BMP2→
Position on chr20 (Mb)
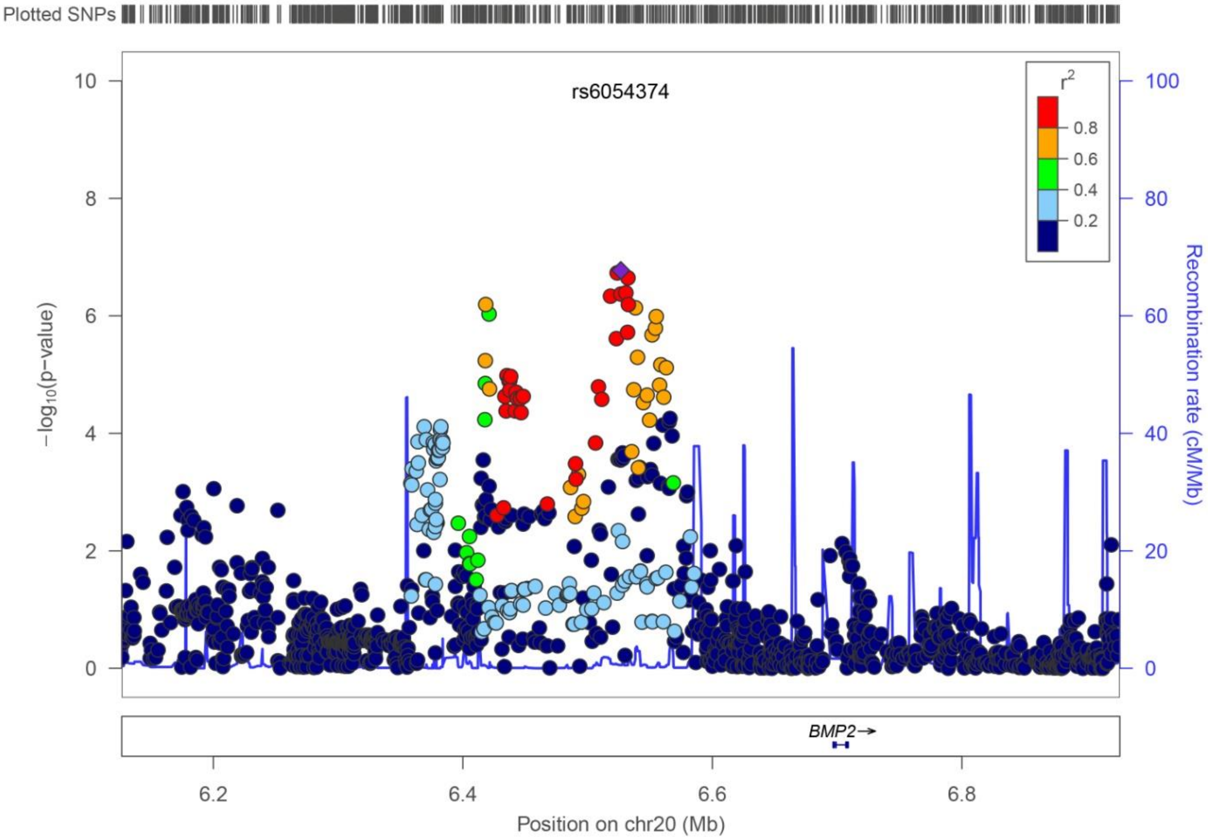
Plotted SNPs
rs6054374
r2
0.8
0.6
0.4
0.2
−log10(p−value)
Recombination rate (cM/Mb)
BMP2→
Position on chr20 (Mb)

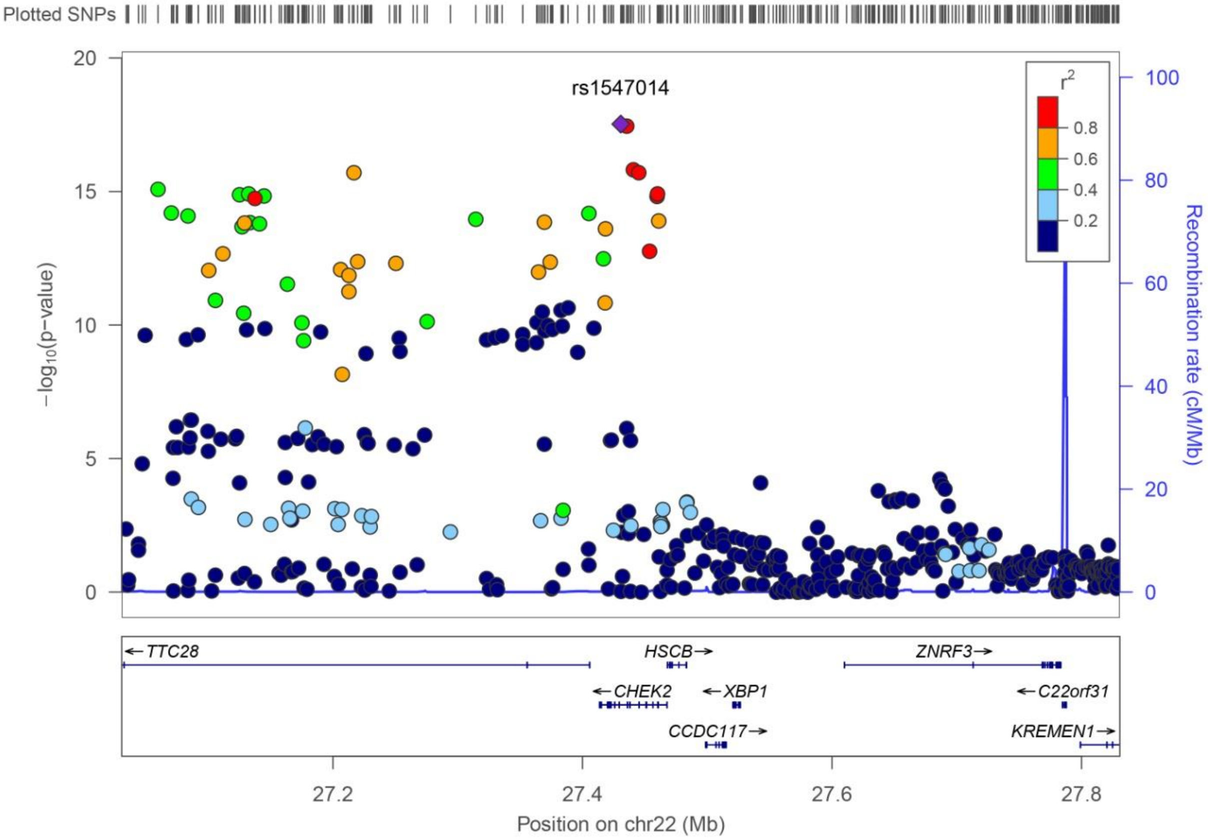
Plotted SNPs
rs1547014
−log10(p−value)
Recombination rate (cM/Mb)
r2
0.8
0.6
0.4
0.2
TTC28
HSCB
ZNRF3
CHEK2
XBP1
C22orf31
CCDC117
KREMEN1
Position on chr22 (Mb)

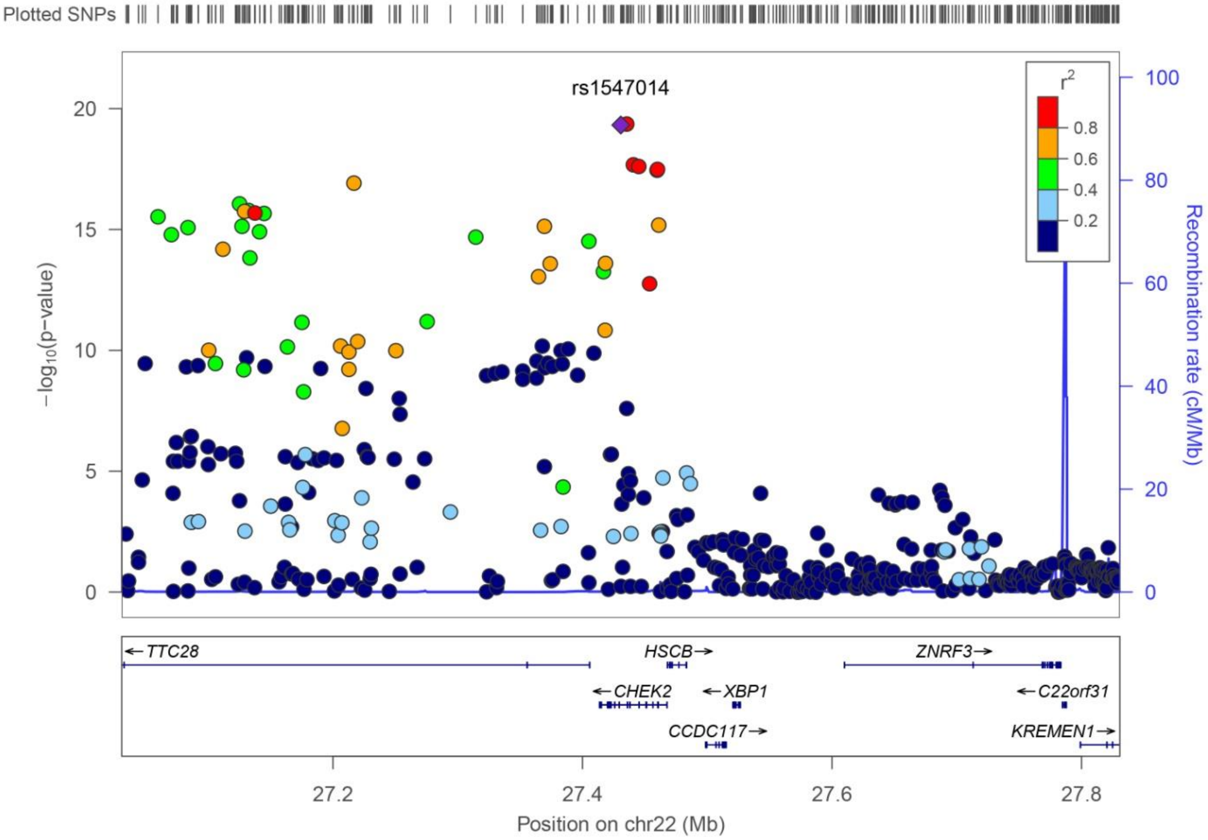
Plotted SNPs
rs1547014
−log10(p−value)
Recombination rate (cM/Mb)
r2
0.8
0.6
0.4
0.2
TTC28
HSCB
ZNRF3
CHEK2
XBP1
C22orf31
CCDC117
KREMEN1
Position on chr22 (Mb)

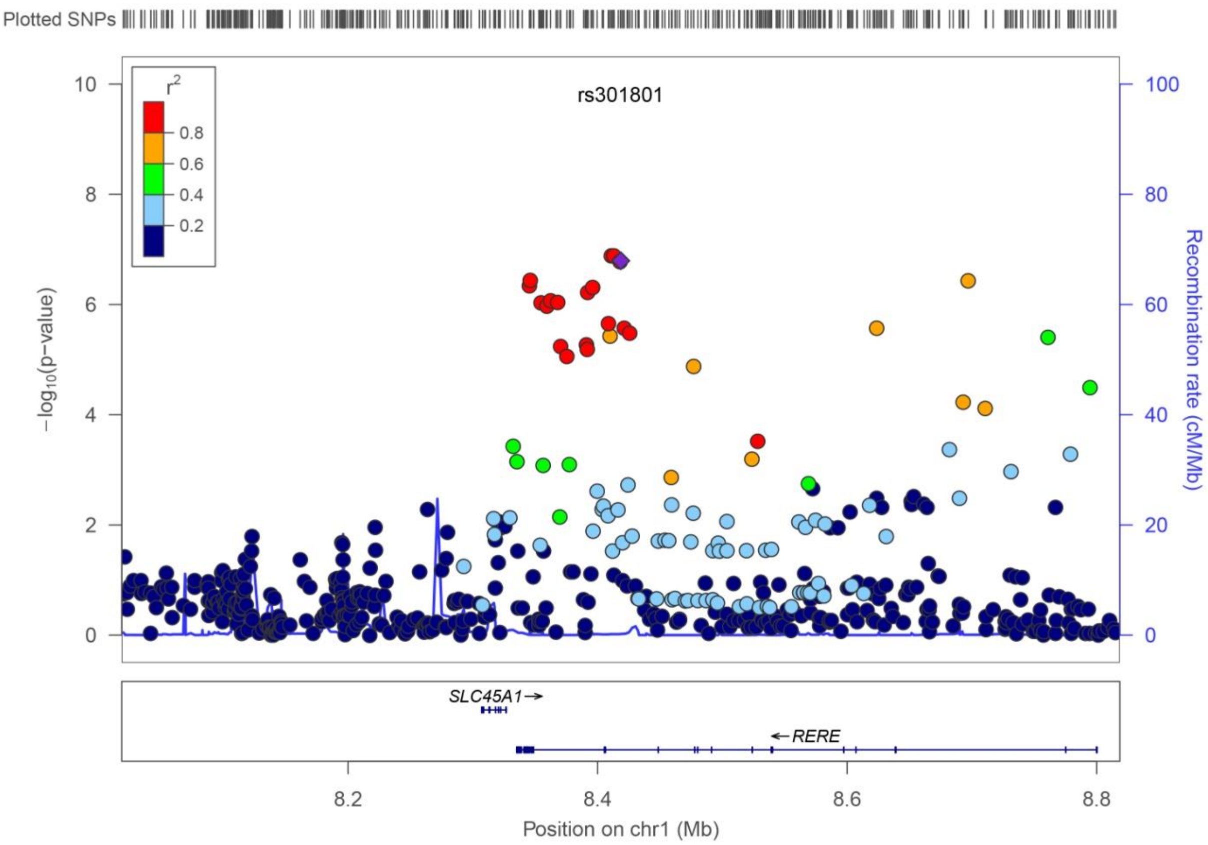
Plotted SNPs
rs301801
r²
0.8
0.6
0.4
0.2
−log10(p−value)
Recombination rate (cM/Mb)
SLC45A1→
←RERE
Position on chr1 (Mb)

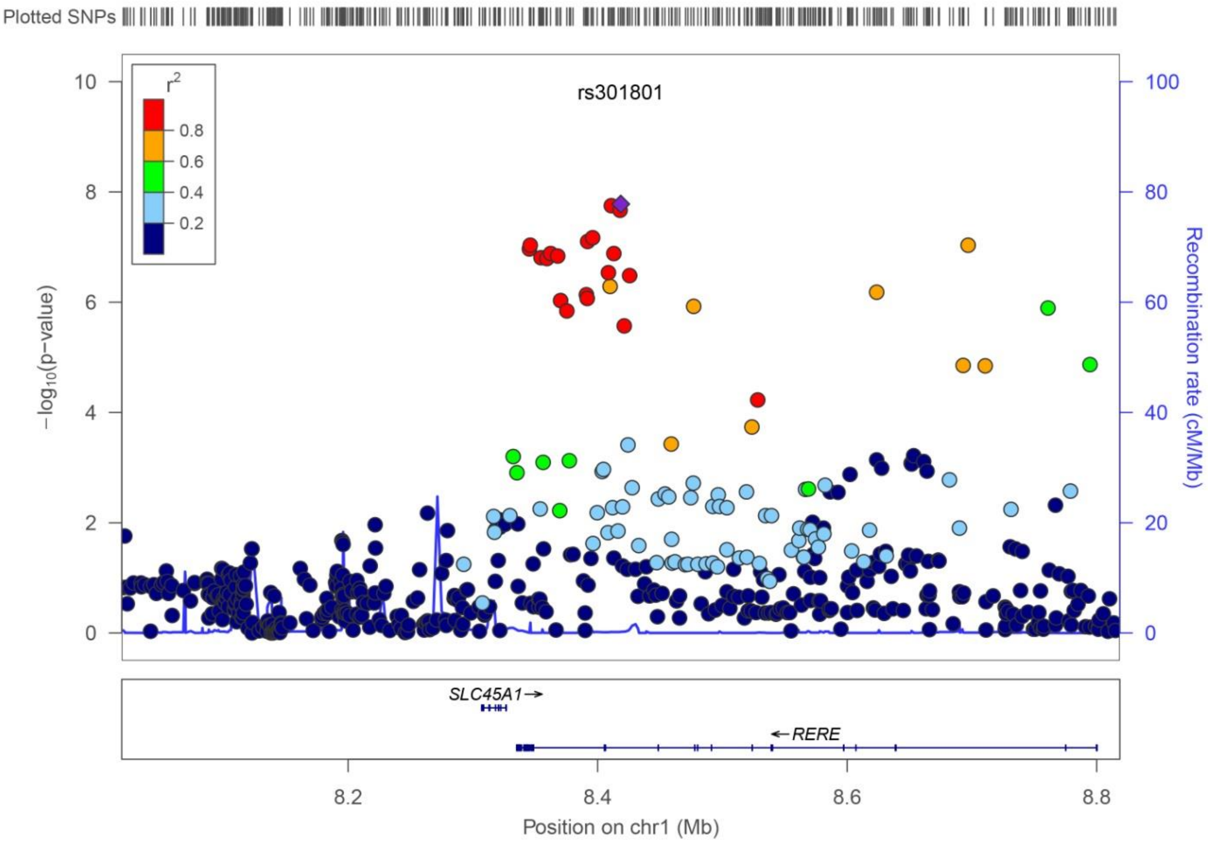
Plotted SNPs
rs301801
r²
0.8
0.6
0.4
0.2
−log10(p−value)
Recombination rate (cM/Mb)
SLC45A1→
←RERE
Position on chr1 (Mb)

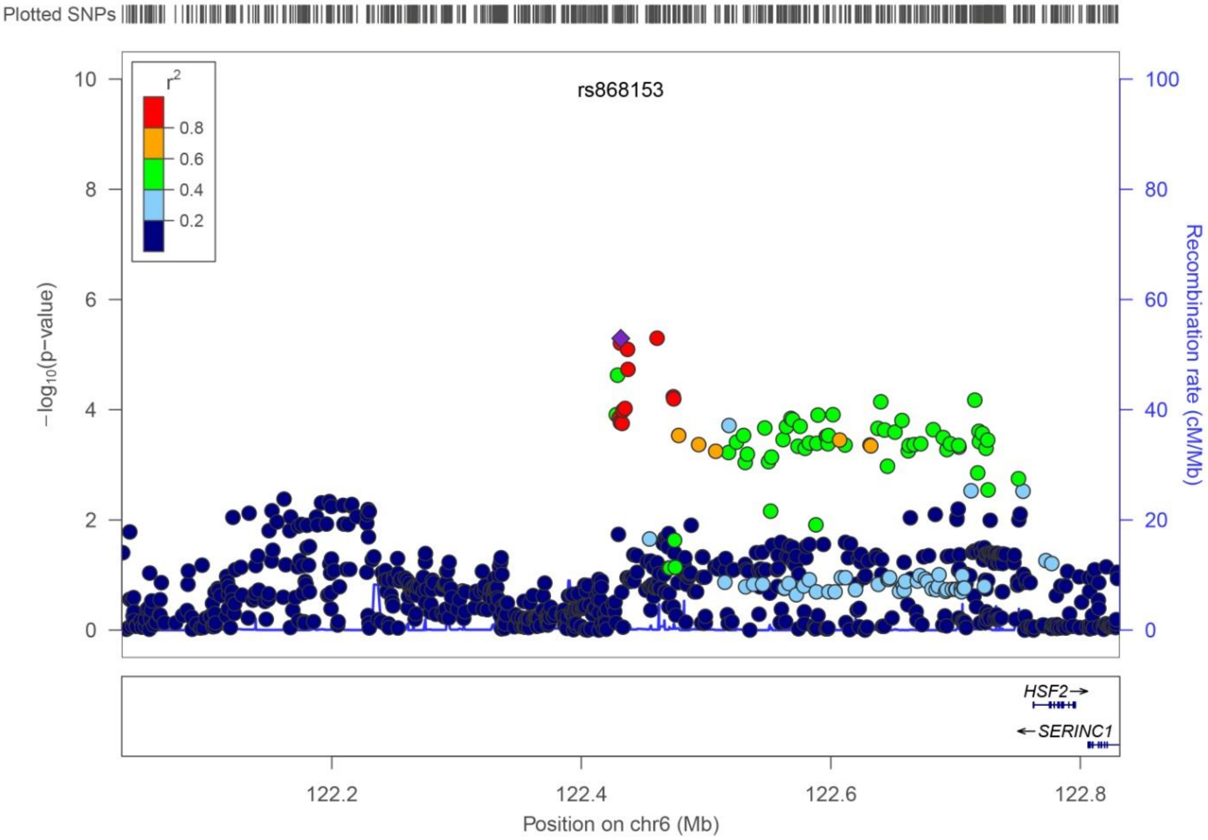
Plotted SNPs
rs868153
r²
0.8
0.6
0.4
0.2
−log10(p−value)
Recombination rate (cM/Mb)
HSF2→
←SERINC1
122.2
122.4
122.6
122.8
Position on chr6 (Mb)

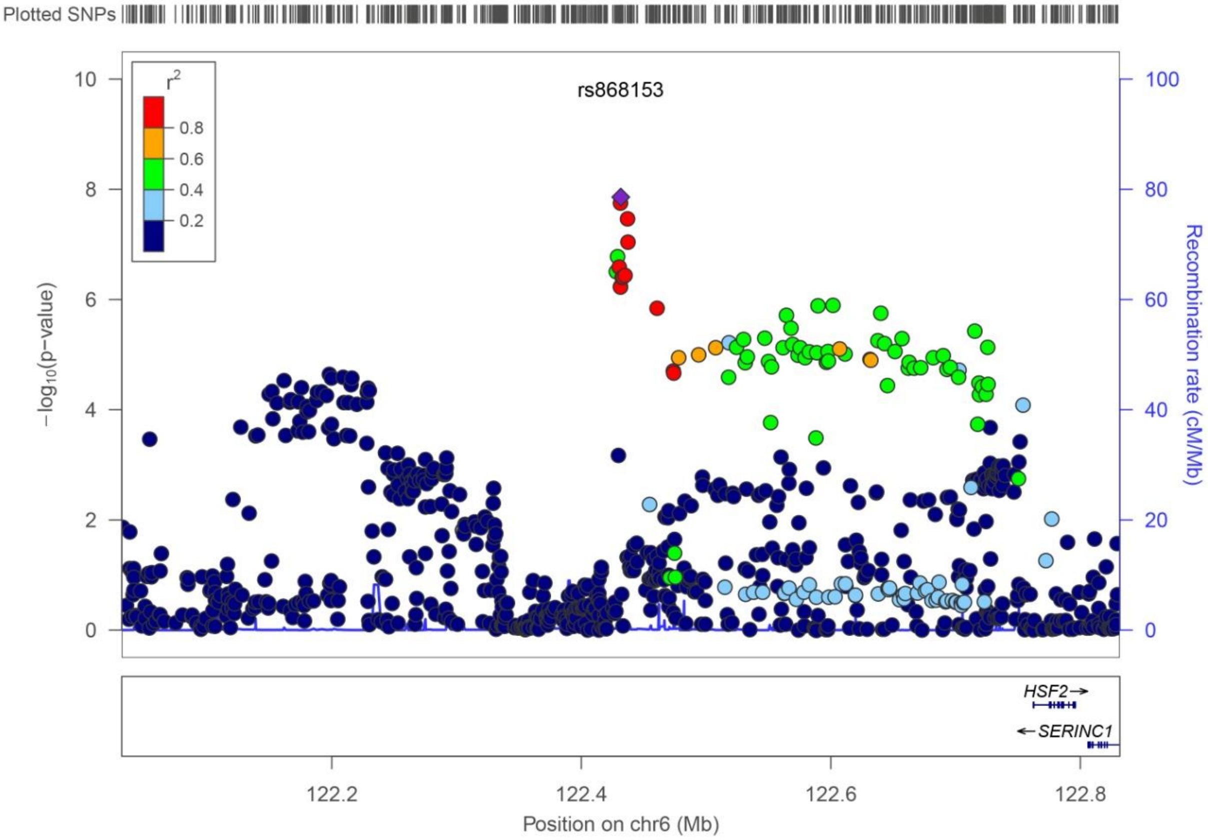
Plotted SNPs
rs868153
r²
0.8
0.6
0.4
0.2
−log10(p−value)
Recombination rate (cM/Mb)
HSF2→
←SERINC1
122.2
122.4
122.6
122.8
Position on chr6 (Mb)

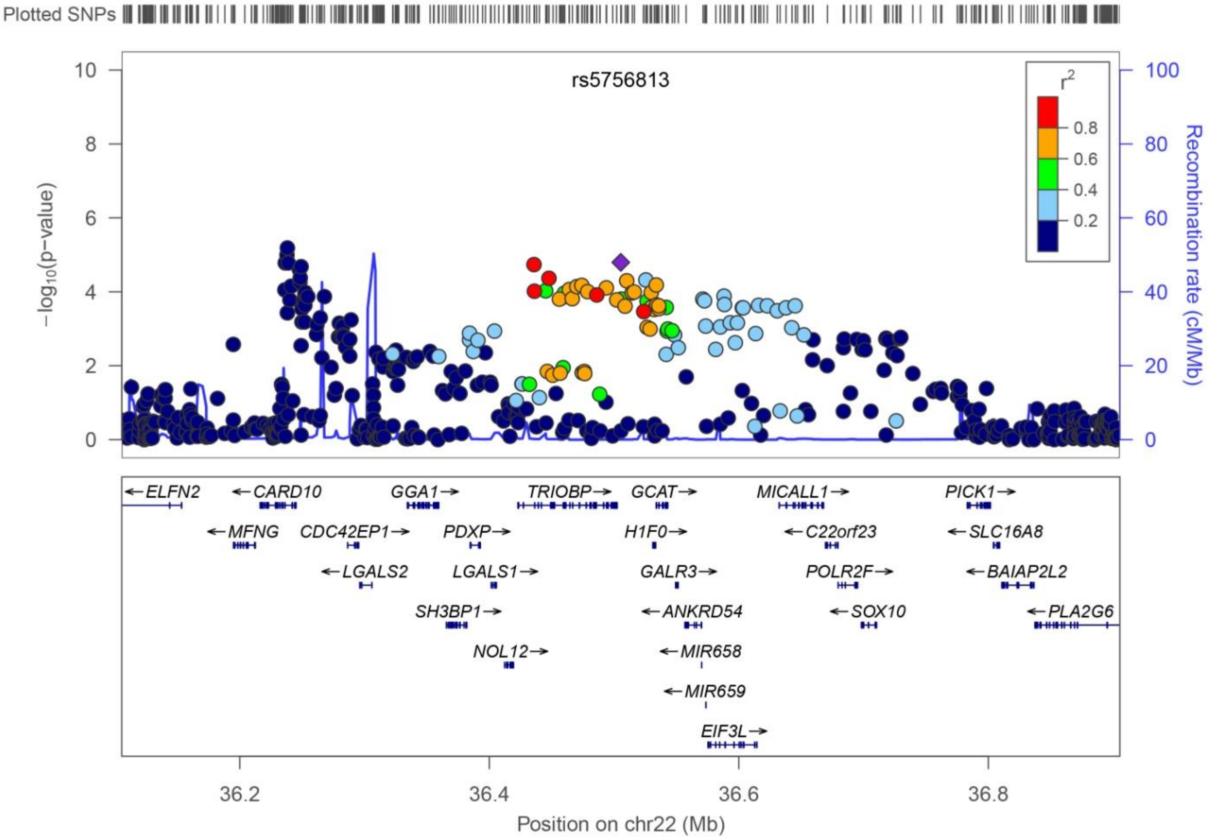

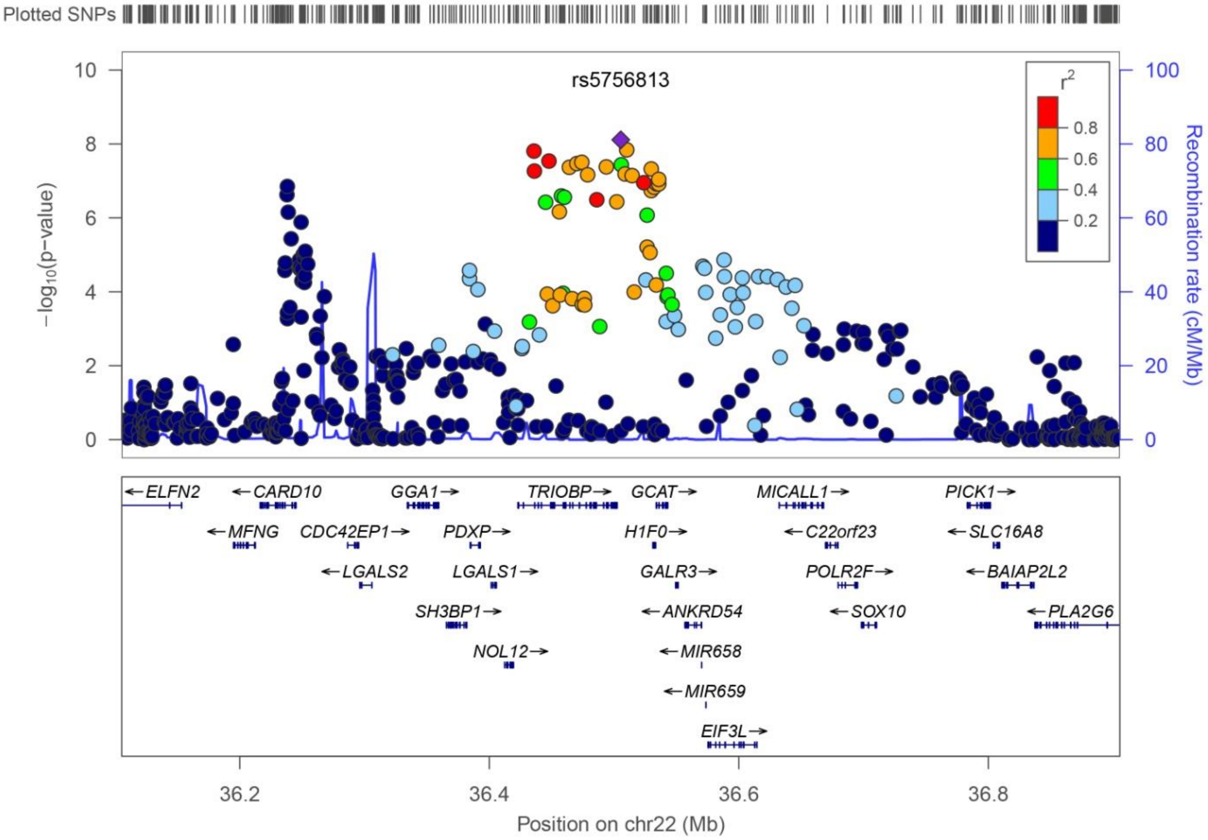

Upper figures represent the results from the meta-analysis of studies with European ancestry; lower figures represent the results from the meta-analysis of studies with European and Asian ancestry. Plots are centered on the most significant SNP at each locus and flanked by the meta-analysis results

for SNPs in the 400-kb region surrounding it. For each locus, the topSNP (lowest P value) is depicted as a purple diamond; other SNPs are shaded according to their pairwise correlation (R^2) with the topSNP. The blue line represents the estimated recombination rates; the gene annotations are shown below the figure. Plots were created with Locuszoom (Locuszoom (http://csg.sph.umich.edu/locuszoom). Figures are shown in the same order as in main Table 1.

Supplementary Figure 6. Forest plots for all 18 identified VCDR loci

For each study, the • shows the beta linear regression coefficient or the average difference in VCDR for each additional copy of the minor allele and the lines represent the standard error of the estimate. Figures are shown in the same order as in main table 1.

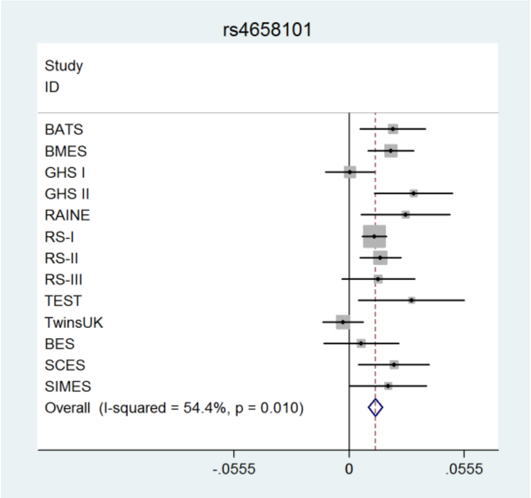

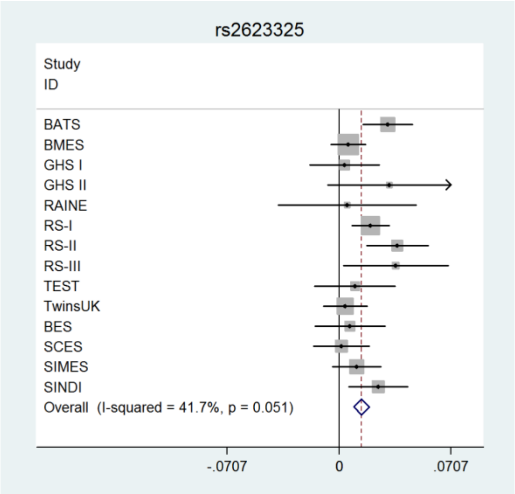

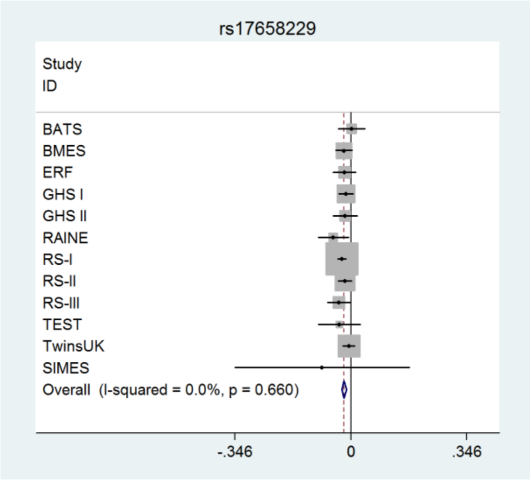

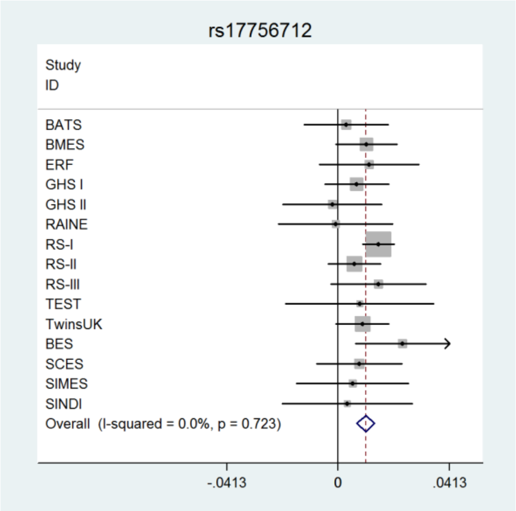

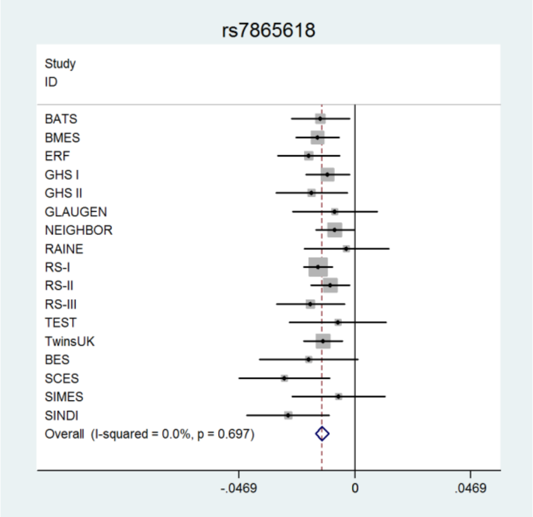

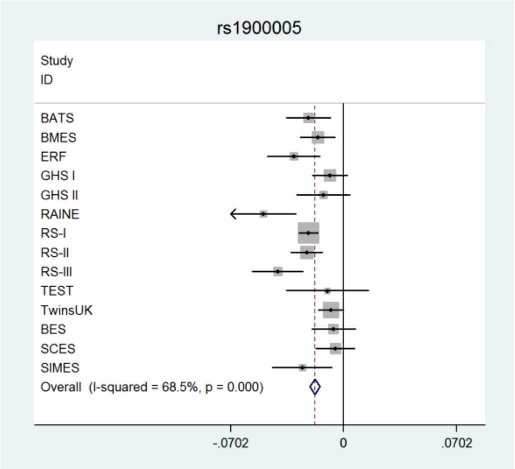

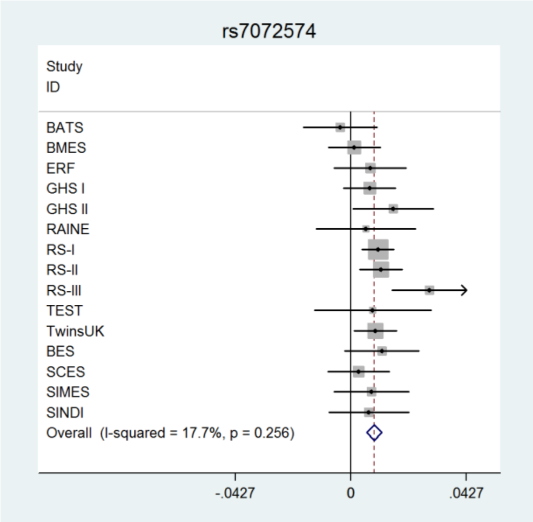
rs7072574
Study
ID
BATS
BMES
ERF
GHS I
GHS II
RAINE
RS-I
RS-II
RS-III
TEST
TwinsUK
BES
SCES
SIMES
SINDI
Overall (I-squared = 17.7%, p = 0.256)
-.0427
0
.0427

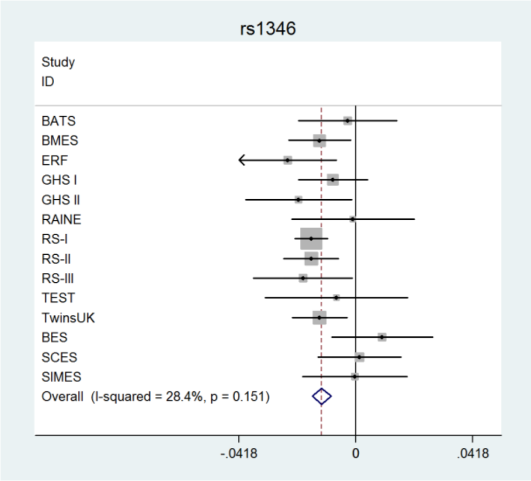
rs1346
Study
ID
BATS
BMES
ERF
GHS I
GHS II
RAINE
RS-I
RS-II
RS-III
TEST
TwinsUK
BES
SCES
SIMES
Overall (I-squared = 28.4%, p = 0.151)
-.0418
0
.0418

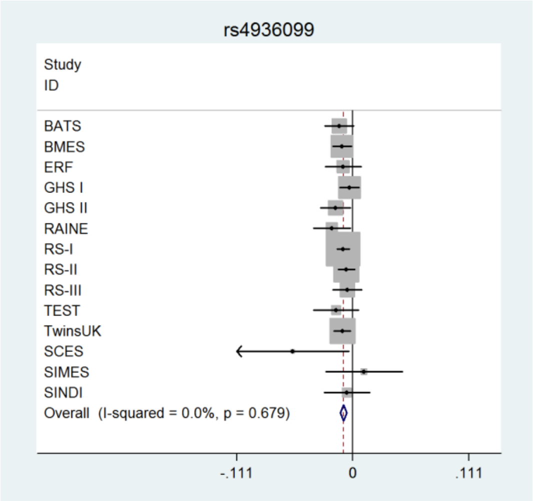
rs4936099
Study
ID
BATS
BMES
ERF
GHS I
GHS II
RAINE
RS-I
RS-II
RS-III
TEST
TwinsUK
SCES
SIMES
SINDI
Overall (I-squared = 0.0%, p = 0.679)
-.111
0
.111

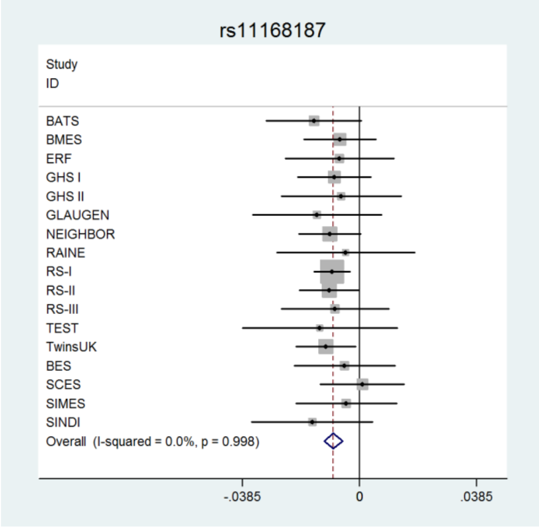
rs11168187
Study
ID
BATS
BMES
ERF
GHS I
GHS II
GLAUGEN
NEIGHBOR
RAINE
RS-I
RS-II
RS-III
TEST
TwinsUK
BES
SCES
SIMES
SINDI
Overall (I-squared = 0.0%, p = 0.998)
-.0385
0
.0385

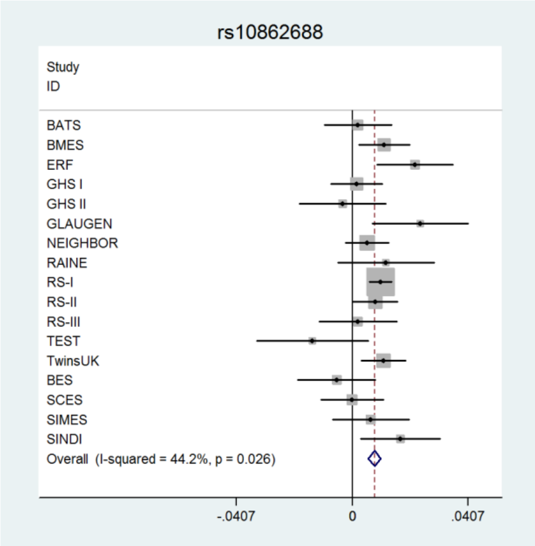
rs10862688
Study
ID
BATS
BMES
ERF
GHS I
GHS II
GLAUGEN
NEIGHBOR
RAINE
RS-I
RS-II
RS-III
TEST
TwinsUK
BES
SCES
SIMES
SINDI
Overall (I-squared = 44.2%, p = 0.026)
-.0407
0
.0407

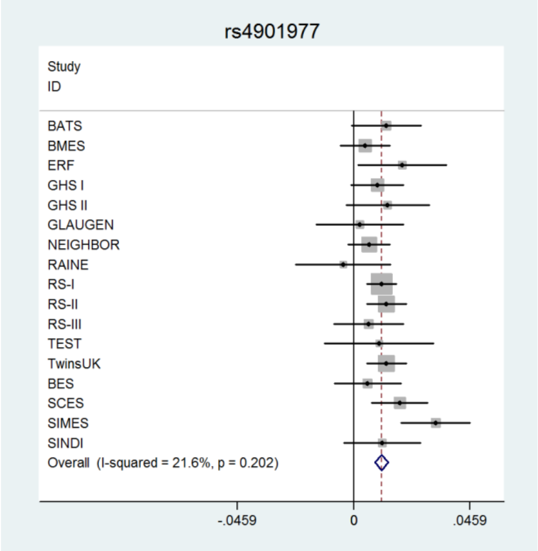
rs4901977
Study
ID
BATS
BMES
ERF
GHS I
GHS II
GLAUGEN
NEIGHBOR
RAINE
RS-I
RS-II
RS-III
TEST
TwinsUK
BES
SCES
SIMES
SINDI
Overall (I-squared = 21.6%, p = 0.202)
-.0459
0
.0459

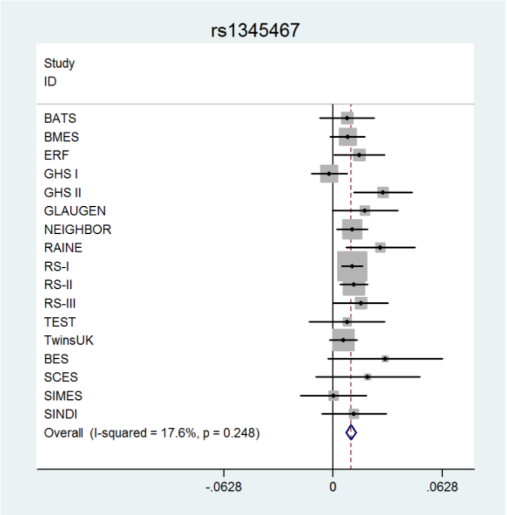
rs1345467
Study ID
BATS
BMES
ERF
GHS I
GHS II
GLAUGEN
NEIGHBOR
RAINE
RS-I
RS-II
RS-III
TEST
TwinsUK
BES
SCES
SIMES
SINDI
Overall (I-squared = 17.6%, p = 0.248)
-.0628
0
.0628

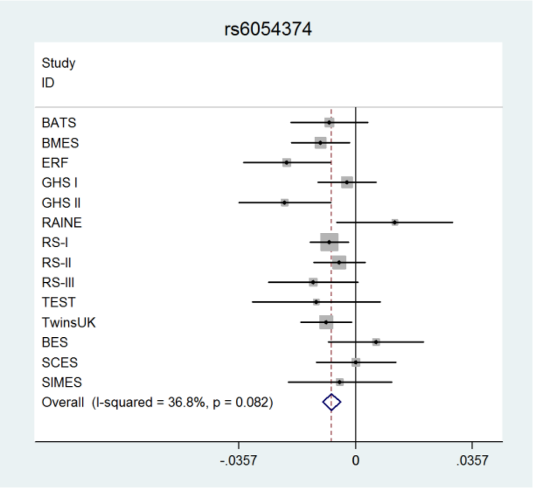
rs6054374
Study ID
BATS
BMES
ERF
GHS I
GHS II
RAINE
RS-I
RS-II
RS-III
TEST
TwinsUK
BES
SCES
SIMES
Overall (I-squared = 36.8%, p = 0.082)
-.0357
0
.0357

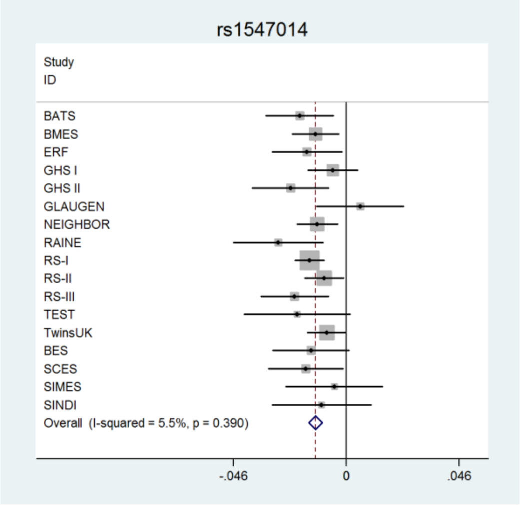
rs1547014
Study ID
BATS
BMES
ERF
GHS I
GHS II
GLAUGEN
NEIGHBOR
RAINE
RS-I
RS-II
RS-III
TEST
TwinsUK
BES
SCES
SIMES
SINDI
Overall (I-squared = 5.5%, p = 0.390)
-.046
0
.046

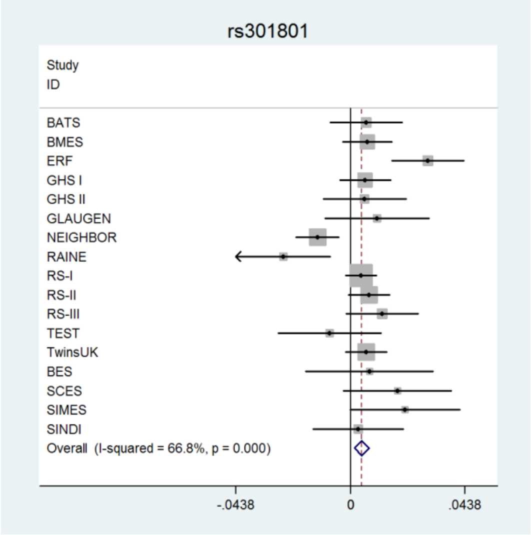
rs301801
Study ID
BATS
BMES
ERF
GHS I
GHS II
GLAUGEN
NEIGHBOR
RAINE
RS-I
RS-II
RS-III
TEST
TwinsUK
BES
SCES
SIMES
SINDI
Overall (I-squared = 66.8%, p = 0.000)
-.0438
0
.0438

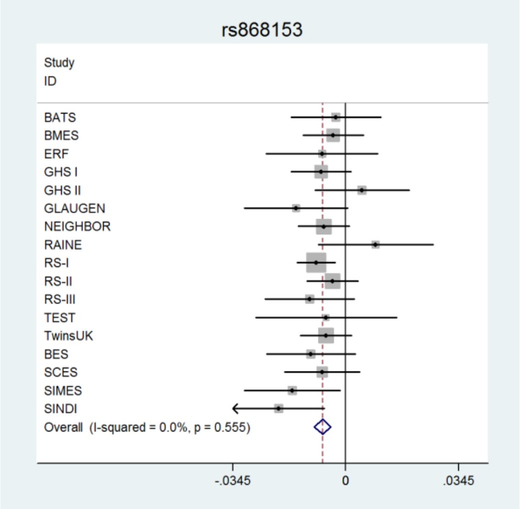
rs868153
Study ID
BATS
BMES
ERF
GHS I
GHS II
GLAUGEN
NEIGHBOR
RAINE
RS-I
RS-II
RS-III
TEST
TwinsUK
BES
SCES
SIMES
SINDI
Overall (I-squared = 0.0%, p = 0.555)
-.0345
0
.0345

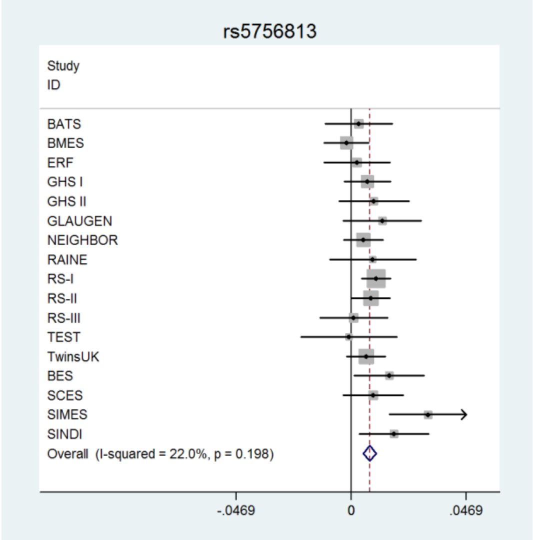
rs5756813
Study ID
BATS
BMES
ERF
GHS I
GHS II
GLAUGEN
NEIGHBOR
RAINE
RS-I
RS-II
RS-III
TEST
TwinsUK
BES
SCES
SIMES
SINDI
Overall (I-squared = 22.0%, p = 0.198)
-.0469
0
.0469

Supplementary Figure 7. Region plots for gene-based tests

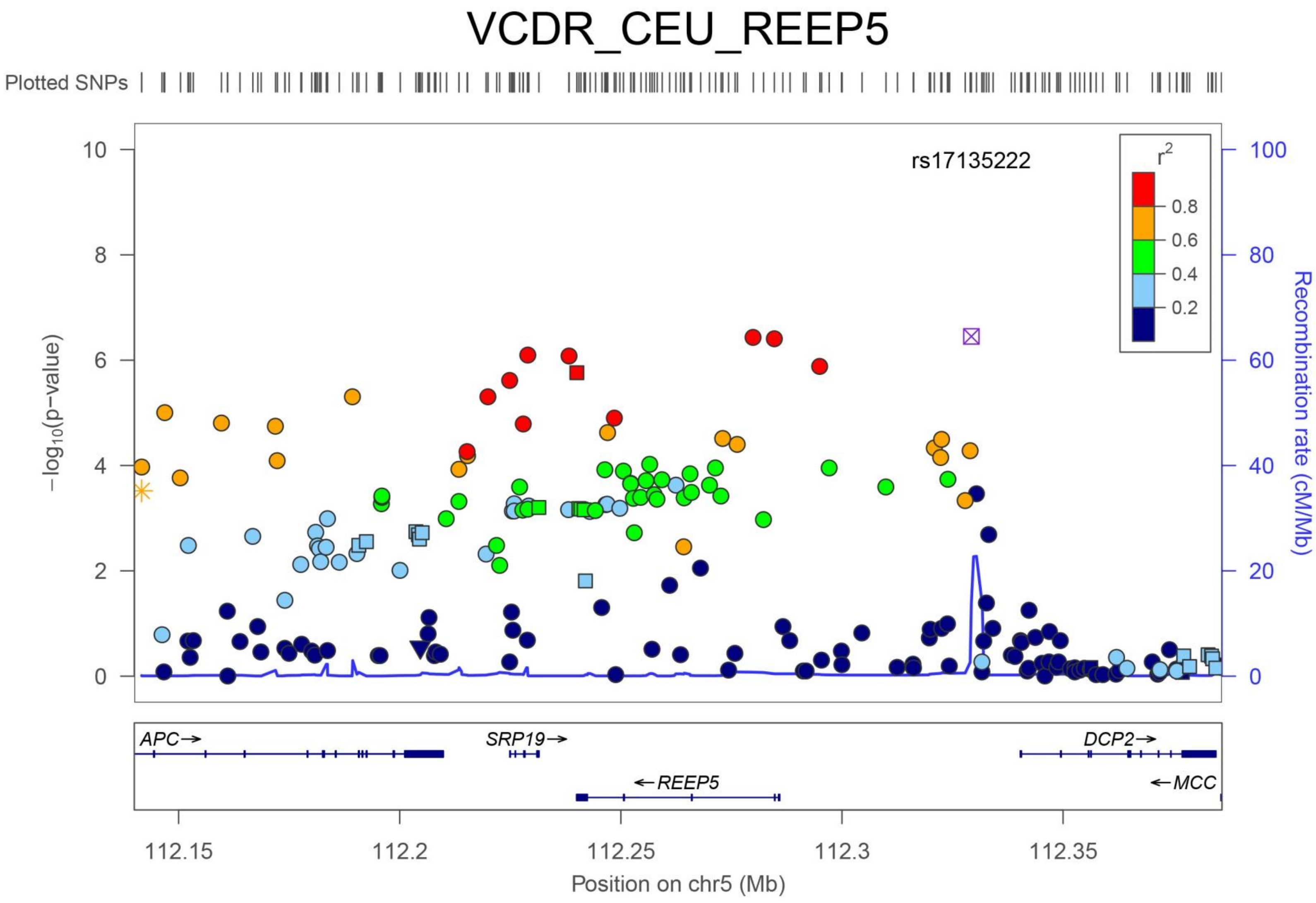

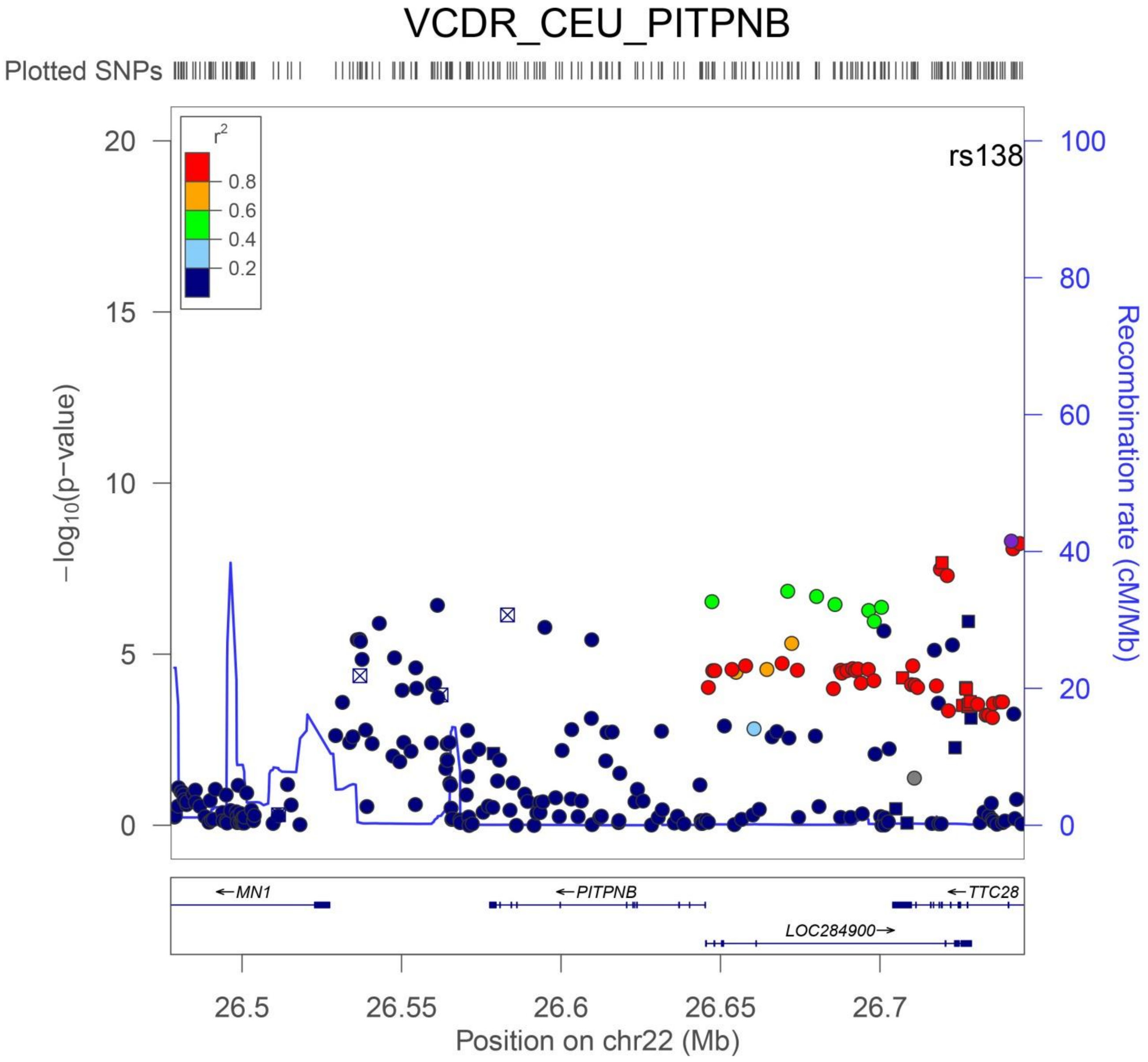
VCDR_CEU_PITPNB
Plotted SNPs
r²
0.8
0.6
0.4
0.2
rs138
20
15
10
5
0
100
80
60
40
20
0
−log10(p−value)
Recombination rate (cM/Mb)
MN1
PITPNB
TTC28
LOC284900
26.5
26.55
26.6
26.65
26.7
Position on chr22 (Mb)

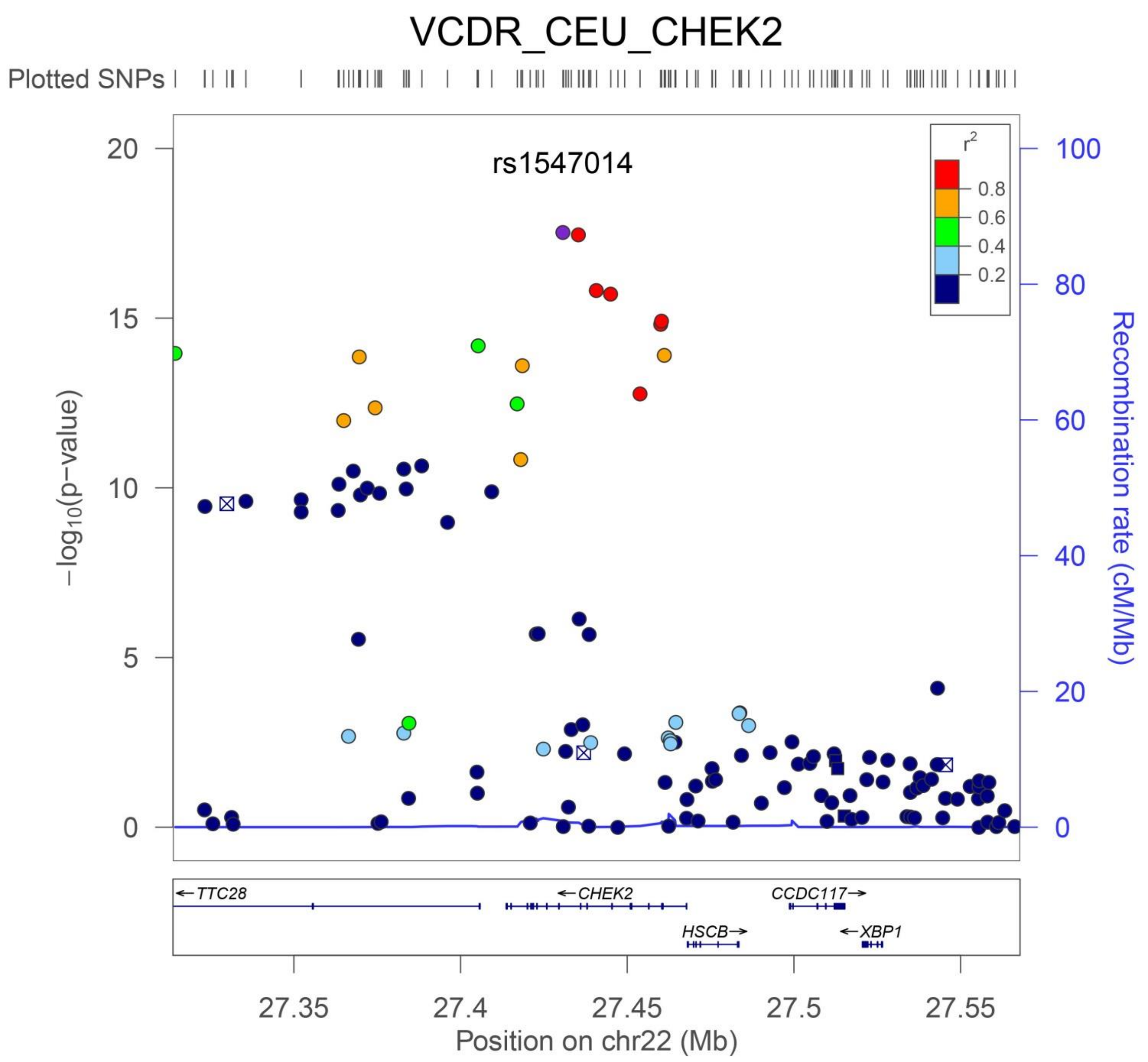

VCDR_CEU_CHEK2
Plotted SNPs
rs1547014
r2
0.8
0.6
0.4
0.2
20
15
10
5
0
−log10(p−value)
100
80
60
40
20
0
Recombination rate (cM/Mb)
←TTC28
←CHEK2
CCDC117→
HSCB→
←XBP1
27.35
27.4
27.45
27.5
27.55
Position on chr22 (Mb)

Supplementary Table 1. Study descriptives

Study	n	Mean age (sd)	Age range	% men	Mean VCDR (sd)	VCDR range	R
BATS	966	20.2 (3.8)	13-34	46.1	0.46 (0.13)	0.09-0.75	0.83
BMES	1656	66.2 (9.8)	49-97	57.0	0.43 (0.13)	0.09-0.94	0.71
ERF	2131	47.3 (14.0)	18-85	44.4	0.31 (0.20)	0.00-0.87	0.71
GHS I	783	55.9 (10.9)	35-74	52.7	0.42 (0.09)	0.22-0.86	0.46
GHS II	485	55.1 (10.9)	35-74	49.1	0.42 (0.11)	0.17-0.90	0.58
Glaugen (controls only)	341	64.4 (11.1)	40-89	46.3	0.30 (0.10)	0.10-0.70	0.85
Neighbor (controls only)	2050	69.3 (11.3)	35-97	43.9	0.32 (0.13)	0.00-0.90	0.90
RAINE	1046	20.0 (0.4)	18-22	51.3	0.28 (0.20)	0.00-0.78	0.77
RS-I	5322	68.0 (8.4)	55-99	41.6	0.50 (0.13)	0.05-0.87	0.71
RS-II	2054	64.9 (7.8)	55-98	45.8	0.50 (0.13)	0.10-0.86	0.71
RS-III	1962	56.1 (5.5)	45-89	43.9	0.29 (0.21)	0.00-1.00	0.73
TEST	376	20.9 (17.4)	5-79	41.5	0.44 (0.12)	0.09-0.88	0.80
TwinsUK	1922	56.9 (11.6)	16-83	2.1	0.34 (0.11)	0.04-0.70	0.80
BES	727	58.6 (9.3)	45-86	35.0	0.43 (0.13)	0.00-0.77	NA
SCES	1726	58.8 (9.6)	44-86	51.2	0.34 (0.17)	0.00-0.95	0.64
SINDI	2026	58.0 (10.0)	43-84	51.2	0.46 (0.23)	0.00-1.00	0.67
SIMES	2305	59.1 (11.0)	40-80	49.4	0.39 (0.23)	0.00-1.00	0.60

R = Pearson correlation coefficient between right and left eye for the vertical cup-disc ratio (this could not be calculated for the Beijing Eye Study because they only measured the right eye); sd = standard deviation; VCDR = vertical cup-disc ratio

Supplementary Table 2. Study specific lambda (λ) estimates

Study	λ
BATS	1.015
BMES	1.006
ERF	1.060
GHS I	0.999
GHS II	0.998
Glaugen	0.992
Neighbor	0.997
RAINE	1.002
RS-I	0.977
RS-II	1.121
RS-III	1.077
TEST	1.000
TwinsUK	1.060
BES	1.040
SCES	1.005
SINDI	1.026
SIMES	1.030

Supplementary Table 3. The most significant SNP in Asians within 100,000 basepairs from the most significant associated SNP in Europeans

Chr.	Gene	European ancestry			Asian ancestry			Combined (European + Asian ancestry)		
		rs number	basepair	P value	rs number	basepair	P value	rs number	basepair	P value
1	*CDC7/TGFBR3*	rs4658101	91849997	8.80E-14	rs1192419	91852647	3.29E-05	rs1192419	91852647	3.23E-17
3	*COL8A1*	rs2623325	100614445	7.05E-09	rs2700667	100568496	3.40E-03	rs2623325	100614445	6.61E-10
5	*DUSP1*	rs17658229	172123657	8.06E-09	rs1010483	172155569	3.19E-03	rs17658229	172123657	8.06E-09
6	*EXOC2*	rs17756712	570071	1.98E-08	rs708038	521902	4.48E-04	rs17756712	570071	1.13E-09
9	*CDKN2BAS*	rs7865618	22021005	2.80E-20	rs8181050	22054391	6.29E-07	rs7030641	22044040	2.86E-24
10	*ATOH7*	rs1900005	69668061	7.21E-31	rs7916697	69661859	1.31E-04	rs7916697	69661859	5.56E-32
10	*PLCE1*	rs7072574	96026296	6.17E-09	rs2077218	96061551	1.79E-03	rs7081879	96028033	5.36E-10
11	*SSSCA1*	rs1346	65093827	2.54E-15	rs1152620	65013905	4.62E-02	rs17146964	65005721	2.14E-13
11	*ADAMTS8*	rs4936099	129785935	6.38E-09	rs12416827	129841878	2.16E-03	rs4936099	129785935	4.61E-09
12	*RPAP3*	rs11168187	46330278	2.96E-08	rs1232987	46270481	6.11E-02	rs11168187	46330278	2.96E-08
12	*TMTC2*	rs10862688	82447043	1.24E-11	rs904091	82474799	1.77E-02	rs2645975	82520258	6.25E-12
14	*SIX1/6*	rs4901977	59858929	1.98E-11	rs8015152	59881752	9.79E-08	rs4901977	59858929	2.13E-16
16	*SALL1*	rs1345467	50039822	2.70E-12	rs1420985	49945338	1.63E-02	rs1345467	50039822	4.19E-13
20	*BMP2*	rs6054374	6526556	1.79E-08	rs1407033	6492657	5.97E-04	rs6054374	6526556	1.69E-07
22	*CHEK2*	rs1547014	27430711	2.98E-18	rs738722	27460012	4.60E-04	rs5752773	27435415	4.36E-20
1	*RERE*	rs301798	8411152	1.30E-07	rs301814	8433164	8.87E-04	rs301801	8418532	5.23E-02
6	*HSF2*	rs17084191	122460780	5.01E-06	rs1402538	122430550	5.09E-05	rs868153	122431654	7.96E-01
22	*CARD10*	rs5756813	36505423	1.60E-05	rs2413485	36523866	6.99E-08	rs5756813	36505423	1.98E-01

For each SNP with the lowest P value (z-statistic) in individuals with European ancestry, we looked at the SNP with the lowest P value (z-statistic) in individuals with Asian ancestry within 100,000 basepairs to determine the differences between both ancestries.

Supplementary Table 4. The F_{st} values for the main association signals

rs4658101	0.32%
rs2623325	4.47%
rs17658229	0.68%
rs17756712	0.58%
rs7865618	6.80%
rs1900005	1.25%
rs7072574	0.26%
rs1346	0.18%
rs4936099	11.82%
rs11168187	0.22%
rs10862688	1.11%
rs4901977	5.42%
rs1345467	3.02%
rs6054374	6.60%
rs1547014	2.59%
rs301801	3.89%
rs868153	0.52%
rs5756813	1.77%

The F_{st} values show the percentage of genetic variation due to subdivision of populations.

Supplementary Table 5a. Adjustment for disc area

				β	SE	P value	β	SE	P value
SNP	Chr.	Nearest Gene	A1/A2	Caucasians unadjusted (*n* = 17,047)			Caucasians adjusted for DA (*n* = 16,980)		
rs4658101	1	*CDC7/TGFBR3*	a/g	0.014	0.002	3.48E-11	0.005	0.002	8.99E-03
rs2623325	3	*COL8A1*	a/c	0.023	0.004	4.44E-10	0.019	0.003	1.93E-08
rs17658229	5	*DUSP1*	c/t	-0.020	0.004	3.56E-08	-0.017	0.004	8.35E-07
rs17756712	6	*EXOC2*	g/a	0.010	0.002	1.07E-07	0.008	0.002	1.01E-05
rs7865618	9	*CDKN2BAS*	g/a	-0.013	0.002	5.02E-17	-0.013	0.001	5.79E-23
rs1900005	10	*ATOH7*	a/c	-0.020	0.002	5.11E-29	-0.010	0.002	7.28E-09
rs7072574	10	*PLCE1*	a/g	0.010	0.002	1.52E-09	0.008	0.002	5.70E-07
rs1346	11	*SSSCA1*	t/a	-0.014	0.002	3.66E-14	-0.012	0.002	6.20E-11
rs4936099	11	*ADAMTS8*	c/a	-0.009	0.002	6.48E-08	-0.008	0.001	7.08E-09
rs11168187	12	*RPAP3*	g/a	-0.009	0.002	6.17E-07	-0.009	0.002	5.20E-07
rs10862688	12	*TMTC2*	g/a	0.008	0.001	4.07E-09	0.006	0.001	5.04E-06
rs4901977	14	*SIX1/6*	t/c	0.011	0.002	1.54E-11	0.013	0.002	7.84E-15
rs1345467	16	*SALL1*	g/a	0.010	0.002	9.33E-10	0.007	0.002	5.27E-05
rs6054374	20	*BMP2*	t/c	-0.008	0.002	2.71E-07	-0.008	0.001	2.96E-10
rs1547014	22	*CHEK2*	t/c	-0.013	0.002	7.17E-16	-0.011	0.002	1.15E-11
rs301801	1	*RERE*	c/t	0.007	0.002	6.26E-06	0.008	0.002	5.00E-07
rs868153	6	*HSF2*	g/t	-0.006	0.002	9.00E-05	-0.006	0.002	2.62E-04
rs5756813	22	*CARD10*	g/t	0.007	0.002	1.55E-05	0.007	0.001	9.62E-07

SNPs that showed genome-wide significant ($P < 5 \times 10^{-8}$) association with vertical cup-disc ratio in subjects of European ancestry , adjusted for disc area. Note that the number of subjects is lower in the tables as several studies did not measure disc area.

A1 = reference allele; A2 = other allele; β = effect size on VCDR based on allele A1; Chr. = chromosome; DA = disc area; SE = standard error; SNP = single nucleotide polymorphism

Supplementary Table 5b. Adjustment for disc area

				β	SE	P value	β	SE	P value
SNP	Chr.	Nearest Gene	A1/A2	Asians unadjusted (*n* = 6,784)			Asians adjusted for DA (*n* = 6,694)		
rs4658101	1	*CDC7/TGFBR3*	a/g	0.016	0.005	3.13E-03	0.003	0.005	5.84E-01
rs2623325	3	*COL8A1*	a/c	0.011	0.005	1.46E-02	0.008	0.004	4.86E-02
rs17658229	5	*DUSP1*	c/t	-0.086	0.133	5.17E-01	-0.049	0.116	6.72E-01
rs17756712	6	*EXOC2*	g/a	0.011	0.005	1.76E-02	0.007	0.004	1.26E-01
rs7865618	9	*CDKN2BAS*	g/a	-0.021	0.005	8.11E-06	-0.018	0.004	1.87E-05
rs1900005	10	*ATOH7*	a/c	-0.010	0.004	2.08E-02	0.004	0.004	3.14E-01
rs7072574	10	*PLCE1*	a/g	0.007	0.003	4.80E-02	0.003	0.003	3.74E-01
rs1346	11	*SSSCA1*	t/a	0.003	0.005	5.23E-01	0.005	0.004	2.56E-01
rs4936099	11	*ADAMTS8*	c/a	-0.007	0.009	4.15E-01	-0.005	0.008	5.05E-01
rs11168187	12	*RPAP3*	g/a	-0.005	0.004	2.80E-01	-0.002	0.004	5.48E-01
rs10862688	12	*TMTC2*	g/a	0.004	0.003	2.48E-01	0.002	0.003	4.08E-01
rs4901977	14	*SIX1/6*	t/c	0.017	0.003	2.64E-07	0.018	0.003	5.09E-10
rs1345467	16	*SALL1*	g/a	0.011	0.006	5.53E-02	0.006	0.005	2.15E-01
rs6054374	20	*BMP2*	t/c	0.001	0.004	8.66E-01	0.001	0.004	8.26E-01
rs1547014	22	*CHEK2*	t/c	-0.013	0.004	4.26E-03	-0.010	0.004	8.01E-03
rs301801	1	*RERE*	c/t	0.012	0.005	2.59E-02	0.007	0.005	1.23E-01
rs868153	6	*HSF2*	g/t	-0.013	0.003	1.44E-04	-0.009	0.003	1.78E-03
rs5756813	22	*CARD10*	g/t	0.017	0.004	1.71E-06	0.012	0.003	2.34E-04

SNPs that showed genome-wide significant ($P < 5 \times 10^{-8}$) association with vertical cup-disc ratio in subjects of Asian ancestry , adjusted for disc area. Note that the number of subjects is lower in the tables as several studies did not measure disc area.

A1 = reference allele; A2 = other allele; β = effect size on VCDR based on allele A1; Chr. = chromosome; DA = disc area; SE = standard error; SNP = single nucleotide polymorphism

Supplementary Table 5c. Adjustment for disc area

				β	SE	P value	β	SE	P value
SNP	Chr.	Nearest Gene	A1/A2	Combined unadjusted (*n* = 23,831)			Combined adjusted for DA (*n* = 23,674)		
rs4658101	1	*CDC7/TGFBR3*	a/g	0.014	0.002	4.26E-13	0.005	0.002	9.04E-03
rs2623325	3	*COL8A1*	a/c	0.018	0.003	1.50E-10	0.015	0.003	2.70E-08
rs17658229	5	*DUSP1*	c/t	-0.020	0.004	3.56E-08	-0.017	0.004	8.35E-07
rs17756712	6	*EXOC2*	g/a	0.010	0.002	6.08E-09	0.008	0.002	3.19E-06
rs7865618	9	*CDKN2BAS*	g/a	-0.014	0.002	7.04E-21	-0.014	0.001	9.56E-27
rs1900005	10	*ATOH7*	a/c	-0.018	0.002	4.44E-29	-0.007	0.002	1.60E-06
rs7072574	10	*PLCE1*	a/g	0.009	0.002	2.98E-10	0.007	0.001	1.38E-06
rs1346	11	*SSSCA1*	t/a	-0.012	0.002	7.21E-12	-0.010	0.002	2.25E-08
rs4936099	11	*ADAMTS8*	c/a	-0.009	0.002	4.66E-08	-0.008	0.001	5.93E-09
rs11168187	12	*RPAP3*	g/a	-0.009	0.002	6.17E-07	-0.008	0.002	2.04E-06
rs10862688	12	*TMTC2*	g/a	0.007	0.001	4.32E-09	0.005	0.001	6.66E-06
rs4901977	14	*SIX1/6*	t/c	0.012	0.002	7.56E-17	0.014	0.001	8.89E-23
rs1345467	16	*SALL1*	g/a	0.010	0.002	1.44E-10	0.007	0.002	2.35E-05
rs6054374	20	*BMP2*	t/c	-0.007	0.002	2.20E-06	-0.007	0.001	5.65E-09
rs1547014	22	*CHEK2*	t/c	-0.013	0.002	1.15E-17	-0.011	0.002	3.23E-13
rs301801	1	*RERE*	c/t	0.008	0.002	6.42E-07	0.008	0.002	1.49E-07
rs868153	6	*HSF2*	g/t	-0.008	0.002	2.24E-07	-0.007	0.001	2.76E-06
rs5756813	22	*CARD10*	g/t	0.009	0.002	3.32E-09	0.007	0.001	2.62E-09

SNPs that showed genome-wide significant ($P < 5 \times 10^{-8}$) association with vertical cup-disc ratio in subjects of European and Asian ancestry combined, adjusted for disc area. Note that the number of subjects is lower in the tables as several studies did not measure disc area.

A1 = reference allele; A2 = other allele; β = effect size on VCDR based on allele A1; Chr. = chromosome; DA = disc area; SE = standard error; SNP = single nucleotide polymorphism

Supplementary Table 6. Pearson's correlation matrix for VCDR, spherical equivalent and disc area

Rotterdam Study I (n=5304)

	VCDR	DA	SE
VCDR	1	0.31	0.038
DA	0.31	1	-0.172
SE	0.038	-0.172	1

Rotterdam Study II (n=2051)

	VCDR	DA	SE
VCDR	1	0.356	0.093
DA	0.356	1	-0.139
SE	0.093	-0.139	1

Rotterdam Study III (n=1962)

	VCDR	DA	SE
VCDR	1	0.387	-0.052
DA	0.387	1	-0.076
SE	-0.052	-0.076	1

DA = disc area, SE = spherical equivalent, VCDR = vertical cup-disc ratio

Supplementary Table 7a. Adjustment for spherical equivalent

				β	SE	P value	β	SE	P value
SNP	Chr.	Nearest Gene	A1/A2	Caucasians unadjusted (*n* = 18,703)			Caucasians adjusted for SphE (*n* = 18,379)		
rs4658101	1	*CDC7/TGFBR3*	a/g	0.015	0.002	8.80E-14	0.013	0.002	6.25E-11
rs2623325	3	*COL8A1*	a/c	0.018	0.003	7.05E-09	0.017	0.004	1.69E-06
rs17658229	5	*DUSP1*	c/t	-0.020	0.004	8.06E-09	-0.022	0.004	2.83E-09
rs17756712	6	*EXOC2*	g/a	0.010	0.002	1.98E-08	0.010	0.002	2.37E-08
rs7865618	9	*CDKN2BAS*	g/a	-0.014	0.002	1.52E-19	-0.014	0.002	8.43E-18
rs1900005	10	*ATOH7*	a/c	-0.019	0.002	7.21E-31	-0.019	0.002	9.95E-28
rs7072574	10	*PLCE1*	a/g	0.009	0.002	6.17E-09	0.010	0.002	8.32E-10
rs1346	11	*SSSCA1*	t/a	-0.014	0.002	2.54E-15	-0.015	0.002	2.59E-15
rs4936099	11	*ADAMTS8*	c/a	-0.009	0.002	6.38E-09	-0.009	0.002	5.29E-08
rs11168187	12	*RPAP3*	g/a	-0.009	0.002	3.83E-07	-0.009	0.002	3.72E-06
rs10862688	12	*TMTC2*	g/a	0.008	0.001	2.27E-10	0.009	0.001	3.03E-11
rs4901977	14	*SIX1/6*	t/c	0.011	0.002	2.37E-11	0.010	0.002	6.24E-10
rs1345467	16	*SALL1*	g/a	0.010	0.002	2.45E-10	0.010	0.002	4.31E-09
rs6054374	20	*BMP2*	t/c	-0.009	0.002	1.79E-08	-0.008	0.002	1.37E-07
rs1547014	22	*CHEK2*	t/c	-0.013	0.002	2.39E-17	-0.013	0.002	1.98E-14
rs301801	1	*RERE*	c/t	0.007	0.002	2.52E-06	0.008	0.002	2.05E-06
rs868153	6	*HSF2*	g/t	-0.006	0.002	7.40E-05	-0.007	0.002	5.87E-05
rs5756813	22	*CARD10*	g/t	0.006	0.002	8.24E-05	0.006	0.002	9.91E-05

SNPs that showed genome-wide significant ($P < 5 \times 10^{-8}$) association with vertical cup-disc ratio in subjects of European ancestry , adjusted for spherical equivalent. Note that the number of subjects is lower in the tables as several studies did not measure disc spherical equivalent.

A1 = reference allele; A2 = other allele; β = effect size on VCDR based on allele A1; Chr. = chromosome; SE = standard error; SNP = single nucleotide polymorphism; SphE = spherical equivalent

Supplementary Table 7b. Adjustment for spherical equivalent

				β	SE	P value	β	SE	P value
SNP	Chr.	Nearest Gene	A1/A2	Asians unadjusted (*n* = 6,784)			Asians adjusted for SphE (*n* = 5,921)		
rs4658101	1	*CDC7/TGFBR3*	a/g	0.016	0.005	3.13E-03	0.017	0.006	3.62E-03
rs2623325	3	*COL8A1*	a/c	0.011	0.005	1.46E-02	0.012	0.005	1.80E-02
rs17658229	5	*DUSP1*	c/t	0.086	0.133	5.17E-01	-0.086	0.132	5.11E-01
rs17756712	6	*EXOC2*	g/a	0.011	0.005	1.76E-02	0.008	0.005	1.28E-01
rs7865618	9	*CDKN2BAS*	g/a	-0.021	0.005	8.11E-06	-0.023	0.005	5.12E-06
rs1900005	10	*ATOH7*	a/c	-0.010	0.004	2.08E-02	-0.012	0.004	8.50E-03
rs7072574	10	*PLCE1*	a/g	0.007	0.003	4.80E-02	0.006	0.004	1.28E-01
rs1346	11	*SSSCA1*	t/a	0.003	0.005	5.23E-01	-0.003	0.005	5.54E-01
rs4936099	11	*ADAMTS8*	c/a	-0.007	0.009	4.15E-01	-0.012	0.010	2.22E-01
rs11168187	12	*RPAP3*	g/a	-0.005	0.004	2.80E-01	-0.006	0.005	2.02E-01
rs10862688	12	*TMTC2*	g/a	0.004	0.003	2.48E-01	0.005	0.004	1.25E-01
rs4901977	14	*SIX1/6*	t/c	0.017	0.003	2.64E-07	0.019	0.004	5.11E-08
rs1345467	16	*SALL1*	g/a	0.011	0.006	5.53E-02	0.009	0.006	1.44E-01
rs6054374	20	*BMP2*	t/c	0.001	0.004	8.66E-01	0.001	0.004	8.43E-01
rs1547014	22	*CHEK2*	t/c	-0.013	0.004	4.26E-03	-0.009	0.005	5.98E-02
rs301801	1	*RERE*	c/t	0.012	0.005	2.59E-02	0.016	0.006	3.87E-03
rs868153	6	*HSF2*	g/t	-0.013	0.003	1.44E-04	-0.015	0.004	3.17E-05
rs5756813	22	*CARD10*	g/t	0.017	0.004	1.71E-06	0.018	0.004	4.13E-06

SNPs that showed genome-wide significant ($P < 5 \times 10^{-8}$) association with vertical cup-disc ratio in subjects of Asian ancestry , adjusted for spherical equivalent. Note that the number of subjects is lower in the tables as several studies did not measure disc spherical equivalent.

A1 = reference allele; A2 = other allele; β = effect size on VCDR based on allele A1; Chr. = chromosome; SE = standard error; SNP = single nucleotide polymorphism; SphE = spherical equivalent

Supplementary Table 7c. Adjustment for spherical equivalent

				β	SE	P value	β	SE	P value
SNP	Chr.	Nearest Gene	A1/A2	Combined unadjusted (*n* = 25,487)			Combined adjusted for SphE (*n* = 24,300)		
rs4658101	1	*CDC7/TGFBR3*	a/g	0.015	0.002	1.06E-15	0.014	0.002	9.55E-13
rs2623325	3	*COL8A1*	a/c	0.016	0.003	6.61E-10	0.015	0.003	1.36E-07
rs17658229	5	*DUSP1*	c/t	-0.020	0.004	8.06E-09	-0.022	0.004	2.83E-09
rs17756712	6	*EXOC2*	g/a	0.010	0.002	1.13E-09	0.010	0.002	8.06E-09
rs7865618	9	*CDKN2BAS*	g/a	-0.014	0.001	2.05E-23	-0.014	0.002	1.10E-21
rs1900005	10	*ATOH7*	a/c	-0.018	0.002	5.51E-31	-0.018	0.002	1.04E-28
rs7072574	10	*PLCE1*	a/g	0.009	0.001	1.02E-09	0.009	0.002	4.79E-10
rs1346	11	*SSSCA1*	t/a	-0.012	0.002	4.89E-13	-0.014	0.002	1.80E-14
rs4936099	11	*ADAMTS8*	c/a	-0.009	0.002	4.61E-09	-0.009	0.002	2.57E-08
rs11168187	12	*RPAP3*	g/a	-0.008	0.002	3.63E-07	-0.008	0.002	1.95E-06
rs10862688	12	*TMTC2*	g/a	0.008	0.001	2.68E-10	0.009	0.001	1.52E-11
rs4901977	14	*SIX1/6*	t/c	0.012	0.001	1.63E-16	0.012	0.002	2.65E-15
rs1345467	16	*SALL1*	g/a	0.010	0.002	3.80E-11	0.010	0.002	1.45E-09
rs6054374	20	*BMP2*	t/c	-0.007	0.001	1.69E-07	-0.007	0.002	1.02E-06
rs1547014	22	*CHEK2*	t/c	-0.013	0.002	3.85E-19	-0.012	0.002	4.28E-15
rs301801	1	*RERE*	c/t	0.008	0.002	2.65E-07	0.008	0.002	7.96E-08
rs868153	6	*HSF2*	g/t	-0.007	0.001	2.20E-07	-0.008	0.002	7.96E-08
rs5756813	22	*CARD10*	g/t	0.008	0.001	3.66E-08	0.008	0.002	7.73E-08

SNPs that showed genome-wide significant ($P < 5 \times 10^{-8}$) association with vertical cup-disc ratio in subjects of European and Asian ancestry combined, adjusted for spherical equivalent. Note that the number of subjects is lower in the tables as several studies did not measure disc spherical equivalent.

A1 = reference allele; A2 = other allele; β = effect size on VCDR based on allele A1; Chr. = chromosome; SE = standard error; SNP = single nucleotide polymorphism; SphE = spherical equivalent

Supplementary Table 8. Association with POAG in five case-control studies of SNPs that showed genome-wide significant ($P < 5 \times 10^{-8}$) association with vertical cup-disc ratio

				ANZRAG		deCODE		MEEI		NEIGHBOR		Southampton		Meta-analysis	
SNP	Chr.	Nearest Gene	A1/A2	OR	P value	OR	P value	OR	P value	OR	P value	OR	P value	OR	P value
rs4658101	1	*CDC7/TGFBR3*	a/g	1.06	3.58E-01	1.05	5.80E-01	1.49	2.12E-02	1.04	4.88E-01	0.98*	8.39E-01	1.06 (0.98-1.14)	1.22E-01
rs2623325	3	*COL8A1*	a/c	0.94	3.26E-01	1.03	7.50E-01	0.88	4.74E-01	1.11	1.06E-01	1.02**	8.56E-01	1.02 (0.95-1.09)	6.51E-01
rs17658229	5	*DUSP1*	c/t	1.10	4.70E-01	0.79	1.20E-01	0.73	4.67E-01	0.84	1.71E-01	0.27	9.42E-03	0.88 (0.76-1.02)	9.53E-02
rs17756712	6	*EXOC2*	g/a	1.03	7.07E-01	1.15	1.00E-01	1.30	1.54E-01	1.11	9.34E-02	0.97	8.15E-01	1.09 (1.01-1.17)	2.87E-02
rs7865618	9	*CDKN2BAS*	g/a	0.68	1.68E-12	0.94	3.50E-01	0.63	1.58E-03	0.67	7.31E-15	0.74	6.17E-05	0.73 (0.69-0.77)	<2.00E-16
rs1900005	10	*ATOH7*	a/c	1.12	6.24E-02	1.01	9.10E-01	1.02	8.96E-01	0.92	1.34E-01	1.00	9.78E-01	1.01 (0.94-1.08)	8.07E-01
rs7072574	10	*PLCE1*	a/g	1.11	6.11E-02	1.04	5.40E-01	0.98	8.75E-01	1.00	9.55E-01	1.09***	3.36E-01	1.05 (0.99-1.12)	9.91E-02
rs1346	11	*SSSCA1*	t/a	0.93	2.97E-01	1.03	7.30E-01	0.81	2.39E-01	0.90	1.03E-01	0.83	1.20E-01	0.92 (0.86-1.00)	3.66E-02
rs4936099	11	*ADAMTS8*	c/a	0.98	6.67E-01	0.99	8.20E-01	0.88	3.75E-01	0.96	3.88E-01	0.97	6.61E-01	0.97 (0.91-1.02)	2.40E-01
rs11168187	12	*RPAP3*	g/a	0.91	1.89E-01	0.88	1.60E-01	1.19	3.26E-01	0.94	3.74E-01	0.79	6.13E-02	0.92 (0.85-0.99)	2.53E-02
rs10862688	12	*TMTC2*	g/a	1.01	9.03E-01	1.11	9.70E-02	1.28	8.92E-02	1.06	2.45E-01	1.02	7.55E-01	1.06 (1.00-1.12)	5.36E-02
rs4901977	14	*SIX1/6*	t/c	1.29	7.99E-06	1.06	3.80E-01	1.58	2.02E-03	1.28	4.67E-06	0.97	7.90E-01	1.21 (1.14-1.29)	7.91E-10
rs1345467	16	*SALL1*	g/a	1.04	4.82E-01	1.00	9.70E-01	0.80	1.68E-01	1.09	1.30E-01	1.11	1.90E-01	1.04 (0.98-1.10)	1.61E-01
rs6054374	20	*BMP2*	t/c	0.95	3.82E-01	0.91	1.40E-01	0.92	5.69E-01	0.87	8.70E-03	0.97	6.74E-01	0.92 (0.87-0.97)	3.74E-03
rs1547014	22	*CHEK2*	t/c	0.97	5.54E-01	0.94	3.30E-01	0.93	6.39E-01	0.92	1.43E-01	0.95	5.06E-01	0.94 (0.89-1.00)	5.26E-02
rs301801	1	*RERE*	c/t	1.03	5.96E-01	1.00	9.60E-01	0.99	9.54E-01	1.11	4.38E-02	0.95	4.89E-01	1.03 (0.98-1.10)	2.53E-01
rs868153	6	*HSF2*	g/t	0.98	7.09E-01	0.96	5.70E-01	0.90	4.52E-01	0.84	9.19E-04	0.96	5.99E-01	0.92 (0.87-0.98)	7.06E-03
rs5756813	22	*CARD10*	g/t	1.05	4.12E-01	1.16	2.70E-02	1.19	2.00E-01	1.09	8.97E-02	1.08	3.11E-01	1.09 (1.03-1.16)	2.40E-03

Proxy SNPs were used for three missing SNPs in Southampton: * = rs1192415 (G allele), ** = rs2623320 (T allele), *** = rs4918188 (C allele). A1 = reference allele; A2 = other allele; Chr. = chromosome; OR = Odds ratio; SNP = single nucleotide polymorphism.

Supplementary Table 9. Results of a weighted risk score model in five case-control studies

	ANZRAG		deCODE		MEEI		NEIGHBOR		Southampton		Meta-analysis	
	OR	P value	OR	P value	OR	P value	OR	P value	OR	P value	OR	P value
1st quintile	1.00	-	1.00	-	1.00	-	1.00	-	1.00	-	1.00	-
2nd quintile	1.49	1.29E-03	0.94	6.50E-01	1.98	2.64E-02	1.50	3.35E-04	3.40	2.00E-02	1.39 (1.21-1.60)	3.15E-06
3rd quintile	1.71	1.35E-05	1.01	9.40E-01	1.43	2.39E-01	1.60	3.54E-05	2.97	4.10E-02	1.48 (1.29-1.70)	2.05E-08
4th quintile	1.78	3.20E-06	1.22	1.50E-01	1.75	7.16E-02	2.15	2.17E-11	3.18	2.90E-02	1.76 (1.53-2.01)	6.66E-16
5th quintile	2.44	2.80E-13	1.63	2.40E-04	3.47	1.67E-04	3.22	1.71E-23	5.10	1.00E-03	2.48 (2.17-2.84)	< 2.00E-16

A weighted genetic risk score per individual was calculated. Standardized regression coefficients of the eighteen top SNPs from the vertical cup-disc ratio analysis were used as weighting factor. The weighted risk scores were divided into quintiles. Odds ratios were calculated for each quintile, using the first quintile as a reference.

Supplementary Table 10. Genes where there is genome-wide significant evidence for association in gene-based tests

Gene	Chr.	Start Position (build 36)	Stop Position (build 36)	Gene-based empirical P value		
				European ancestry cohort	Asian ancestry cohort	Combined
SRP19	5	112224891	112231503	8.75E-05	0.00125	2.49E-06
REEP5	5	112239979	112285930	2.72E-05	0.001126	7.48E-07
CDKN2A	9	21957750	21984490	1.00E-08	0.000288	1.03E-10
CDKN2B	9	21992901	21999312	1.00E-08	0.000161	5.98E-11
MYPN	10	69539255	69641779	1.00E-08	0.067	1.38E-08
ATOH7	10	69660387	69661861	1.00E-08	0.00619	1.74E-09
PBLD	10	69712422	69762690	1.00E-08	0.00291	8.79E-10
HNRNPH3	10	69761773	69772959	1.00E-08	0.0119	3.13E-09
RUFY2	10	69773280	69837057	1.00E-08	0.0602	1.26E-08
DNA2	10	69843826	69901885	1.00E-08	0.326733	4.57E-08
SLC25A16	10	69912102	69957590	2.00E-08	0.366337	9.63E-08
SCYL1	11	65049123	65062758	1.00E-08	0.683168	6.80E-08
LTBP3	11	65062605	65082006	1.00E-08	0.792079	7.14E-08
SSSCA1	11	65094518	65095815	1.00E-08	0.930693	7.36E-08
FAM89B	11	65096395	65098245	1.00E-08	1	7.39E-08
EHBP1L1	11	65100084	65116692	1.00E-08	0.821782	7.20E-08
KCNK7	11	65116901	65120043	1.00E-08	0.811881	7.18E-08
MAP3K11	11	65121801	65138296	3.00E-08	0.841584	2.11E-07
PCNXL3	11	65140358	65161486	3.10E-07	0.871287	2.03E-06
PITPNB	22	26577656	26645255	1.20E-07	0.308	4.89E-07
CHEK2	22	27413730	27467822	1.00E-08	0.00272	8.27E-10
HSCB	22	27468042	27483496	1.00E-08	0.00401	1.18E-09
LGALS1	22	36401558	36405755	0.000225	0.000245	1.33E-06
NOL12	22	36412289	36419431	0.000127	3.32E-05	1.18E-07
TRIOBP	22	36423573	36502509	5.59E-05	3.85E-06	6.93E-09
H1F0	22	36531059	36533389	5.80E-05	5.04E-06	9.29E-09
GCAT	22	36533900	36542850	4.81E-05	8.16E-06	1.23E-08
GALR3	22	36549334	36551448	5.32E-05	2.14E-05	3.40E-08
ANKRD54	22	36556807	36570249	7.92E-05	0.000134	2.81E-07

Supplementary Table 11. Results from the gene-based test using alternative cutoff points from the transcription initiation and end sites and an LD binning approach (for this approach, we included SNPs within the gene boundary plus SNPs outside the gene boundary which were in r2>0.8 with SNPs within the gene).

	P values				
Gene	0kb + SNPs in LD with SNPs within the gene	0kb	10kb	20kb	50kb
ANKRD54	NA	NA	2.55E-04	2.52E-04	2.81E-07
ATOH7	NA	NA	5.60E-10	1.62E-09	1.74E-09
CDKN2A	3.88E-03	3.31E-03	1.64E-08	4.22E-09	1.03E-10
CDKN2B	1.34E-08	8.00E-09	1.58E-09	4.79E-10	5.98E-11
CHEK2	1.62E-10	1.24E-10	1.70E-10	1.59E-10	8.27E-10
DNA2	4.01E-08	3.80E-08	4.66E-08	4.74E-08	4.57E-08
EHBP1L1	6.87E-08	2.55E-07	7.31E-08	7.38E-08	7.20E-08
FAM89B	NA	NA	7.38E-08	7.20E-08	7.39E-08
GALR3	NA	NA	3.23E-04	1.42E-07	3.40E-08
GCAT	5.49E-08	4.49E-08	5.40E-08	2.27E-08	1.23E-08
H1F0	7.21E-08	NA	1.46E-08	2.81E-08	9.29E-09
HNRNPH3	2.54E-07	3.09E-07	2.16E-08	1.90E-08	3.13E-09
HSCB	1.88E-02	9.06E-03	4.17E-10	6.24E-10	1.18E-09
KCNK7	NA	NA	3.46E-06	6.02E-07	7.18E-08
LGALS1	NA	NA	1.56E-03	1.43E-03	1.33E-06
LTBP3	2.41E-02	5.73E-08	6.94E-08	7.29E-08	7.14E-08
MAP3K11	4.00E-05	2.90E-05	1.31E-05	3.47E-06	2.11E-07
MYPN	8.78E-08	9.17E-08	2.93E-08	2.64E-08	1.38E-08
NOL12	4.37E-02	4.73E-02	1.61E-02	5.94E-05	1.18E-07
PBLD	7.90E-10	8.82E-10	1.39E-09	1.06E-09	8.79E-10
PCNXL3	2.61E-03	3.74E-03	2.71E-03	1.07E-04	2.03E-06
PITPNB	2.37E-02	2.44E-02	1.03E-03	4.33E-04	4.89E-07
REEP5	1.50E-06	1.74E-06	1.32E-06	9.02E-07	7.48E-07
RUFY2	5.85E-08	5.98E-08	1.75E-07	3.63E-08	1.26E-08
SCYL1	7.33E-08	7.37E-08	7.37E-08	7.18E-08	6.80E-08
SLC25A16	4.59E-06	5.65E-06	4.98E-06	2.59E-09	9.63E-08
SRP19	1.74E-06	2.06E-06	1.77E-06	1.63E-06	2.49E-06
SSSCA1	7.32E-08	7.38E-08	7.39E-08	7.18E-08	7.36E-08
TRIOBP	3.50E-08	4.21E-08	4.03E-08	2.41E-08	6.93E-09

Supplementary Table 12. Glaucoma case-control gene-based results for regions of interest from vertical cup-disc ratio analysis

Chr.	Gene	n SNPs	Start	Stop	Gene-based P value			
					ANZRAG	NEIGHBOR	MEEI	Meta-analysis
5	*REEP5*	25	112239979	112285930	9.82E-01	8.83E-01	1.51E-01	6.68E-01
22	*PITPNB*	25	26577656	26645255	3.38E-02	9.39E-01	2.37E-01	1.34E-01

Supplementary Table 13. Results from pathway analysis

**GO ID**	**GO Term**	**Empirical P value**	**Genes**
GO:0045736	negative regulation of cyclin-dependent protein kinase activity	1.08E-06	*CASP3, APC, CDKN1A, LATS1, CDKN2A, MEN1, LATS2, HEXIM2, CDK5RAP1*
GO:0050680	negative regulation of epithelial cell proliferation	1.15E-05	*PLA2G2A, RUNX3, TGFBR3, TGFB2, SOX2, EREG, APC, PPARD, CDK6, NKX3-1, CDKN2B, GAS1, PTCH1, CDKN1C, KRT4, BRCA2, TINF2, NKX2-8, ESR2, TSC2, WFDC1, TGFB1*

Supplementary Table 14. Overview of DNaseI hypersensitivity sites located within 200 basepairs distance from each top SNP

SNP	Regulatory element	ENCODE annotation	Cell type (top signal)	N active/125 cell types	Position
rs4658101	DNaseI	Weak enhancer	adult dermal fibroblasts,	41	in cluster
rs2623325	--	--	--		
rs17658229	DNaseI	Weak transcribed region	skeletal muscle myoblasts	33	in cluster
rs17756712	DNaseI	Insulator	neonatal dermal fibroblast	62	in cluster
rs7865618	--	--	--		
rs1900005	--	--	--		
rs7072574	--	--	--		
rs1346	DNaseI	Active promotor	monocytes	125	in cluster
rs4936099	DNaseI	Repressed	pancreatic islets	2	within 200 bases
rs11168187	--	--	--		
rs10862688	--	--	--		
rs4901977	--	--	--		
rs1345467	--	--	--		
rs6054374	--	--	--		
rs1547014	DNaseI	Weak transcribed region	adult dermal fibroblasts	3	within 200 bases
rs301801	DNaseI	Weak transcribed region	retinal pigment epithelial cells	13	in cluster
rs868153	DNaseI	--	retinal pigment epithelial cells	23	within 200 bases
rs5756813	DNaseI	Weak transcribed region	mammary epithelial cells	25	in cluster

We used the ENCyclopedia Of DNA Elements (ENCODE)[1] data in the UCSC Genome Browser (available at: www.genome.ucsc.edu; accessed February 20th, 2014) to look at DNaseI hypersensitivity sites located within 200 basepairs distance from the most associated SNP. These sites are assessed in 125 cell types. The cell type with the top signal together with the number of cell types with active signals from these sites are presented.

Supplementary Table 15. Gene expression in human eye tissues

		Expression in 5 adult eye tissues[1]						Expression in 6 adult eye tissues[2]						
Chr.	Gene	Probe ID	Sclera	Cornea	Optic Nerve	Retina	RPE	NM_number	NPE	PE	TM	RPE	PHOT	choroid
			avg signal (P)	avg signal (P)	avg signal (P)	avg signal (P)	avg signal (P)							
1	*CDC7*	4900044	37.60 (0.02)	33.35 (0)	21.90 (0.04)	290.02 (0)	64.55 (0.04)	NM_003503	M	M	M	M	M	L
1	*TGFBR3*	3190379	556.02 (0)	968.25 (0)	483.22 (0)	101.68 (0)	595.28 (0)	NM_003243	M	M	M	M	L	M
1	*RERE*	10008	460.63 (0)	789.67 (0)	319.67 (0)	2008.78 (0)	799.5 (0)	NM_012102	M	M	M	M	M	M
3	*COL8A1*	2510091	3105.63 (0)	1349.35 (0)	421.48 (0)	26.67 (0.1)	1846.62 (0)	NM_001850	M	M	H	M	M	M
5	*DUSP1*	6860377	3722.75 (0)	4967.18 (0)	2490.72 (0)	5350.75 (0)	1293.38 (0)	NM_004417	H	H	H	-	-	-
5	*REEP5*	520324	981.65 (0)	1492.23 (0)	1109.12 (0)	5687.93 (0)	1535.57 (0)	NM_005669	H	H	H	H	H	H
6	*EXOC2*	2970390	44.37 (0)	58.15 (0)	35.85 (0)	242.82 (0)	71.95 (0)	NM_018303	M	M	M	M	M	M
6	*HSF2*	130121	58.97 (0.03)	151.47 (0)	51.57 (0)	491.73 (0)	92.20 (0)	NM_004506	M	M	M	M	M	M
9	*CDKN2BAS*	4200678	12.22 (0.31)	-0.23 (0.52)	-2.32 (0.54)	7.83 (0.56)	15.35 (0.30)	na	-	-	-	-	-	-
10	*ATOH7*	4480047	12.15 (0.33)	3.67 (0.34)	1.28 (0.37)	17.03 (0.26)	12.98 (0.4)	NM_145178	L	L	L	L	L	L
10	*PLCE1*	6220687	161.18 (0)	31.68 (0)	101.50 (0)	197.03 (0)	188.25 (0)	NM_016341	H	H	M	H	M	M
11	*ADAMTS8*	2480239	18.18 (0.17)	4.70 (0.28)	14.38 (0.11)	20.62 (0.15)	49.07 (0.07)	NM_007037	L	L	L	L	L	L
11	*SSSCA1*	6480605	14.92 (0.24)	46.23 (0)	9.23 (0.1)	25.38 (0.12)	23.77 (0.18)	NM_006396	M	M	M	M	M	M
12	*RPAP3*	2000392	42.33 (0.02)	127.73 (0)	24.82 (0.01)	151.97 (0)	35.90 (0.21)	NM_024604	-	-	-	M	M	M
12	*TMTC2*	3120184	17.13 (0.14)	19.57 (0.01)	19.53 (0.01)	100.08 (0)	21.65 (0.12)	NM_152588	M	M	M	L	L	L
14	*SIX1*	1450408	20.90 (0.20)	-4.10 (0.73)	31.98 (0.14)	7.93 (0.55)	17.30 (0.25)	NM_005982	L	L	M	L	L	M
14	*SIX6*	4260047	8.80 (0.49)	-1.68 (0.59)	54.97 (0)	594.35 (0)	103.73 (0.07)	NM_007374	H	H	L	M	M	M
16	*SALL1*	4610451	1.58 (0.82)	-5.85 (0.82)	8.70 (0.21)	14.08 (0.39)	3.92 (0.70)	NM_002968	VL	L	VL	M	M	L
20	*BMP2*	4280577	41.38 (0.01)	6.52 (0.18)	26.35 (0.01)	46.92 (0.01)	81.23 (0)	NM_001200	H	H	M	M	L	M
22	*CARD10*	3840678	na	263.50 (0)	28.75 (0.01)	na	na	NM_014550	M	M	M	L	L	M
22	*CHEK2*	5260685	65.37 (0)	100.93 (0)	23.93 (0.01)	23.32 (0.11)	53.55 (0.05)	NM_001005735, NM_007194	L	L	M	L	VL	L
22	*PITPNB*	1980687	84.88 (0.02)	96.85 (0)	43.42 (0)	121.38 (0)	111.97 (0.02)	NM_012399	M	M	M	M	M	M

1. In the first 5 adult human eye tissues, the avg sig represents the gene expression[2]. The avg sig is the average of detected signals of all probes for each single gene on the Illumina HumanHT-12-v4 Expression BeadChips after background noisy subtraction. The signal detection p-value (p-value <0.05) defines the gene expression. Average signal of 28 is the background expression signal.

2. With microarray studies we performed gene expression analysis on separately dissected non-pigmented (NPE) and pigmented epithelia (PE) of the ciliary body epithelia, trabecular meshwork (TM), photoreceptor cells (PHOT), retinal pigment epithelium (RPE) and choroid. The data of these tissues are included in the GEO database: GSE37957 (NPE, PE), GSE50784 (TM) and GSE20191 (PHOT, RPE, choroid). For detailed description of the microarray procedures, see Booij et al. BMC Genomics 2009[3] and Janssen et al. PlosOne 2012[4]. We ranked the genes by expression level and assigned percentile ranks (P). Next, we formed four groups: high (H) expression (expression >90th P), moderate (M) expression (50-90th P), low (L) expression (10-50th P) and very low (VL) expression (<10th P). This means that a gene in the high expression group (>90th P) has an expression intensity that falls into the highest 10% intensity values of the microarray, whereas a gene in the very low expression group (<10th P) has an expression intensity in the lowest 10% intensity values of the microarray (methodology according to Booij et al. BMC Genomics 2009). For three genes, *DUSP1*, *CDKN2BAS* and *RPAP3*, expression intensities were missing for some tissues. The expression intensities of *CDKN2B* were low for all tissues.

Other abbreviations: avg = average; chr. = chromosome; na = not applicable; P = P value

Supplementary Table 16. Gene expression in human eye tissues (online available)

		EyeSAGE[1]				The Ocular Tissue Database[2]					
Chr.	Gene	TM	Retina Macular	RPE Macular	RPE Peripheral	Probe ID	Sclera	Cornea	Optic Nerve	TM	Retina
							PLIER	PLIER	PLIER	PLIER	PLIER
1	*CDC7*	+	-	+	+	2346399	14.1437	26.2526	13.3414	31.3827	42.0588
1	*TGFBR3*	+	+	+	+	2422722	54.2114	48.6702	33.6028	129.423	20.5432
1	*RERE*	+	-	+	+	2395245	69.0848	98.9788	81.2431	99.1054	97.577
3	*COL8A1*	+	-	-	+	2633390	86.785	67.5133	18.0607	52.2185	11.06
5	*DUSP1*	+	+	+	+	2887309	344.877	175.895	152.288	288.982	109.293
5	*REEP5*	+	na	na	na	2871176	107.365	160.873	177.741	127.828	131.439
6	*EXOC2*	+	na	na	na	2938196	36.702	29.802	28.0297	32.3478	35.2114
6	*HSF2*	+	+	-	-	2923819	26.8471	56.2374	38.1092	41.0938	84.9574
9	*CDKN2BAS*	na	na	na	na	3164914	31.6883	36.9869	33.3128	33.4433	26.0191
10	*ATOH7*	na	na	na	na	3292561	5.84118	11.295	4.64013	2.8083	9.37729
10	*PLCE1*	+	+	+	+	3258477	140.073	26.7803	165.914	143.237	60.5558
11	*ADAMTS8*	na	na	na	na	3398328	31.8785	27.8784	26.1419	26.0516	24.3471
11	*SSSCA1*	na	+	+	+	3335327	18.5191	23.2121	6.36679	9.22546	18.4087
12	*RPAP3*	na	na	na	na	3452622	52.9563	74.9094	63.1802	73.1841	47.3673
12	*TMTC2*	+	na	na	na	3424442	17.7274	50.6204	80.9089	70.4686	40.9726
14	*SIX1*	+	+	-	+	3567333	25.0362	9.65488	18.3224	15.4274	14.7294
14	*SIX6*	+	+	+	+	3538624	19.0166	27.3315	22.2523	25.7996	26.0817
16	*SALL1*	na	+	+	+	3691326	43.9266	47.3643	80.9887	43.8865	45.5443
20	*BMP2*	na	na	na	na	3875423	26.0481	16.909	17.0003	33.4738	21.0732
22	*CARD10*	+	na	na	na	3960133	83.7697	68.6066	66.9579	66.632	69.6956
22	*CHEK2*	+	-	+	+	3941623	39.9337	31.5934	39.2161	38.5951	24.0604
22	*PITPNB*	+	+	+	+	3956290	233.442	344.688	226.684	364.335	164.843

1. In the EyeSAGE datasets from NEIBank[5,6], the gene expression is determined by tag counts in the Serial Analysis of Gene Expression (SAGE). We summarized all counts for each gene per tissue and put ‘+’ to indicate the expression; put ‘-‘ to label the no expression while the counts are 0. The EyeSAGE is publicly available at the http://neibank.nei.nih.gov/EyeSAGE/index.shtml.

2. In the Ocular Tissue Database (OTDB)[7], the gene expression is indicated as Affymetrix Probe Logarithmic Intensity Error (PLIER) normalized value. The PLIER normalization method was described by Wagner et al[7]. The OTDB is available at https://genome.uiowa.edu/otdb/.

Abbreviations: chr. = chromosome; na = not applicable; RPE = retinal pigment epithelium; TM = trabecular meshwork

Supplementary Table 17a. Phenotyping methods

Study	Measurement of vertical cup-disc ratio
BATS	Nidek 3-Dx fundus camera (Nidek, Gamagori, Japan) with custom planimetric software (StereoDx, using a Z-screen; StereoGraphics Corp., Beverly Hills, CA, USA)
BMES	30º color stereoscopic optic disc photographs taken with a 99 Zeiss FF3 fundus camera
ERF	Heidelberg Retina Tomograph 2
GHS I	Visucam ProNM and Visupac (Carl Zeiss Meditec AG, Jena, Germany)
GHS II	Visucam ProNM and Visupac (Carl Zeiss Meditec AG, Jena, Germany)
Glaugen	Retrieved from medical records
Neighbor	Retrieved from medical records
RAINE	Heidelberg Retina Tomograph 3, Heidelberg Engineering, Heidelberg, Germany
RS-I	ImageNet and stereoscopic fundus camera (Topcon TRC-SS2; Tokyo Optical Co., Tokyo, Japan)
RS-II	ImageNet and stereoscopic fundus camera (Topcon TRC-SS2; Tokyo Optical Co., Tokyo, Japan)
RS-III	Heidelberg Retina Tomograph 2, Heidelberg Engineering, Heidelberg, Germany
TEST	Nidek 3-Dx fundus camera (Nidek, Gamagori, Japan) with custom planimetric software (StereoDx, using a Z-screen; StereoGraphics Corp., Beverly Hills, CA, USA)
TwinsUK	Nidek 3-Dx fundus camera (Nidek, Gamagori, Japan) with custom planimetric software (StereoDx, using a Z-screen; StereoGraphics Corp., Beverly Hills, CA, USA)
BES	Planimetry, VCDR was manually calculated
SCES	Heidelberg Retina Tomography 2, Heidelberg Engineering, Heidelberg, Germany
SINDI	Heidelberg Retina Tomography 2, Heidelberg Engineering, Heidelberg, Germany
SIMES	Heidelberg Retina Tomography 2, Heidelberg Engineering, Heidelberg, Germany

Supplementary Table 17b. Genotyping methods

Study	Genotyping chip
BATS	Illumina HumanHap 610W Quad arrays (Illumina Inc., San Diego, CA, USA)
BMES	Illumina Human660W Quad array
ERF	Illumina 6k; Illumina 318K; Illumina 370K; Affymetrix 250K
GHS I	Affymetrix Genome-Wide Human SNP 6.0 Array
GHS II	Affymetrix Genome-Wide Human SNP 6.0 Array
Glaugen	Illumina 660W Quad Array
Neighbor	Illumina 660W Quad Array
RAINE	Illumina 660W Quad Array
RS-I	Illumina Infinium II HumanHap550 chip v3.0 array
RS-II	HumanHap550 Duo Arrays + Human610-Quad Arrays Illumina
RS-III	Human 610 Quad Arrays Illumina
TEST	Illumina HumanHap 610W Quad arrays (Illumina Inc., San Diego, CA, USA)
TwinsUK	Illumina 300K Duo and HumanHap610-Quad arrays
BES	Illumina610
SCES	Illumina610
SINDI	Illumina610
SIMES	Illumina610
ANZRAG	Illumina Omni-1M and Illumina Omni-Express arrays
deCODE	Illumina HumanHap300; HumanCNV370; HumanHap610; HumanHap1M; HumanHap660; Omni-1; Omni 2.5; Omni Express
Southampton	Affymetrix SNP 6.0 array

Supplementary Note: Acknowledgements

We would like to acknowledge the following persons and agencies:

Australian & New Zealand Registry of Advanced Glaucoma (ANZRAG)

Support for recruitment of ANZRAG was provided by the Royal Australian and New Zealand College of Ophthalmology (RANZCO) Eye Foundation. Genotyping was funded by the National Health and Medical Research Council of Australia (#535074 and #1023911). The authors acknowledge the support of Ms Bronwyn Usher-Ridge and Ms Emmanuelle Souzeau in patient recruitment and data collection, Professor Matthew A.Brown and Dr Patrick Danoy for genotyping and Mr Rhys Fogarty and Dr Matthew Law for data analysis.

Beijing Eye Study (JJES)

Beijing Eye Study was supported by National Natural Science Foundation of China (grant 81170890).

Blue Mountains Eye Study

BMES was supported by the Australian National Health & Medical Research Council (NH&MRC), Canberra Australia (974159, 211069, 457349, 512423, 475604, 529912); the Centre for Clinical Research Excellence in Translational Clinical Research in Eye Diseases; NH&MRC research fellowships (358702, 632909 to J.J.W, 1028444 to P.N.B.); and the Wellcome Trust, UK as part of Wellcome Trust Case Control Consortium 2 (A. Viswanathan, P. McGuffin, P. Mitchell, F. Topouzis, P. Foster) for genotyping costs of the entire BMES population (085475/B/08/Z, 085475/Z/08/Z, 076113).

The Centre for Eye Research Australia receives Operational Infrastructure Support from the Victorian government. BMES acknowledges Elena Rochtchina from the Centre for Vision Research, Department of Ophthalmology and Westmead Millennium Institute University of Sydney (NSW Australia); John Attia, Rodney Scott, Elizabeth G. Holliday from the University of Newcastle (Newcastle, NSW Australia); Jing Xie, and Andrea J. Richardson from the Centre for Eye Research Australia, University of Melbourne; Michael T. Inouye, Medical Systems Biology, Department of Pathology & Department of Microbiology & Immunology, University of Melbourne (Victoria, Australia); Ananth Viswanathan, Moorfields Eye Hospital (London, UK); Paul J. Foster, NIHR Biomedical Research Centre for Ophthalmology, UCL Institute of Ophthalmology & Moorfields Eye Hospital (London); Peter McGuffin, MRC Social Genetic and Developmental Psychiatry Research Centre, Institute of Psychiatry, King's College (London, United Kingdom); Fotis Topouzis, Department of Ophthalmology, School of Medicine, Aristotle University of Thessaloniki, AHEPA Hospital (Thessaloniki, Greece); Xueling Sim, National University of Singapore.

deCODE

We thank all the participants whose contribution made this study possible, as well as their ophthalmologists. We also thank the personnel at deCODE recruitment center and core facilities for their hard work and enthusiasm.

Erasmus Rucphen Family (ERF) Study and *Rotterdam Study*

The Rotterdam Study and ERF were supported by the Netherlands Organisation of Scientific Research (NWO; 91111025); Erasmus Medical Center and Erasmus University, Rotterdam, The Netherlands; Netherlands Organization for Health Research and Development (ZonMw); UitZicht; the Research Institute for Diseases in the Elderly; the Ministry of Education, Culture and Science; the Ministry for Health, Welfare and Sports; the European Commission (DG XII); the Municipality of Rotterdam; the Netherlands Genomics Initiative/NWO; Center for Medical Systems Biology of NGI; Stichting Lijf en Leven; Stichting Oogfonds Nederland; Landelijke Stichting voor Blinden en Slechtzienden; Algemene Nederlandse Vereniging ter Voorkoming van Blindheid; Medical Workshop; Heidelberg Engineering; Topcon Europe BV. Henriët Springelkamp is supported by the NWO Graduate Programme 2010 BOO (022.002.023). We acknowledge the contribution of Ada Hooghart, Corina Brussee, Riet Bernaerts-Biskop, Patricia van Hilten, Pascal Arp, Jeanette Vergeer, Maarten Kooijman and Virginie Verhoeven.

The generation and management of GWAS genotype data for the Rotterdam Study is supported by the Netherlands Organisation of Scientific Research NWO Investments (nr. 175.010.2005.011, 911-03-012). This study is funded by the Research Institute for Diseases in the Elderly (014-93-015; RIDE2), the Netherlands Genomics Initiative (NGI)/Netherlands Organisation for Scientific Research (NWO) project nr. 050-060-810. We thank Pascal Arp, Mila Jhamai, Marijn Verkerk, Lizbeth Herrera and Marjolein Peters for their help in creating the GWAS database, and Karol Estrada and Maksim V. Struchalin for their support in creation and analysis of imputed data.

The authors are grateful to the study participants, the staff from the Rotterdam Study and the participating general practitioners and pharmacists.

Glaucoma Genes and Environment (GLAUGEN) and *National Eye Institute (NEI) Glaucoma Human Genetics Collaboration (NEIGHBOR)*

Genotyping services for the NEIGHBOR study were provided by the Center for Inherited Disease Research (CIDR) and were supported by the National Eye Institute through grant HG005259-01 (JL Wiggs). Additionally, CIDR is funded through a federal contract from the National Institutes of Health to The Johns Hopkins University, contract number HHSN268200782096C. Genotyping for the GLAUGEN dataset at the Broad Institute was supported by GENEVA project grant HG004728 (LR Pasquale) and U01-HG004424 (Broad Institute). Genotype data cleaning and analysis for the GLAUGEN study was supported by U01 HG004446 (C Laurie). Collecting and processing samples for the NEIGHBOR dataset was supported by the National Eye Institute through ARRA grants 3R01EY015872-05S1 (JL Wiggs) and 3R01EY019126-02S1 (MA Hauser). Funding for the collection of cases and controls was provided by NIH grants: EY015543 (RR Allingham), EY006827 (D Gaasterland); HL73042, HL073389, EY13315 (MA Hauser); CA87969 (JH Kang), CA49449 (JH Kang), CA55075 (JH Kang), EY009149 (PR Lichter), HG004608 (C McCarty), EY008208 (FA Medeiros), EY015473 (LR Pasquale), EY012118 (M Pericak-Vance), EY015682 (A Realini), EY011671 (JE Richards), EY09580 (JE Richards), EY013178 (JS Schuman), RR015574, EY015872 (JL Wiggs), EY010886 (JL Wiggs), EY009847 (JL Wiggs), R01EY022305 (JL Wiggs), P30EY014104 (JL Wiggs), EY011008, EY144428 (K Zhang), EY144448 (K Zhang), EY18660 (K Zhang). JL Wiggs and LR Pasquale are also supported by the Harvard Glaucoma Center for Excellence and the Margolis Fund. Y Liu is supported by the Glaucoma Research Foundation, American Health Assistance Foundation, and the Glaucoma Foundation. Jessica N. Cooke Bailey was previously supported by Vanderbilt University (NIH T32 EY21453-2) and is currently

supported by Case Western Reserve University (NIH T32 EY007157). JL Wiggs, LR Pasquale, DC Musch, and JE Richards are supported by Research to Prevent Blindness.

Gutenberg Health Study (GHS I, GHS II)

The Gutenberg Health Study was funded through the government of Rhineland-Palatine ("Stiftung Rheinland Pfalz für Innovation" (AZ 961-386261/733); the research programs "Wissen schafft Zukunft" and "Schwerpunkt Vaskuläre Prävention" of the Johannes Gutenberg-University of Mainz; Boehringer Ingelheim, Ingelheim, Germany; PHILIPS Medical Systems, Hamburg, Germany; National Genome Network ''NGFNplus'' by the Federal Ministry of Education and Research, Germany (A301GS0833).

Raine Study

The core management of the Raine Study is funded by The University of Western Australia (UWA), The Telethon Institute for Child Health Research, Raine Medical Research Foundation, UWA Faculty of Medicine, Dentistry and Health Sciences, Women's and Infant's Research Foundation and Curtin University. Genotyping was funded by NHMRC project grant 572613. Support for the Raine Eye Health Study was provided by NHMRC Grant 1021105, Lions Eye Institute, the Australian Foundation for the Prevention of Blindness, Ophthalmic Research Institute of Australia and Alcon Research Institute. The Raine Eye Health Study authors thank the Raine eye health study participants and their families. They also thank the Raine Study management, Craig E Pennell and the team at TICHR and LEI for cohort co-ordination and data collection, particularly: Charlotte McKnight, Seyhan Yazar, Hannah Forward, Wei Ang, Alex Tan, Alla Soloshenko, Sandra Oates, and Diane Wood.

Singapore (SCES, SIMES, SINDI)

We acknowledge the following source of funding support : National Medical Research Council, Singapore (NMRC/TCR/002-SERI/2008 (R626/47/2008TCR), CSA R613/34/2008, NMRC 0796/2003, STaR/0003/2008), the National Research Foundation of Singapore, the Biomedical Research Council, Singapore (BMRC 09/1/35/ 19/616, 08/1/35/19/550, 10/1/35/19/675) and Genome Institute of Singapore (GIS/12-AR2105). The Singapore Tissue Network and the Genome Institute of Singapore, Agency for Science, Technology and Research, Singapore provided services.

Southampton

We thank Marie Nelson, Catrin Watkins, Georgina Matei, and the Southampton Wellcome Trust Clinical Research Facility for research nurse support in collecting DNA samples and all the patients who contributed to this work. Funding for this work was provided by: Optegra, UK and Eire Glaucoma Society, International Glaucoma Association (in association with the Royal College of Ophthalmologists), T F C Frost Charitable Trust and Gift of Sight.

Twins Eye Study in Tasmania (TEST) and *Brisbane Adolescent Twin Study (BATS)*

TEST and BATS (Australian Twins) were supported by an Australian National Health and Medical Research Council (NHMRC) Enabling Grant (2004-2009, 350415, 2005-2007); Clifford Craig Medical Research Trust; Ophthalmic Research Institute of Australia; American Health Assistance Foundation; Peggy and Leslie Cranbourne Foundation; Foundation for Children; Jack Brockhoff Foundation;

National Institutes of Health/National Eye Institute (RO1EY01824601 (2007-2010)); Pfizer Australia Senior Research Fellowship (to D.A.M.); and Australian NHMRC Career Development Award (to S.M.). Genotyping was funded by an NHMRC Medical Genomics Grant; US National Institutes of Health/National Eye Institute (1RO1EY018246), Australian sample imputation analyses were carried out on the Genetic Cluster Computer which is financially supported by the Netherlands Scientific Organization (NWO48005003). Australian Twins thanks Nicholas Martin, Scott Gordon, Dale Nyholt, Sarah Medland, Brian McEvoy, Margaret Wright, Anjali Henders, Megan Campbell for ascertaining and processing genotyping data; Jane MacKinnon, Shayne Brown, Lisa Kearns, Jonathan Ruddle, Sandra Staffieri, Olivia Bigault, Colleen Wilkinson, Byoung Sung Chu, Robert Macmillan, Johan Poulsen, Yaling Ma, Julie Barbour for assisting with clinical examinations; and Dr Camilla Day and staff at the Center for Inherited Disease Research.

TwinsUK

TwinsUK received funding from the Wellcome Trust; the European Union MyEuropia Marie Curie Research Training Network; Guide Dogs for the Blind Association; the European Community's FP7 (HEALTHF22008201865GEFOS); ENGAGE (HEALTHF42007201413); the FP-5 GenomEUtwin Project (QLG2CT200201254); US National Institutes of Health/National Eye Institute (1RO1EY018246); NIH Center for Inherited Disease Research; the National Institute for Health Research (NIHR)-funded BioResource, Clinical Research Facility and Biomedical Research Centre based at Guy's and St. Thomas' National Health Service Foundation Trust in partnership with King's College London. A.N. received funding from Fight for Sight and The Worshipful Company of Spectacle Makers. P.G.H. is the recipient of a Fight for Sight ECI award. C.J.H. is an NIHR Senior Research fellow. We acknowledge the contribution of Drs Toby Andrew, Margarida Lopes, Samantha Fahy and Diana Kozareva.

Terri L. Young and Xiaoyan Luo received funding from the National Institutes of Health, National Eye Institute (grants R01 EY014685 and R01 EY018246).

Supplementary Methods

Australian & New Zealand Registry of Advanced Glaucoma (ANZRAG)

The Australian & New Zealand Registry of Advanced Glaucoma (ANZRAG) recruits cases of advanced glaucoma Australia-wide through ophthalmologist referral. The cohort also included participants enrolled in the Glaucoma Inheritance Study in Tasmania (GIST) that met the criteria for ANZRAG. This cohort has been described previously[8]. Advanced Primary Open Angle Glaucoma was defined as best-corrected visual acuity worse than 6/60 due to primary open angle glaucoma, or a reliable 24-2 Visual Field with a mean deviation of worse than -22db or at least 2 out of 4 central fixation squares affected with a Pattern Standard Deviation of < 0.5%. The less severely affected eye was also required to have signs of glaucomatous disc damage. Clinical exclusion criteria included: i) pseudoexfoliation or pigmentary glaucoma, ii) angle closure or mixed mechanism glaucoma; iii) secondary glaucoma due to aphakia, rubella, rubeosis or inflammation; iv) infantile glaucoma, v) glaucoma in the presence of a known associated syndrome.

Controls were drawn from the Australian Cancer Study (225 esophageal cancer cases, 317 Barrett's esophagus cases and 552 controls) or from a study of inflammatory bowel diseases (303 cases and 595 controls). All were Australians of European ancestry. All participants provided written informed consent. Approval was obtained from the Human Research Ethics Committees of Southern Adelaide Health Service/Flinders University, University of Tasmania, Queensland Institute of Medical Research and Royal Victorian Eye and Ear Hospital.

In total there were 1,155 glaucoma cases and 1,992 controls. DNA was extracted from peripheral whole blood. Genotyping was conducted using Illumina omni-1M or omni-express arrays. Cases and controls genotypes for the 569,249 SNPs common to the arrays were taken forward for analysis. Principle components were computed for all participants and reference samples of known northern European ancestry (1000G British, CEU, Finland participants). Participants with PC1 or PC2 values >6 standard deviations from the known northern European ancestry group were excluded. Identity by state was computed based on autosomal markers, with individuals with relatedness >0.2 removed. Association testing was conducted using logistic regression with sex and the first 6 principle components fitted as covariates. Imputation was conducted using IMPUTE2[9], with 1000 Genomes Phase 1 Europeans as the reference panel. Association testing was done using SNPTEST. SNPs of interest from the VCDR analysis were selected to assess association with POAG.

Beijing Eye Study (BES)

The BES is a population-based cohort of Han Chinese in the rural region and in the urban region of Beijing in North China[10,11]. The Medical Ethics Committee of the Beijing Tongren Hospital approved the study protocol and all participants gave informed consent, according to the Declaration of Helsinki. At baseline (2001), 4439 individuals out of 5324 eligible individuals aged 40 years or older participated (response rate: 83.4%). In the years 2006 and 2011, the study was repeated by re-inviting all participants from the survey from 2001 to be re-examined. Out of the 4439 subjects examined in 2001, 3251 (73.2%) subjects returned for the follow-up examination in 2006, and 2695 (60.7%) subjects returned for the follow-up examination in 2011. All study participants underwent an ophthalmic examination including refractometry, pneumotonometry, slit-lamp biomicroscopy, and photography of the cornea, lens, optic disk, and macula. Optic disc parameters were measured using

Planimetry and the vertical cup-disc ratio was manually calculated. Blood samples were taken from 2,929 (90.1%), and DNA was extracted from blood leucocytes according to standard procedures. We performed genotyping using Illumina Human610-Quad BeadChip in 988 subjects[12]. 151 individuals with cryptic relatedness were excluded during sample QC procedure. After the removal of samples, SNPs were excluded based on (i) high rates of missingness (>5%); (ii) monomorphism; (iii) gross departure from HWE of $p<10^{-6}$. Imputation was performed using IMPUTE v2.2.2[9] on post-QC SNPs. The HapMap Phase II panel (build36, release 22 db126 JPT+CHB HapMap panel) was used for the imputation.

Blue Mountains Eye Study (BMES)

BMES was a population-based eye assessment of a representative older Australian community sample. During the period 1992-1994, 3654 residents (57% male and 43% females) aged 49-97 were examined (BMES-1). The study was approved by the Western Sydney Area Health Service Human Ethics Committee, and written, informed consent was obtained from all participants. All participants had undertaken a detailed eye examination including stereo photographs in order to calculate the diameter of the vertical optic disc and the vertical cup. Vertical disc (VDD) and cup diameter (VCD) were obtained after pupil dilation from 30º color stereoscopic optic disc photographs taken with a 99 Zeiss FF3 fundus camera (Carl Zeiss Meditec, Dublin, CA). Vertical CDR was calculated by the vertical disc and cup measurements. Further details have been described elsewhere[13]. Samples were genotyped on the Human660W-Quad. Imputation was performed with IMPUTE2 which adopts a two-stage approach using both haploid and diploid reference panels. 1000 Genome pilot data was used as reference panel. The SNPs considered in this study were those present also in HapMap2.

deCODE

The Icelandic primary open angle glaucoma (POAG) cases were identified from a list of participants in the Reykjavik Eye Study[14] and from a list compiled by Icelandic ophthalmologists in 2008 that included patients 55-86 years old at the time of diagnosis, all meeting either structural (glaucomatous optic neuropathy) or functional (glaucomatous visual field defects) criteria of glaucoma or both. For visual fields measurements, Octopus 123 perimeter (Haag-Streit AG, Koniz Switzerland) was used. Intra ocular pressure was not a part of the definition. On gonioscopy the angles were found to be open and normal in appearance. The combined list includes 598 individuals, 290 men and 308 women with POAG. The diagnosis of exfoliation syndrome (XFS) was specifically evaluated and if detected the participant was excluded from the study. The control group included 98,670 Icelandic individuals without know history of glaucoma. 533 of the POAG samples and 85,689 of the controls samples were assayed with the Illumina HumanHap300, HumanCNV370, HumanHap610, HumanHap1M, HumanHap660, Omni-1, Omni 2.5 or Omni Express bead chips at deCODE genetics and genotypes for about 34 million sequence variants were imputed into the chip typed individuals based on a training set of 2,230 whole genome sequenced Icelanders using methods previously described[15]. The case-control association analysis was done on the imputed genotypes, including in addition 65 cases and 12,981 controls that were not chip typed but for which genotype probabilities were imputed using methods of familial imputation[15]. For the risk score analysis only chip typed individuals were used. The study was approved by the Icelandic National Bioethics Committee and by the Icelandic Data Protection Authority. Informed consent was obtained

from all participants. The study was conducted in accordance with revised Declaration of Helsinki.

Erasmus Rucphen Family (ERF) Study

The Erasmus Rucphen Family (ERF) Study is a family-based cohort in a genetically isolated population in the southwest of the Netherlands with over 3,000 participants aged between 18 and 86 years[16,17]. Cross-sectional examination took place between 2002 and 2005. Heidelberg Retina Tomograph 2 was used to measure the VCDR. Details have been described elsewhere[18]. All measurements in these studies were conducted after the Medical Ethics Committee of the Erasmus University had approved the study protocols and all participants had given a written informed consent in accordance with the Declaration of Helsinki. DNA was genotyped on one of four different platforms (Illumina 6k, Illumina 318K, Illumina 370K and Affymetrix 250K), which were then merged. Samples with low call rate (<97.5%), with excess autosomal heterozygosity (>0.336), or with sex-mismatch were excluded. A set of genotyped input SNPs with call rate >98%, with minor allele frequency >0.01, and with Hardy-Weinberg P value $>10^{-6}$ was used for imputation. We used the Markov Chain Haplotyping (MaCH) package version 1.0.18.c software (Rotterdam, The Netherlands; imputed to plus strand of NCBI build 36, HapMap release #22) and minimac version 2011.10.27 for the analyses. For each imputed SNP, a reliability of imputation was estimated as the ratio of the empirically observed dosage variance to the expected binomial dosage variance (O/E ratio). GWAS analyses were performed using the ProbABEL package. Mmscore models were used to correct for family structure.

Gutenberg Health Study (GHS I, GHS II)

The GHS is a population-based, prospective, observational cohort study in the Rhine-Main Region in midwestern Germany with a total of 15,010 participants and follow-up after five years. The study sample is recruited from subjects aged between 35 and 74 years at the time of the exam. The sample was drawn randomly from local governmental registry offices and stratified by gender, residence (urban and rural) and decade of age. Exclusion criteria were insufficient knowledge of the German language to understand explanations and instructions, and physical or psychic inability to participate in the examinations in the study center. The study was approved by the Medical Ethics Committee of the University Medical Center Mainz and by the local and federal data safety commissioners. According to the tenets of the Declaration of Helsinki, written informed consent was obtained from all participants prior to entering the study.

Within GHS, DNA was extracted from buffy-coats from EDTA blood samples as described earlier[19]. Genetic analysis was conducted in the first 5,000 study participants. For these, 3,463 individuals were genotyped in 2008 (GHS I) and further 1,439 individuals in 2009 (GHS II). Genotyping was performed for GHS I and GHS II using the Affymetrix Genome-Wide Human SNP Array 6.0. Genotypes were called using the Affymetrix Birdseed-V2 calling algorithm. Individuals with a call rate below 97% or a too high autosomal heterozygosity (3 SD from mean) and sex-mismatches were excluded. After applying standard quality criteria (MAF >1%, genotype call rate >98% and *P*-value of deviation from HWE of $>10^{-4}$), 675,350 SNPs in 2750 individuals from GHS I and 673,914 SNPs in 1,143 individuals from GHS II remained for analysis. Imputation of missing genotypes was performed using IMPUTE software v2.1.0[9] and HapMap release 24, NCBI Build 36.

All participants underwent an ophthalmological investigation of 25 minutes' duration taking place between 11:00 a.m. and 8:00 p.m. This examination was based on standard operating procedures,

including 30° and 45° color photographs by a non-mydriatic fundus camera (Visucam PRO NM,™, Carl Zeiss Meditec AG, Jena, Germany) centered around the optic nerve head (ONH). The vertical cup to disc ratio, disc area, cup area and rim width were measured semiautomatically with Visupac™ (Carl Zeiss Meditec AG, Jena, Germany). At the time of this analysis, ONH data of 1268 genotyped subjects (GHS I: n=783, GHS II: n=485) were available.

Massachusetts Eye and Ear Infirmary (MEEI) glaucoma clinic and *Glaucoma Genes and Environment (GLAUGEN)*

The GLAUGEN study consists of POAG cases and controls drawn from the Nurses' Health Study (NHS), the Health Professionals Follow-up Study (HPFS) and the Genetic Etiologies of Primary Open-Angle Glaucoma study. The former two studies are population-based, nested case-control studies and the latter study is a clinic-based case-control study from the Massachusetts Eye and Ear Infirmary. Details regarding the inclusion/exclusion criteria for the GLAUGEN POAG case-control cohort have been described[20].

491 cases and 351 controls collected from the Massachusetts Eye and Ear Infirmary glaucoma clinic and comprehensive ophthalmology clinics were recruited for this study. All cases and controls were residents of the continental United States and were of mainly European ancestry, which was confirmed by both self-identification and genetic markers. Primary open angle glaucoma (POAG) cases were defined as individuals for whom reliable visual field (VF) tests show characteristic VF defects consistent with glaucomatous optic neuropathy. Individuals were classified as affected if the VF defects were reproduced on a subsequent test or if a single qualifying VF was accompanied by a cup-disc ratio (CDR) of 0.7 or more in at least one eye. The examination of the ocular anterior segment did not show signs of secondary causes for elevated IOP such as exfoliation syndrome or pigment dispersion syndrome and the filtration structures were deemed to be open based on clinical measures. Elevation of IOP was not a criterion for inclusion; however, 67% of cases did have a history of elevated IOP (≥22 mm Hg) measured in a clinical setting (typically between the hours of 8AM and 5PM) and were classified as high-pressure glaucoma.

Genotyping was performed using the Illumina Human660W_Quad_v1 array and 495,132 SNPs passed quality control filters. Illumina‟s BeadStudio and GenomeStudio and Autocall software along with genotype cluster definitions based on study samples were used to generate genotyping calls. SNPs with a GenTrain score <0.6, cluster separation score <0.4 and call rate <97% were considered technical failures at the genotyping center and were automatically deleted before release for further quality control. Subsequent data quality control measures consisted of identifying and removing samples with gender misidentification, unexpected duplicates and unexpected relatedness. Analysis of connectivity removed samples that appeared to be related to other samples and/or suggestive of contamination. Any SNP with missing call rate >2% or with Hardy Weinberg p-value < 10^{-4} in the control population was excluded. Imputation was performed with IMPUTE2 using the March 2012 1000 genomes as a reference panel. Logistic regression analysis was performed using PLINK implementing dosage data analysis options. Covariates in the MEEI analysis included sex, age, DNA source (blood or cheek) and extraction method.

National Eye Institute (NEI) Glaucoma Human Genetics Collaboration (NEIGHBOR)

2,170 cases and 2,347 controls collected from 12 sites throughout the United States were genotyped for the NEIGHBOR study[21]. Primary open angle glaucoma (POAG) cases were defined as individuals for whom reliable visual field (VF) tests show characteristic VF defects consistent with glaucomatous optic neuropathy. Individuals were classified as affected if the VF defects were reproduced on a subsequent test or if a single qualifying VF was accompanied by a cup-disc ratio (CDR) of 0.7 or more in at least one eye. The examination of the ocular anterior segment did not show signs of secondary causes for elevated IOP such as exfoliation syndrome or pigment dispersion syndrome and the filtration structures were deemed to be open based on clinical measures. Elevation of IOP was not a criterion for inclusion; however, 67% of cases did have a history of elevated IOP (≥22 mm Hg) measured in a clinical setting (typically between the hours of 8AM and 5PM) and were classified as high-pressure glaucoma (HPG).

Genotyping was performed using the Illumina Human660W_Quad_v1 array and 523,528 SNPs passed quality control filters. Allele cluster definitions for each SNP were determined using Illumina GenomeStudio Genotyping Module version 1.7.4, GenTrain version 1.0 and the combined intensity data from 99.9% of the samples. The resulting cluster definitions were used on all samples. Genotypes were not called if the quality threshold (Gencall score) was below 0.15. Genotypes were released by CIDR for 557,029 SNPs (99.58% of attempted). Genotypes were not released for SNPs that had call rates less than 85%, more than 1 HapMap replicate error, cluster separation less than 0.2, more than a 3% (autosomal) or 2.2% (X chromosome) difference in call rate between genders, more than 0.4% (X chromosome) male heterozygosity, or more than a 8% (autosomal) difference in AB frequency. Imputation was performed with IMPUTE2 using the March 2012 1000 genomes as a reference panel. Logistic regression using a model included age, gender, study site and 2 eigenvectors (EV1 and 2) was to assess the association between individual SNPs and POAG was done using PLINK v1.07.

Raine Study

The Western Australian Pregnancy Cohort (Raine) Study is an ongoing prospective cohort study of pregnancy, childhood, adolescence and young adulthood in Perth, Western Australia[22]. At the initiation of the study, 2,900 pregnant women were recruited at 16-18 weeks' gestation from the state's largest public women's hospital and surrounding private practices for a randomized clinical trial investigating effects of intensive ultrasound and Doppler studies in pregnancy outcomes. Following this study, the offspring of the recruited individuals have been evaluated in detail during childhood and adolescence. At the 20-year review of the cohort, Raine participants underwent a comprehensive ocular examination for the first time[23]. As part of this examination, a baseline glaucoma analysis was done on each participant using the Heidelberg Retina Tomography 3 (Heidelberg Engineering, Heidelberg, Germany). Participant was appropriately positioned in front of the camera and instructed to stare at the flashing light. The position of the camera was adjusted to illuminate and sharpen the image of the optic disc. Poor images were repeated. Each scan was reviewed at the end and the mean standard deviation of less than 20 µm was maintained for quality check. DNA samples and consents for GWAS studies were available from the previous assessments. Genotype data was generated using the genome-wide Illumina 660 Quad Array at the Centre for Applied Genomics (Toronto, Ontario, Canada). As part of quality control (QC), we investigated for any

individuals who were related with a π > 0.1875 (second or third degree relatives) and excluded the individuals with the higher proportion of missing data. We also excluded people who had a high degree of missing genotyping data (> 3%). The data was filtered for a Hardy-Weinberg equilibrium p-value > 5.7x10-7, SNP call rate >95%, and a minor allele frequency >0.01. We performed the GWAS imputation in the MACH v1.0.16 (http://www.sph.umich.edu/csg/yli/mach/index.html) software using the CEU samples from HapMap phase2 build 36 release 22 (http://hapmap.ncbi.nlm.nih.gov/index.html.en). This study was approved by the Human Research Ethics Committee of the University of Western Australia. The study was conducted in accordance with the Declaration of Helsinki and informed consent was obtained from all participants.

Rotterdam Study (RS-I, RS-II, RS-III)

The Rotterdam Study (RS) is a prospective population-based cohort study in the elderly living in Ommoord, a suburb of Rotterdam, the Netherlands[24]. In brief, the Rotterdam Study consists of 3 independent cohorts: RS-I, RS-II, and RS-III. Participants underwent multiple physical examinations with regular intervals from 1991 to present. The optic nerve head was assessed with ImageNet (RS-I and RS-II) or Heidelberg Retina Tomograph 2 (RS-III). Details of this assessment have been described elsewhere[18]. All measurements in RS-I, RS-II and RS-III were conducted after the Medical Ethics Committee of the Erasmus University had approved the study protocols and all participants had given a written informed consent in accordance with the Declaration of Helsinki. DNA was extracted from blood leucocytes according to standard procedures. Genotyping of SNPs was performed using the Illumina Infinium II HumanHap550 chip v3.0 array (RS-I); the HumanHap550 Duo Arrays and the Illumina Human610-Quad Arrays (RS-II), and the Human 610 Quad Arrays Illumina (RS-III). Samples with low call rate (<97.5%), with excess autosomal heterozygosity (>0.336), or with sex-mismatch were excluded, as were outliers identified by the identity-by-state clustering analysis (outliers were defined as being >3 s.d. from population mean or having identity-by-state probabilities >97%). We used genomic control to obtain optimal and unbiased results and applied the inverse variance method of each effect size estimated for both autosomal SNPs that were genotyped and imputed in both cohorts. A set of genotyped input SNPs with call rate >98%, with minor allele frequency >0.01, and with Hardy-Weinberg P value $>10^{-6}$ was used for imputation. We used the Markov Chain Haplotyping (MACH) package version 1.0.15 software (Rotterdam, The Netherlands; imputed to plus strand of NCBI build 36, HapMap release #22) for the analyses. For each imputed SNP, a reliability of imputation was estimated as the ratio of the empirically observed dosage variance to the expected binomial dosage variance (O/E ratio). GWAS analyses were performed using GRIMP[25].

Singapore (SCES, SIMES, SINDI)

Singapore Malay Eye Study (SIMES) is a population-based prevalence survey of Malay adults aged 40 to 79 years living in Singapore that was conducted between August of 2004 and June of 2006[26]. From a Ministry of Home Affairs random sample of 16,069 Malay adults in the Southwestern area, an age-stratified random sampling strategy was used in selecting 1400 from each decade from age 40 years onward (40–49, 50–59, 60–69, and 70–79 years).The 4,168 eligible participants from the sampling frame, while 3280 (78.7%) participated. Genome-wide genotyping was performed in 3,072 individuals[12,27]. Total of 3,072 DNA samples were genotyped using the Illumina Human 610 Quad Beadchips[27,28]. Using the same quality control criteria, we omitted a total of 530 individuals including those of subpopulation structure (n=170), cryptic relatedness (n=279), excessive heterozygosity or

high missingness rate > 5% (n=37), and gender discrepancy (n=44). After the removal of the samples, SNP QC was then applied on a total of 579,999 autosomal SNPs for the 2,542 post-QC samples. SNPs were excluded based on (i) high rates of missingness (> 5%) ; (ii) monomorphism or MAF < 1% ; or (iii) genotype frequencies deviated from HWE ($p < 1 \times 10^{-6}$).

Singapore Indian Eye Study (SINDI) is a population-based survey of major eye diseases[29] in ethnic Indians aged 40 to 80 years living in the South-Western part of Singapore and was conducted from August 2007 to December 2009. In brief, 4,497 Indian adults were eligible and 3,400 participated. Genome-wide genotyping was performed in 2,953 individuals[28]. The Illumina Human610 Quad Beadchips was used for genotyping all DNA samples from SINDI (n=2,593). We excluded 415 subjects from the total of 2,953 genotyped samples based on: excessive heterozygosity or high missingness rate > 5% (n=34) , cryptic relatedness (n=326), issues with population structure ascertainment (n=39) and gender discrepancies (n=16). This left a total of 2,538 individuals with 579,999 autosomal SNPs. During SNP QC procedure. SNPs were excluded based on (i) high rates of missingness (> 5%) ; (ii) monomorphism or MAF < 1% ; or (iii) genotype frequencies deviated from HWE ($p < 1 \times 10^{-6}$).

Similar to SINDI, the Singapore Chinese Eye Study (SCES) is a population-based cross-sectional study of eye diseases in Chinese adults 40 years of age or older residing in the southwestern part of Singapore. The methodology of the SCES study has been described in details previously. Between 2009 and 2011, 3,353 (72.8%) of 4,605 eligible individuals underwent a comprehensive ophthalmologic examination, using the same protocol as SINDI[26]. Genome-wide genotyping using was done in a subset of 1,952 SCES participants using Illumina Human610-Quad BeadChip[12]. The same QC methods used for SIMES and SINDI were applied to the SCES genotyping samples: samples were excluded if they showed evidence of admixture, cryptic relatedness, high heterogeneity and gender discrepancies. From a starting number of 1,952 individuals, three samples had per-sample call rate of <95% and were removed from analysis. A total of 21 individuals showed evidence of admixture and were consequently excluded. Biological relationship verification revealed a total of 29 sample pairs with cryptic relatedness. For these, the sample with the lower call rate was removed. In addition, further 14 samples with impossible biological sharing or heterogeneity, probably because of contamination, were removed, as well as two individuals who were removed due to gender discrepancies. PC analysis of the remaining individuals for SCES against the HapMap CHB (Han Chinese) reference populations did not show the cohort to be dissimilar in ancestry, and therefore no PCs were used to correct for any underlying population substructure in the analysis performed.

Optic disc parameters were measured using Heidelberg Retina Tomography 2 (HRT 2), as previously described[30,31] for SIMES, SINDI and SCES.

All three studies adhere to the Declaration of Helsinki. Ethics approvals have been obtained from the Institutional Review Boards of the Singapore Eye Research Institute, Singapore General hospital, National University of Singapore and National Healthcare Group, Singapore. In all cohorts, participants provided written, informed consent at the recruitment into the studies. For studies involving children (SCORM), written informed consent was obtained from the children’s parents.

Southampton

Primary open-angle (POAG) and normal tension glaucoma patients were recruited from the Southampton University Hospital Trust Eye Clinic and satellite regional glaucoma clinics. Ethical approval for the collection of patient information and blood samples was provided by the Southampton and South West Hampshire Local Research Ethics Committee (05/Q1702/8) and Cohort Recruitment commenced in August 2005. Each patient was examined by an experienced glaucoma specialist. Diagnoses were made on the basis of characteristic visual field loss/glaucomatous optic disc damage/increased IOP. Patients presenting with narrow-angle, developmental or secondary glaucoma or any other known abnormalities of the anterior segment were excluded. Patients with unambiguous glaucoma, but normal tension were included in sample collection later. Furthermore, to select for patients with typical POAG or normal-tension glaucoma (NTG), only patients diagnosed over the age of 40 years were included. Both conditions are rare before this age. DNA was extracted according to the standard methods, dissolved in TE buffer, and stored at -20°C. Primary open angle glaucoma patients (n=400) were genotyped on the Affymetrix SNP 6.0 array, all data were exported on the forward strand. These data were compared with the Affymetrix SNP 6.0 data publically available for the WTCCC2 controls. Quality control steps involved removing cases and SNPs with a high degree of missingness (>10%), and removing SNPs with a minor allele frequency less than 5%. We also carried out identity by state (IBS) analysis to identify unknown relatives or duplicates and multi-dimensional scaling (MDS) to identify those with differing ethnic backgrounds to the majority of the group (a Caucasian cohort). 387 cases were available for analysis after this step. The HWE test (in controls) is generally used to control for genotyping error when cases and controls are genotyped together. However as the cases were genotyped separately extra QC steps were carried out. Firstly, the average confidence score for the genotypes (from the Affymetrix SNP calling software "Genotyping console") were calculated for each SNP across individuals and SNPs with confidence scores which fell two standard deviations above the mean (worse confidence) were removed. We also removed SNPs with extreme deviations from HWE ($p<1x10^{-10}$) in cases. Three known tri-allelic SNPs were also removed. 681,549 SNPs were available for analysis after QC. These data were published earlier[32].

Twins Eye Study in Tasmania (TEST) and *Brisbane Adolescent Twin Study (BATS)*

The Australian Twin Eye Study comprises participants examined as part of the Twins Eye Study in Tasmania or the Brisbane Adolescent Twins Study. Details of the study are described elsewhere[33]. Ethical approval was obtained from the Royal Victorian Eye and Ear Hospital, the University of Tasmania, the Australian Twin Registry and the Queensland Institute of Medical Research. A Nidek 3-Dx fundus camera (Nidek, Gamagori, Japan) was used to obtain simultaneous stereoscopic optic disc photographs. All images were captured on colour 35 mm slides (Ektachrome, Eastman Kodak, Rochester, NY, USA) and digitized using a Nikon CoolScan IV ED slide scanner (Nikon Corp., Tokyo, Japan). Optic discs were analysed stereoscopically with custom planimetric software (StereoDx, using a Z-screen; StereoGraphics Corp., Beverly Hills, CA, USA), where the inner margin of the optic disc and the neuroretinal rim were delineated at the depth of the scleral plane, and images were modified for magnification using refraction and keratometry data. DNA was extracted from blood leucocytes according to standard procedures. The Australian cohorts were genotyped on the Illumina Human Hap610 Quad array. SNPs with a genotype success rate of 0.95 or above was required for inclusion of the SNP into further steps of the analysis. Only SNPs in Hardy-Weinberg equilibrium were

processed: the HWE inclusion threshold was P>10x10^{-6}. The minimum minor allele frequency required for inclusion of individual SNPs was 0.01. Imputation was calculated with reference to HapMap release 22 CEU using MACH (http://www.sph.umich.edu/csg/abecasis/MACH/). Association analysis was performed using Merlin (http://www.sph.umich.edu/csg/abecasis/merlin/) in the Australian twin data. Ancestry for these individuals was determined initially through self-reporting and was verified through Principal Component decomposition of their ancestry with and without comparison with HapMap phase 2 standard populations.

TwinsUK

The TwinsUK adult twin registry based at St. Thomas' Hospital in London is a volunteer cohort of over 10,000 twins from the general population[34]. Twins largely volunteered unaware of the eye studies, gave fully informed consent under a protocol reviewed by the St. Thomas' Hospital Local Research Ethics Committee. Out of the original 1,951 subjects for whom phenotype and genotype information was available, 1,922 subjects were included in the study; 29 subjects were excluded after failing quality control. Genotyping was carried out using three genotyping platforms from Illumina: the HumanHap 300k Duo for part of the UK Twin Cohort and the HumanHap610-Quad array for the rest of the UK Twin Cohort. Imputation was calculated with reference to HapMap release 22 CEU population data using IMPUTE version 2. Individuals were included if their genotyping success rate exceeded 95%, did not show excess or low heterozygosity (defined by the interval of 0.2-04). SNPs were included in the imputation if they had a genotype success rate of at least 0.95 if their minor allele frequency was superior to 0.005 and at least 0.99 if their MAF was 0.01-0.05. Only SNPs that were within Hardy-Weinberg equilibrium ($p>10^{-4}$) and had a minor allele frequency of 0.04 or above were regressed. VCDR in the subjects was measured from stereo disc photographs using the Nidek-3DX stereo camera, with digitized images scanned from Polaroid images and StereoDx stereoscopic planimetric software (StereoDx) using a Z-screen (StereoGraphics Corp) and software obtained from James Morgan from Cardiff University software, Wales, UK[35].

Supplementary References

1. Consortium, E.P. A user's guide to the encyclopedia of DNA elements (ENCODE). *PLoS Biol* **9**, e1001046 (2011).
2. Young, T.L. et al. Whole Genome Expression Profiling of Normal Human Fetal and Adult Ocular Tissues. *Exp Eye Res* **116**, 265-78 (2013).
3. Booij, J.C. et al. Functional annotation of the human retinal pigment epithelium transcriptome. *BMC Genomics* **10**, 164 (2009).
4. Janssen, S.F. et al. Gene expression and functional annotation of the human ciliary body epithelia. *PLoS One* **7**, e44973 (2012).
5. Bowes Rickman, C. et al. Defining the human macula transcriptome and candidate retinal disease genes using EyeSAGE. *Invest Ophthalmol Vis Sci* **47**, 2305-16 (2006).
6. Liu, Y. et al. Serial analysis of gene expression (SAGE) in normal human trabecular meshwork. *Mol Vis* **17**, 885-93 (2011).
7. Wagner, A.H. et al. Exon-level expression profiling of ocular tissues. *Exp Eye Res* **111**, 105-11 (2013).
8. Burdon, K.P. et al. Genome-wide association study identifies susceptibility loci for open angle glaucoma at TMCO1 and CDKN2B-AS1. *Nat Genet* **43**, 574-8 (2011).
9. Howie, B.N., Donnelly, P. & Marchini, J. A flexible and accurate genotype imputation method for the next generation of genome-wide association studies. *PLoS Genet* **5**, e1000529 (2009).
10. Xu, L. et al. Visual acuity in northern China in an urban and rural population: the Beijing Eye Study. *Br J Ophthalmol* **89**, 1089-93 (2005).
11. Xu, L., Zhang, H., Wang, Y.X. & Jonas, J.B. Central corneal thickness and glaucoma in adult Chinese: the Beijing Eye Study. *J Glaucoma* **17**, 647-53 (2008).
12. Cornes, B.K. et al. Identification of four novel variants that influence central corneal thickness in multi-ethnic Asian populations. *Hum Mol Genet* **21**, 437-45 (2012).
13. van Koolwijk, L.M. et al. Major genetic effects in glaucoma: commingling analysis of optic disc parameters in an older Australian population. *Invest Ophthalmol Vis Sci* **50**, 5275-80 (2009).
14. Jonasson, F. et al. Prevalence of open-angle glaucoma in Iceland: Reykjavik Eye Study. *Eye (Lond)* **17**, 747-53 (2003).
15. Styrkarsdottir, U. et al. Nonsense mutation in the LGR4 gene is associated with several human diseases and other traits. *Nature* **497**, 517-20 (2013).
16. Aulchenko, Y.S. et al. Linkage disequilibrium in young genetically isolated Dutch population. *Eur J Hum Genet* **12**, 527-34 (2004).
17. Pardo, L.M., MacKay, I., Oostra, B., van Duijn, C.M. & Aulchenko, Y.S. The effect of genetic drift in a young genetically isolated population. *Ann Hum Genet* **69**, 288-95 (2005).
18. Ramdas, W.D. et al. A genome-wide association study of optic disc parameters. *PLoS Genet* **6**, e1000978 (2010).
19. Zeller, T. et al. Genetics and beyond--the transcriptome of human monocytes and disease susceptibility. *PLoS One* **5**, e10693 (2010).
20. Wiggs, J.L. et al. Common variants near CAV1 and CAV2 are associated with primary open-angle glaucoma in Caucasians from the USA. *Hum Mol Genet* **20**, 4707-13 (2011).
21. Wiggs, J.L. et al. The NEIGHBOR Consortium Primary Open-Angle Glaucoma Genome-wide Association Study: Rationale, Study Design, and Clinical Variables. *J Glaucoma* (2012).
22. McKnight, C.M. et al. Birth of a cohort--the first 20 years of the Raine study. *Med J Aust* **197**, 608-10 (2012).
23. Yazar, S. et al. Raine Eye Health Study: Design, Methodology and Baseline Prevalence of Ophthalmic Disease in a Birth-cohort Study of Young Adults. *Ophthalmic Genet* (2013).
24. Hofman, A. et al. The Rotterdam Study: 2014 objectives and design update. *Eur J Epidemiol* **28**, 889-926 (2013).
25. Estrada, K. et al. GRIMP: a web- and grid-based tool for high-speed analysis of large-scale genome-wide association using imputed data. *Bioinformatics* **25**, 2750-2 (2009).

26. Foong, A.W. et al. Rationale and methodology for a population-based study of eye diseases in Malay people: The Singapore Malay eye study (SiMES). *Ophthalmic Epidemiol* **14**, 25-35 (2007).
27. Vithana, E.N. et al. Collagen-related genes influence the glaucoma risk factor, central corneal thickness. *Hum Mol Genet* **20**, 649-58 (2011).
28. Khor, C.C. et al. Genome-wide association studies in Asians confirm the involvement of ATOH7 and TGFBR3, and further identify CARD10 as a novel locus influencing optic disc area. *Hum Mol Genet* **20**, 1864-72 (2011).
29. Lavanya, R. et al. Methodology of the Singapore Indian Chinese Cohort (SICC) eye study: quantifying ethnic variations in the epidemiology of eye diseases in Asians. *Ophthalmic Epidemiol* **16**, 325-36 (2009).
30. Zheng, Y. et al. Influence of diabetes and diabetic retinopathy on the performance of Heidelberg retina tomography II for diagnosis of glaucoma. *Invest Ophthalmol Vis Sci* **51**, 5519-24 (2010).
31. Zheng, Y. et al. Diagnostic ability of Heidelberg Retina Tomography in detecting glaucoma in a population setting: the Singapore Malay Eye Study. *Ophthalmology* **117**, 290-7 (2010).
32. Gibson, J. et al. Genome-wide association study of primary open angle glaucoma risk and quantitative traits. *Mol Vis* **18**, 1083-92 (2012).
33. Mackey, D.A. et al. Twins eye study in Tasmania (TEST): rationale and methodology to recruit and examine twins. *Twin Res Hum Genet* **12**, 441-54 (2009).
34. Spector, T.D. & Williams, F.M. The UK Adult Twin Registry (TwinsUK). *Twin Res Hum Genet* **9**, 899-906 (2006).
35. Morgan, J.E., Sheen, N.J., North, R.V., Choong, Y. & Ansari, E. Digital imaging of the optic nerve head: monoscopic and stereoscopic analysis. *Br J Ophthalmol* **89**, 879-84 (2005).
